# Supplementary material for: Amino acids catalyse RNA formation under ambient alkaline conditions
Source: Nat Commun. 2025 Jun 4;16:5193. doi: 10.1038/s41467-025-60359-3 (PMC12137669; doi:10.1038/s41467-025-60359-3)
Supplement: Supplementary file 1 — Supplementary Information [file 41467_2025_60359_MOESM1_ESM.pdf]

Supplementary Information for

Amino acids catalyse RNA formation under ambient alkaline conditions

Saroj K. Rout[1,5], Sreekar Wunnava[1], Miroslav Krepl[2], Giuseppe Cassone[3],
Judit E. Šponer[2], Christof B. Mast[1], Matthew W. Powner[4] and Dieter Braun[1*]

[1]Systems Biophysics and Center for NanoScience, Faculty of Physics, Ludwig-Maximilians-Universität München, Geschwister-Scholl-Platz 1, 80539 Munich, Germany

[2]Institute of Biophysics of the Czech Academy of Sciences, Kralovopolska 135, 61200 Brno, Czech Republic

[3]Institute for Chemical-Physical Processes, National Research Council of Italy (IPCF-CNR), 98158 Messina, Italy

[4]Department of Chemistry, University College London, London WC1H 0AJ, U.K.

[5]Present address: Institute of Molecular Physical Science, ETH Zurich, 8093 Zurich, Switzerland

**Correspondence to: dieter.braun@lmu.de*

Contents

- Supplementary Figures S1-S39
- Supplementary Table S1
- Materials and methods (Supplementary Figure S40)

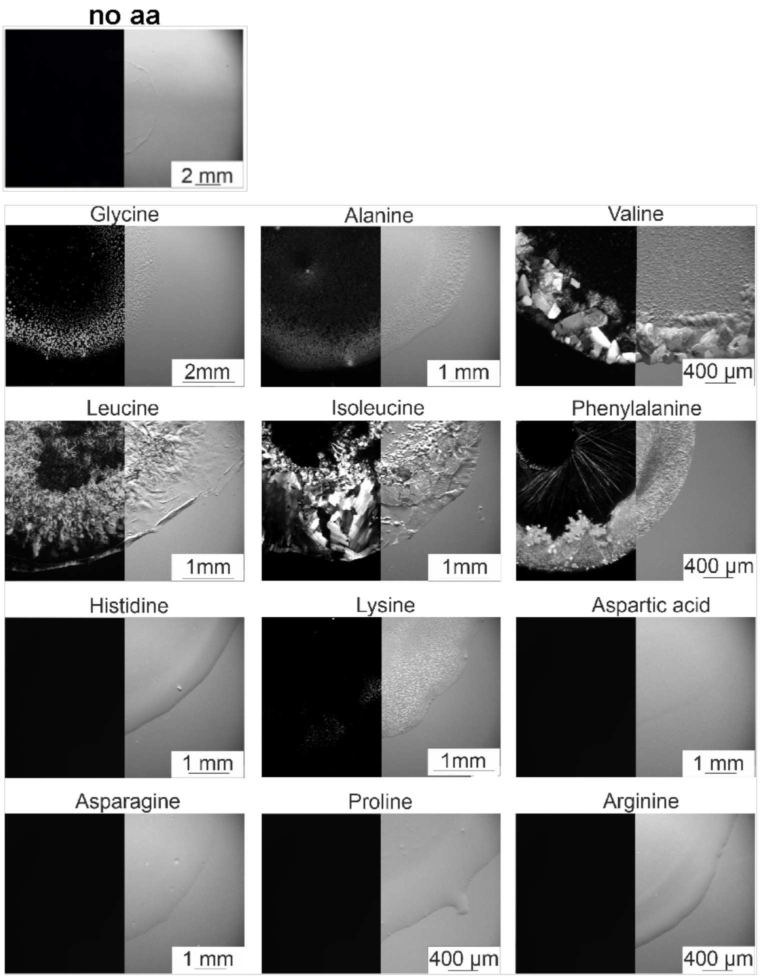

Figure S1. Morphological characterisation of the dried mixtures of nucleotides and amino acids by optical microscopy. The solution containing cCMP and amino acids mixed in a 1:5 ratio was prepared at pH 10 and then incubated for 20 hours. The left half of the images show the internal structure of the samples viewed in cross-polarized light. In the experiment 100 nmol of nucleotides and 500 nmol of amino acids were used. The figures display only a zoomed-in section of the entire dried spot, revealing heterogeneity in its appearance.

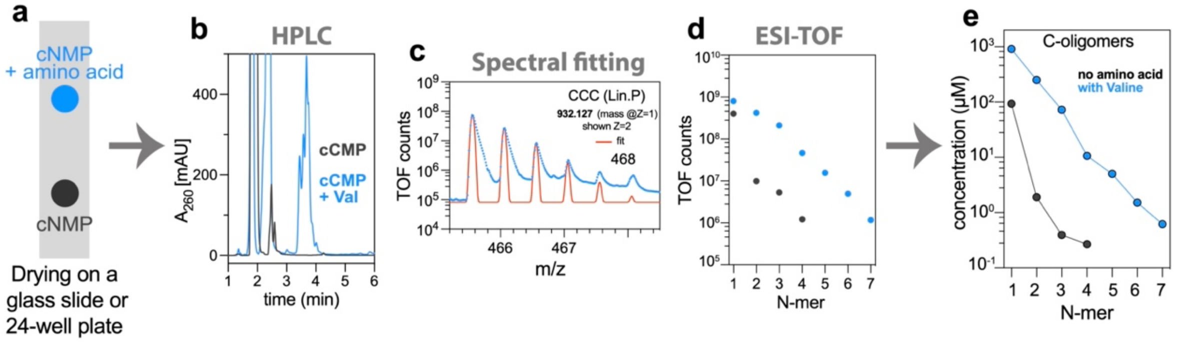

Figure S2. Summary of the experiment and analysis pipeline. a, Drying of the cyclic nucleotides (cCMP) in the absence and presence of amino acids (valine). **b**, Chromatogram at 260 nm is presented for cCMP oligomerisation. **c**, Isotope fits for CCC (-2 charged state) in the

Spectral_browser v3.58. **d,** Total ion counts of each oligomer after spectral fitting. **e,** Concentration of each oligomer as determined by standard curve. Details of the MS data analysis workflow are provided in the Methods section (see Figure S40).

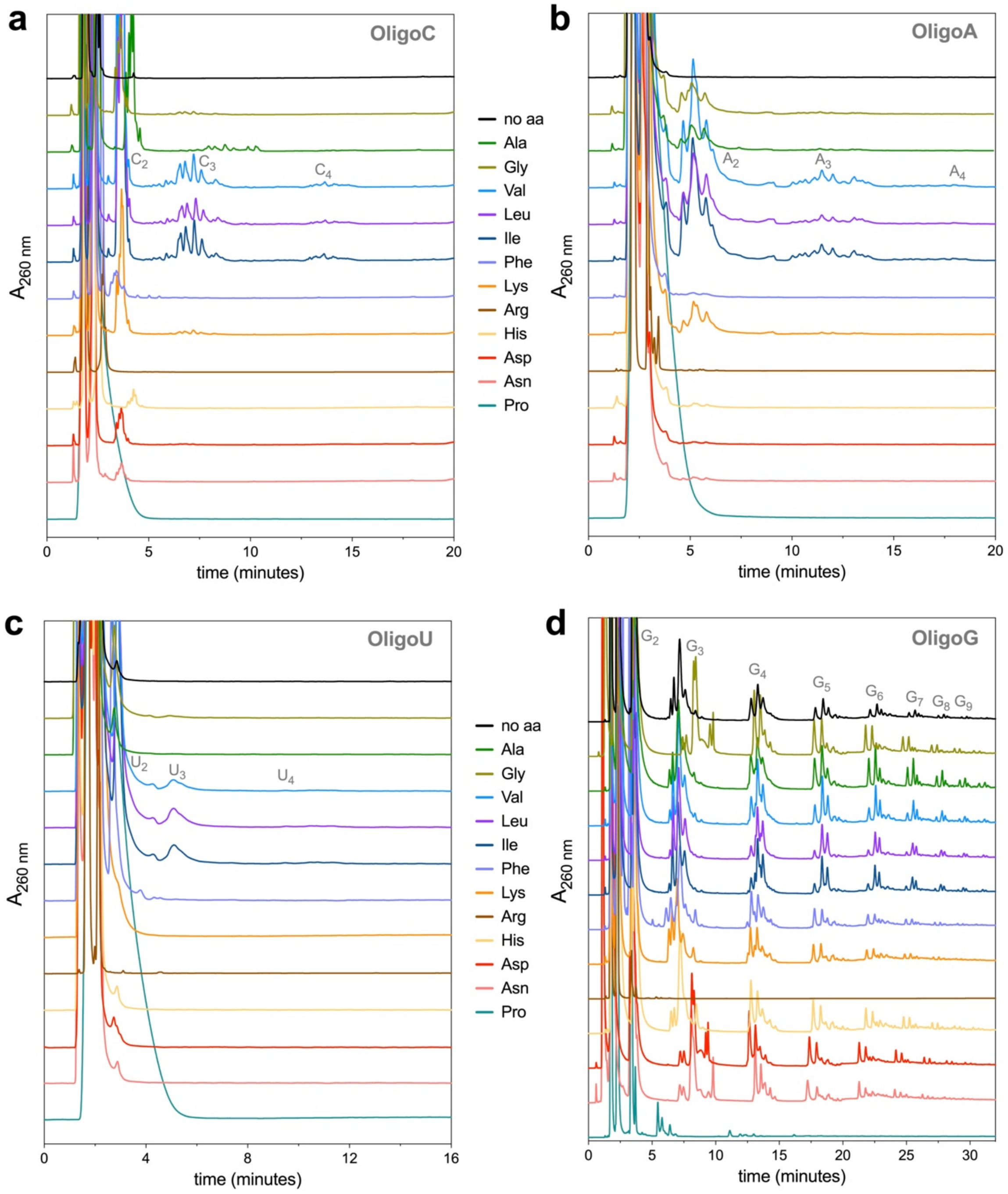

Figure S3. Analysis of the oligomeric RNA products by HPLC. a, The HPLC traces of oligoC at 260 nm in the absence and presence of amino acids are presented. In the experiments, 10 mM cyclic nucleotides and 50 mM amino acids at pH 10 were rapidly dried and incubated for 20 h at room temperature. **b-d**, Chromatograms for oligoA, oligoU and oligoG are plotted. The elution times of different oligomeric RNA are noted on the plot. All quantitative estimations were done by ESI-TOF and a custom-written LabVIEW program.

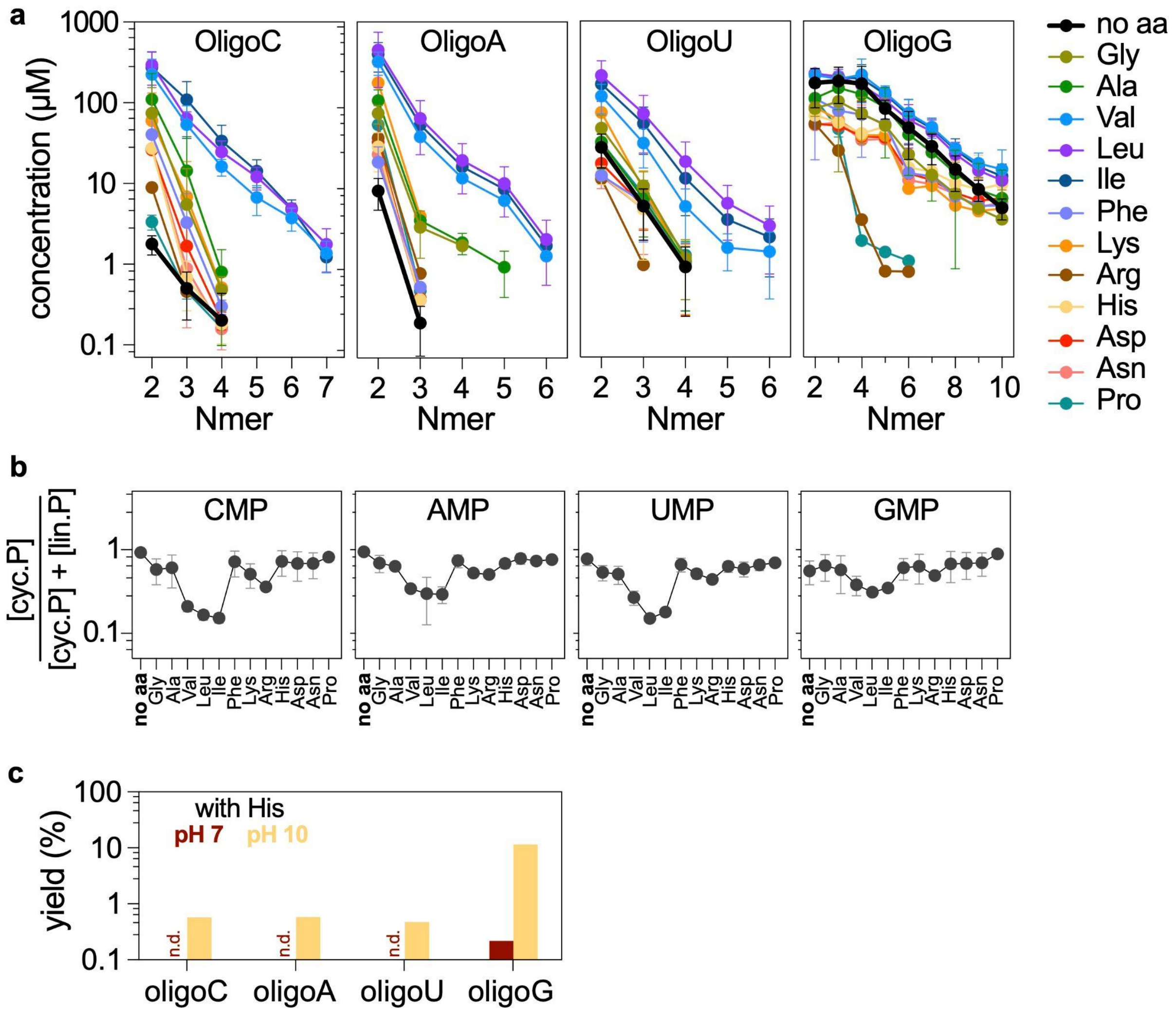

Figure S4. RNA oligomerisation of 2',3'-cyclic nucleotides catalysed by amino acids. a, The total concentrations of oligomers of different lengths, with both linear and cyclic phosphate ends, are plotted on a logarithmic scale for cCMP, cAMP, cUMP, and cGMP oligomerisation**.** Note that we replot the data in Figure 1b but with error bars here. Controls without amino acids are shown in black (no aa), and amino acid-promoted reactions (corresponding three-lettered codes) are shown in other colours. **b,** The ratio of the concentration of 2',3'-cyclic nucleotide monomers to that of the sum of cyclic and linear monomer forms is shown for all four nucleotide types in the presence of amino acids after 20 hours of reaction, providing insights into monomer polymerization and hydrolysis by the amino acids. Nucleotides with 2',3'-cyclic phosphate ends are referred to as cyc.P, while those with hydrolysed 2' or 3' linear ends are denoted as lin.P. **c,** %-yields of RNA oligomers in the presence of histidine at pH 7 and 10. Since histidine contains an ionisable imidazole side chain with a pKa of around 7, we additionally examined its effect on RNA oligomerisation at pH 7 (dark red). At this pH, no oligomerisation was detected for cC/A/UMP, as indicated by "n.d." (not detected), and the yield for G was only 0.2%. At pH 10, the %-yields of RNA (from Figure S4a) are shown in yellow. All reactions were performed with 10 µL of 10 mM cNMP and 50 mM amino acid, at pH 10 (also at pH 7 with His) for 20 h, and quantifications were done on a reverse phase HPLC column coupled to ESI-TOF and by a custom-written LabVIEW program. Errors are given as S.D. of three independent experiments.

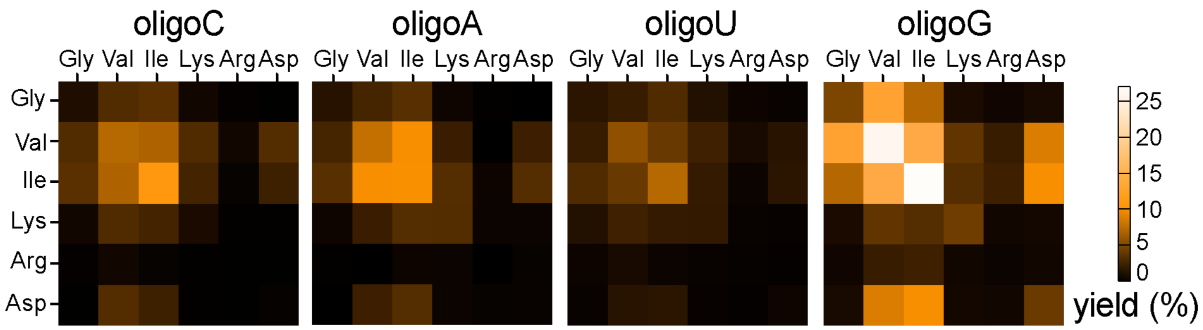

Figure S5. Effect of amino acid mixtures on dry state oligomerisation of RNA from nucleoside 2',3'-cyclic phosphates. The RNA oligomer yields were probed with a 1:1 binary mixture of amino acids (2.5 equiv. each). Maximal yields were typically observed with a single amino acid at the diagonal. The hydrophobic amino acids (Val/Ile), when combined with poorly performing amino acids (Gly/Lys/Arg/Asp), showed a beneficial influence on RNA oligomerisation for all bases. All reactions were performed with 10 µl of 10 mM cNMP and 50 mM amino acid at pH 10 for 20 h, and yields were quantified by reverse phase HPLC ESI-TOF mass spectrometry, fitting the isotope patterns by a custom-written LabVIEW program.

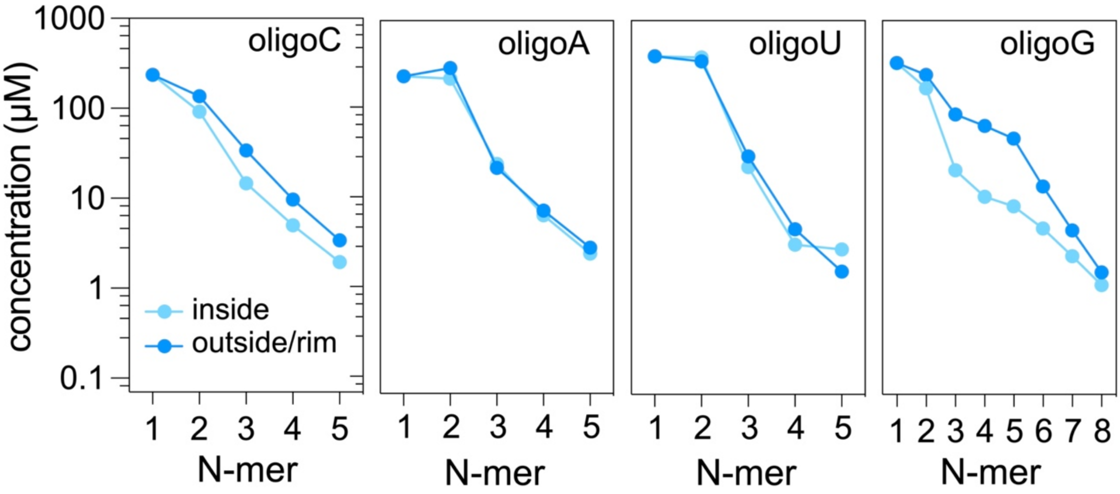

Figure S6. Estimation of oligomers from the outer and inner area of the dried nucleotide and valine mixtures. Reactions were done using 10 mM nucleotides and 50 mM valine at pH 10 by drying for 20 h. The rim of the spot was carefully scraped and separated from the central area. Both samples were then dissolved in nuclease-free water for the LC-MS analysis. Despite some heterogeneity in appearance (see valine in Fig. S1), the oligomerisation process appears to be relatively consistent across the entire spot.

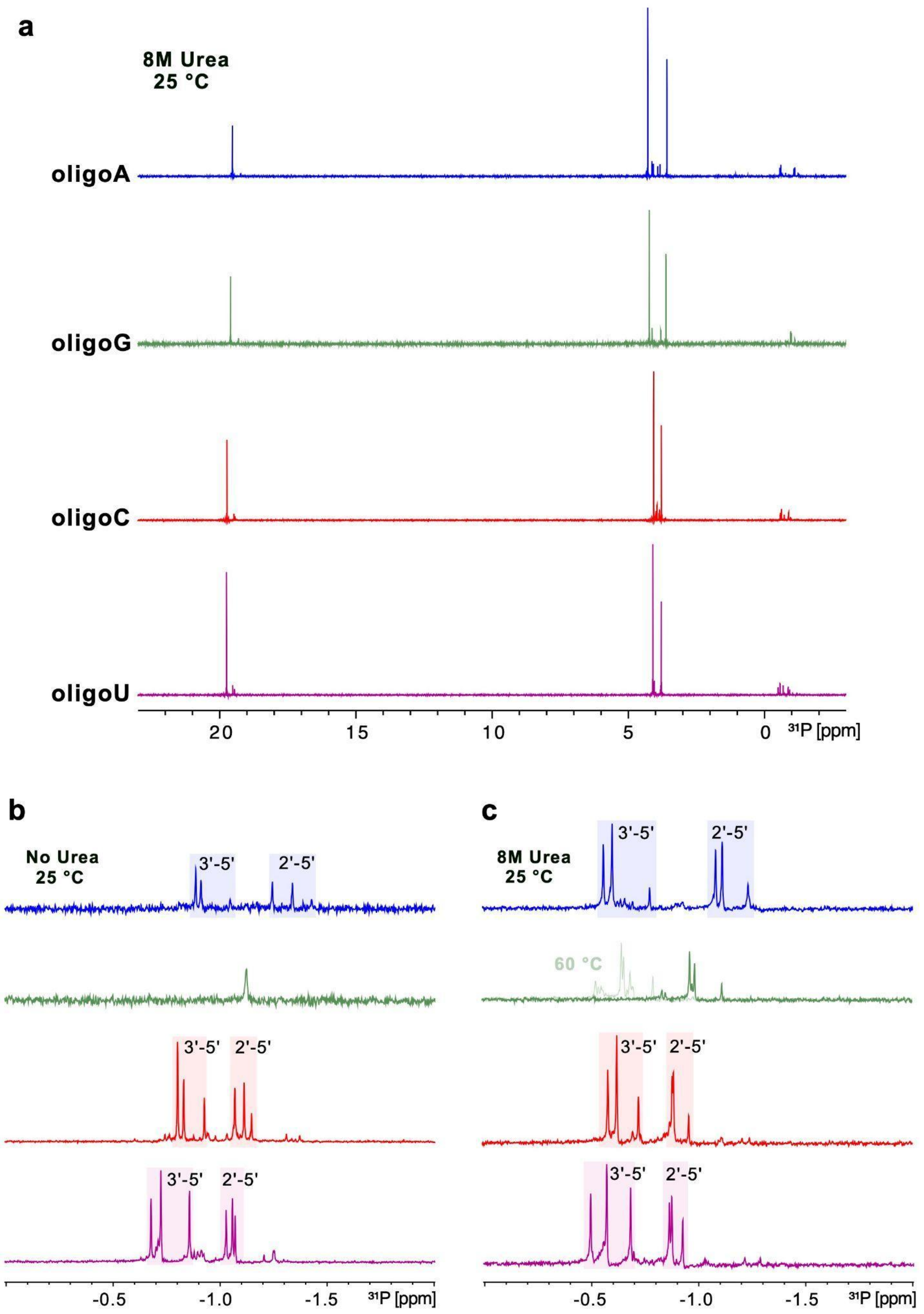

Figure S7. ^{31}P NMR spectra of RNA oligomers. a, NMR spectra (121.5 MHz, H_2O/D_2O 95:5, 23 – -3 ppm) to display all constituents of the reaction of cNMP (ribonucleoside 2', 3'-cyclic phosphate) in the presence of 5 equiv. valine at pH 10 following drying under airflow and incubation at room temperature for 20 h. The samples were dissolved in 5% D_2O/H_2O and also with 8M urea containing 5% D_2O for analysis. The spectra were acquired on a Bruker

Avance III 300 MHz spectrometer and the peak assignments were performed following SI Ref 3. The signals between -1.5 – -0.5 ppm indicate predominantly dinucleotide products with phosphodiester bonds. Hydrolysed cyclic mononucleotides are observed around 4 ppm as 2'- and 3'- monophosphates. A significant amount of unreacted cNMP is detected near 20 ppm. The signals around 19.5 ppm correspond to dinucleotide products with a cyclic phosphate ending. **b-c**, Zoomed-in NMR spectra from -1.5 – -0.5 ppm highlight the 3'-5' and 2'-5' phosphodiester-linked products of the cNMP reaction. Three peaks in this region represent the reaction products, with a 2',3'-cyclic phosphate end and hydrolysed 2'- or 3'-linear phosphate ends.

*oligoG forms aggregates that were not observed by NMR. The NMR signal was improved in 8 M urea and at 60 °C (light green in panel c); however, these spectra were not used to measure the linkage ratio.

The significantly higher peaks of the 2' or 3' linear phosphates (around 4 ppm) compared to those representing phosphodiester linkages (-1.5 to -0.5 ppm) suggest a lower oligomerization yield, likely due to the increased hydrolysis of cyclic nucleotides. Unlike quantitative mass spectrometry, which accounts for all possible oligomer lengths in yield calculations, NMR signals for phosphodiester-linked products primarily reflect dimers within the oligomer pool. Additionally, the oligomerization yield of approximately 8% for A, C, and U in the presence of valine (Mass spectrometry data) indicates that a considerable portion of monomers remained unreacted.

The ratio of 3'-5' to 2'-5' linkages observed in the RNA products were as follows.

	Ratio of **3'-5'** to **2'-5'** linkages	
	**(b)** no Urea	**(c)** with Urea
**oligoA**	55:45	58:42
**oligoG***	-	-
**oligoC**	55:45	58:42
**oligoU**	64:36	66:34

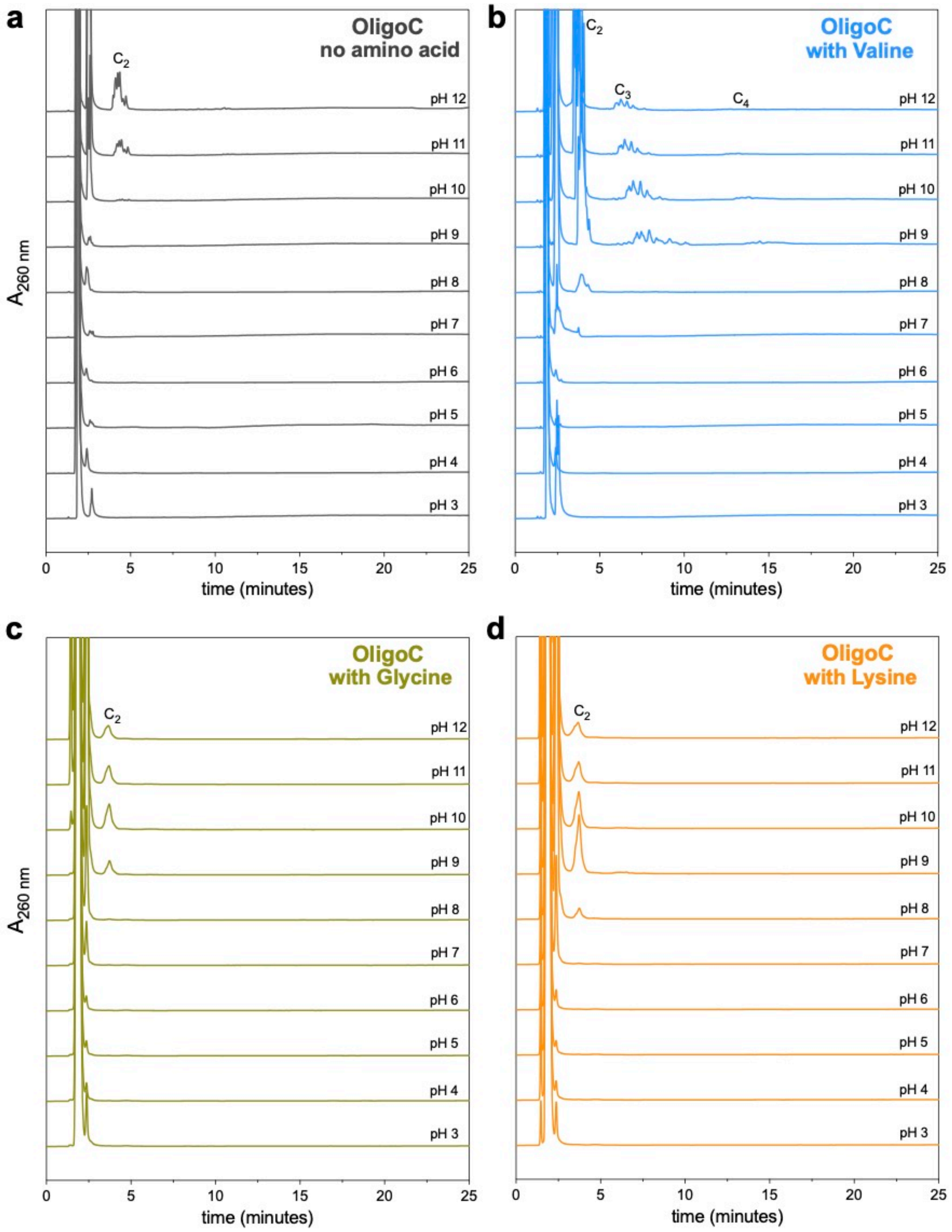

Figure S8. pH-dependent oligomerisation of cCMP in the presence of amino acids investigated by HPLC. a, The HPLC traces of C-oligomerization across pH 3-12, recorded at 260 nm. **b-d**, Chromatograms for oligoC in the presence of valine, glycine and lysine are presented, similar to **a**. In the experiments, 10 mM cyclic nucleotides and 50 mM amino acids at pH 3-12 were rapidly dried and incubated for 20 h at room temperature. The elution times of different oligomeric RNAs are indicated on the plot. All quantitative estimations were done by ESI-TOF and a custom written LabVIEW program.

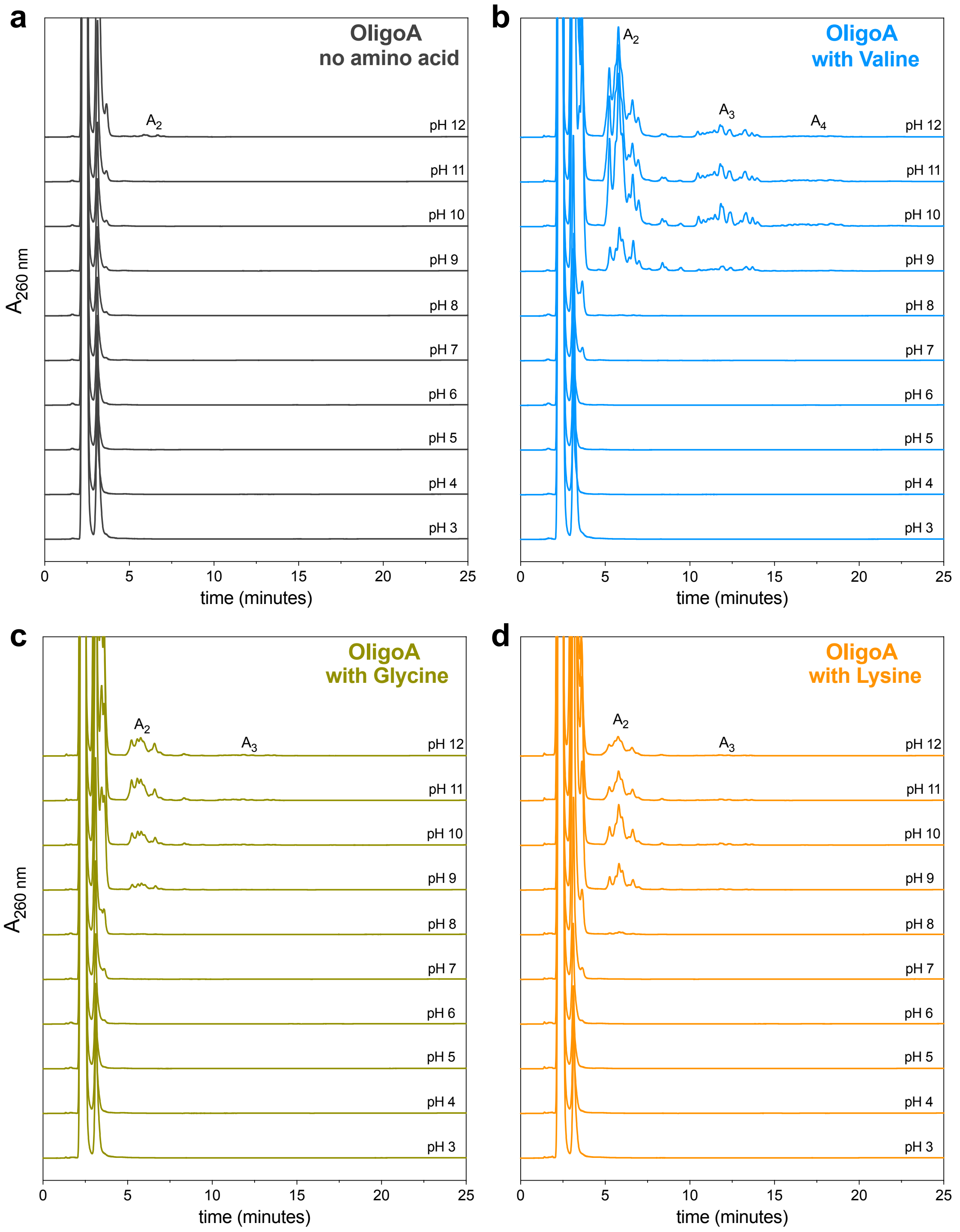

Figure S9. pH-dependent oligomerisation of cAMP in the presence of amino acids investigated by HPLC. a, The HPLC traces of A-oligomerization across pH 3-12, recorded at 260 nm. **b-d**, Chromatograms for oligoA in the presence of valine, glycine and lysine are presented, similar to **a**. In the experiments, 10 mM cyclic nucleotides and 50 mM amino acids at pH 3-12 were rapidly dried and incubated for 20 h at room temperature. The elution times of different oligomeric RNAs are indicated on the plot. All quantitative estimations were done by ESI-TOF and a custom-written LabVIEW program.

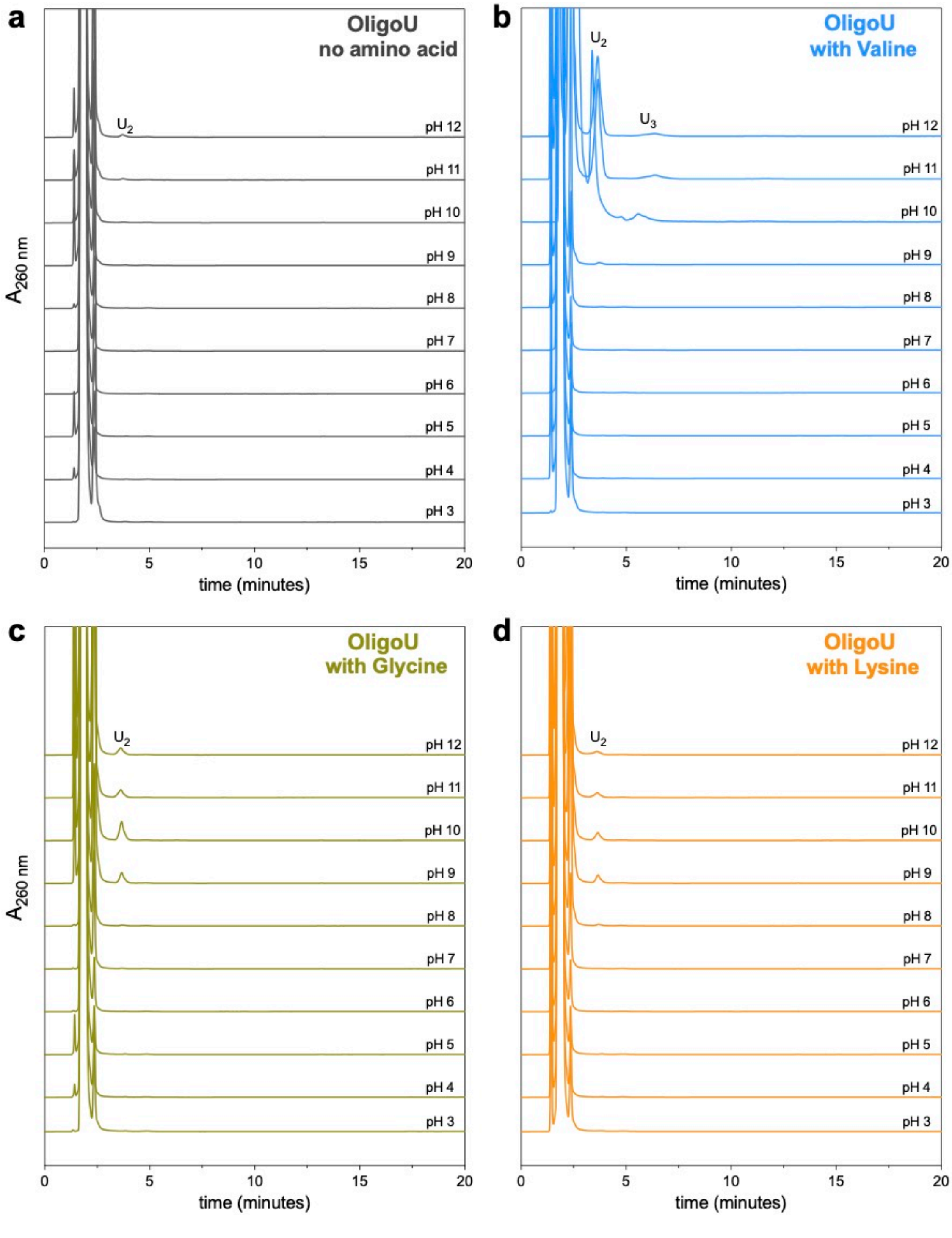

Figure S10. pH-dependent oligomerisation of cUMP in the presence of amino acids investigated by HPLC. a, The HPLC traces of U-oligomerization across pH 3-12, recorded at 260 nm. **b-d**, Chromatograms for oligoU in the presence of valine, glycine and lysine are presented, similar to **a**. In the experiments, 10 mM cyclic nucleotides and 50 mM amino acids at pH 3-12 were rapidly dried and incubated for 20 h at room temperature. The elution times of different oligomeric RNAs are indicated on the plot. All quantitative estimations were done by ESI-TOF and a custom-written LabVIEW program.

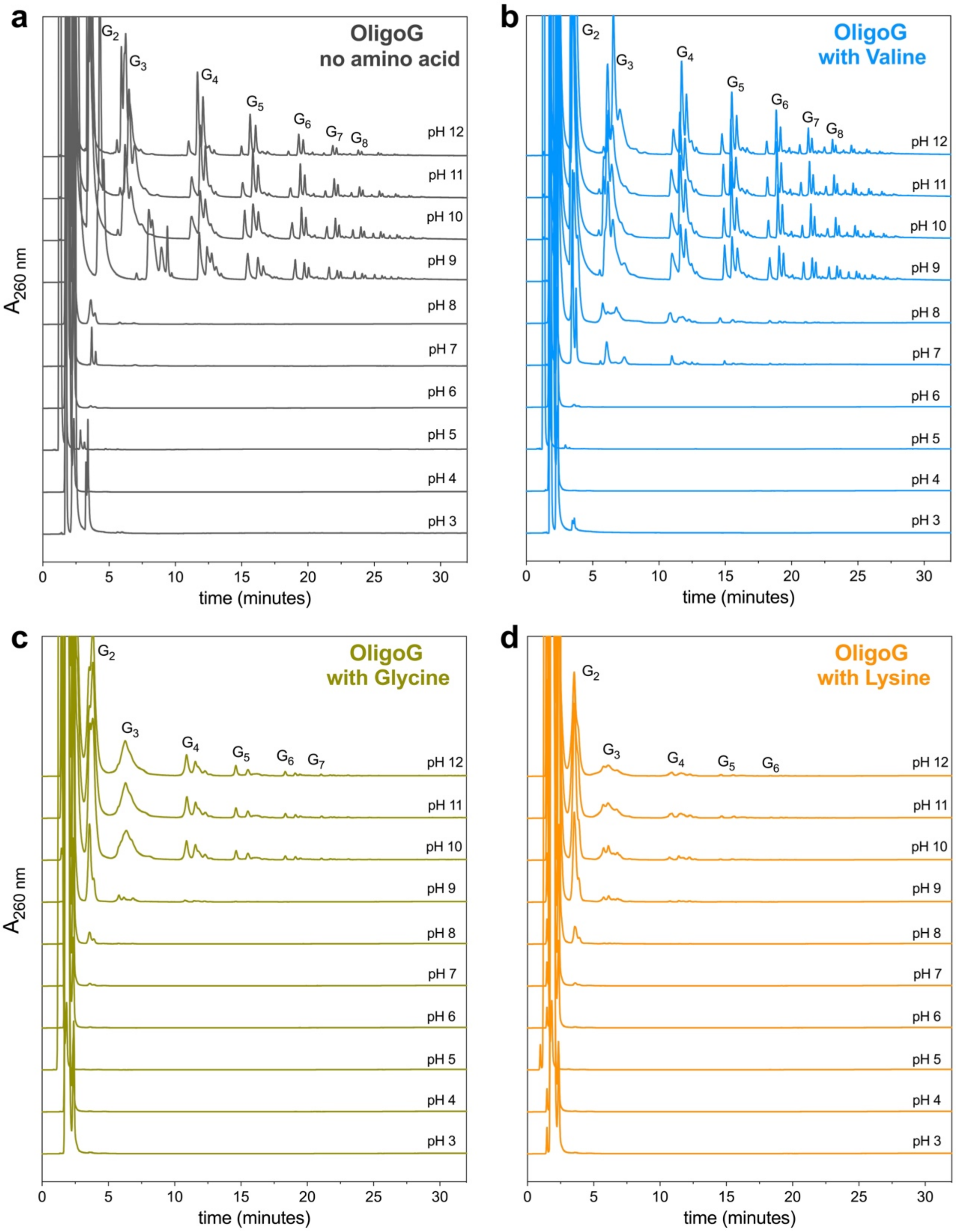

Figure S11. pH-dependent oligomerisation of cGMP in the presence of amino acids investigated by HPLC. a, The HPLC traces of G-oligomerization across pH 3-12, recorded at 260 nm. **b-d**, Chromatograms for oligoG in the presence of valine, glycine and lysine are presented, similar to **a**. In the experiments, 10 mM cyclic nucleotides and 50 mM amino acids at pH 3-12 were rapidly dried and incubated for 20 h at room temperature. The oligomers containing a mixture of canonical and non-canonical linkages, along with linear and cyclic phosphate ends, elute slightly differently on the HPLC columns, resulting in multiple peaks. All quantitative estimations were done by ESI-TOF and a custom written LabVIEW program.

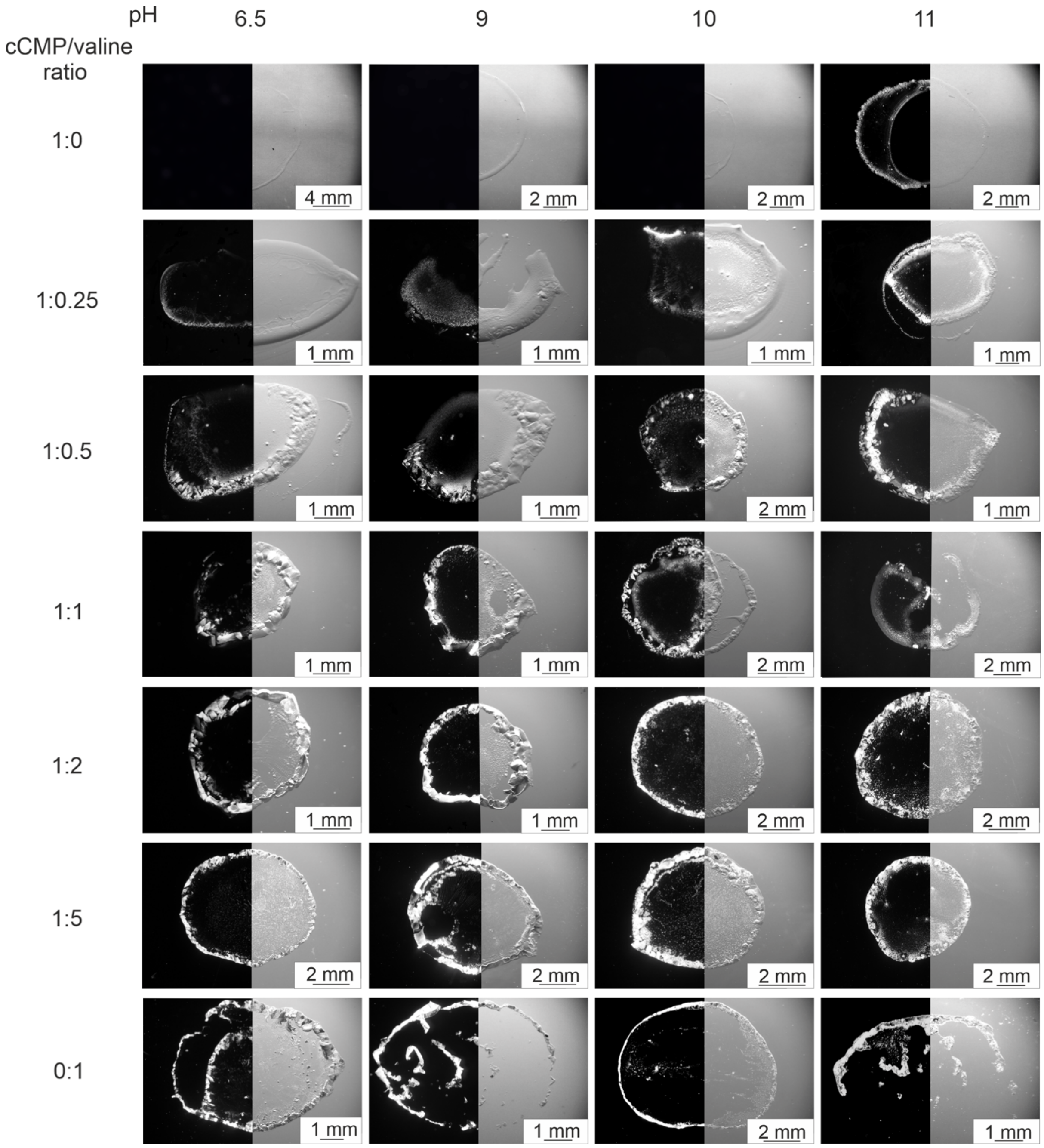

Figure S12. Morphological characterisation of cCMP and valine mixtures with varying ratios across different pH ranges by optical microscopy. The solutions containing cCMP and valine mixed in various ratios were prepared within a pH range 6.5-11 and subsequently incubated for 20 hours at room temperature. The left half of the images displays the internal structure of the samples viewed in cross-polarized light. In the experiment, 100 nmol of nucleotides and 25-500 nmol of valine were used. With increased pH and valine concentrations, a noticeable outer rim and the heterogeneity within the sample become more pronounced.

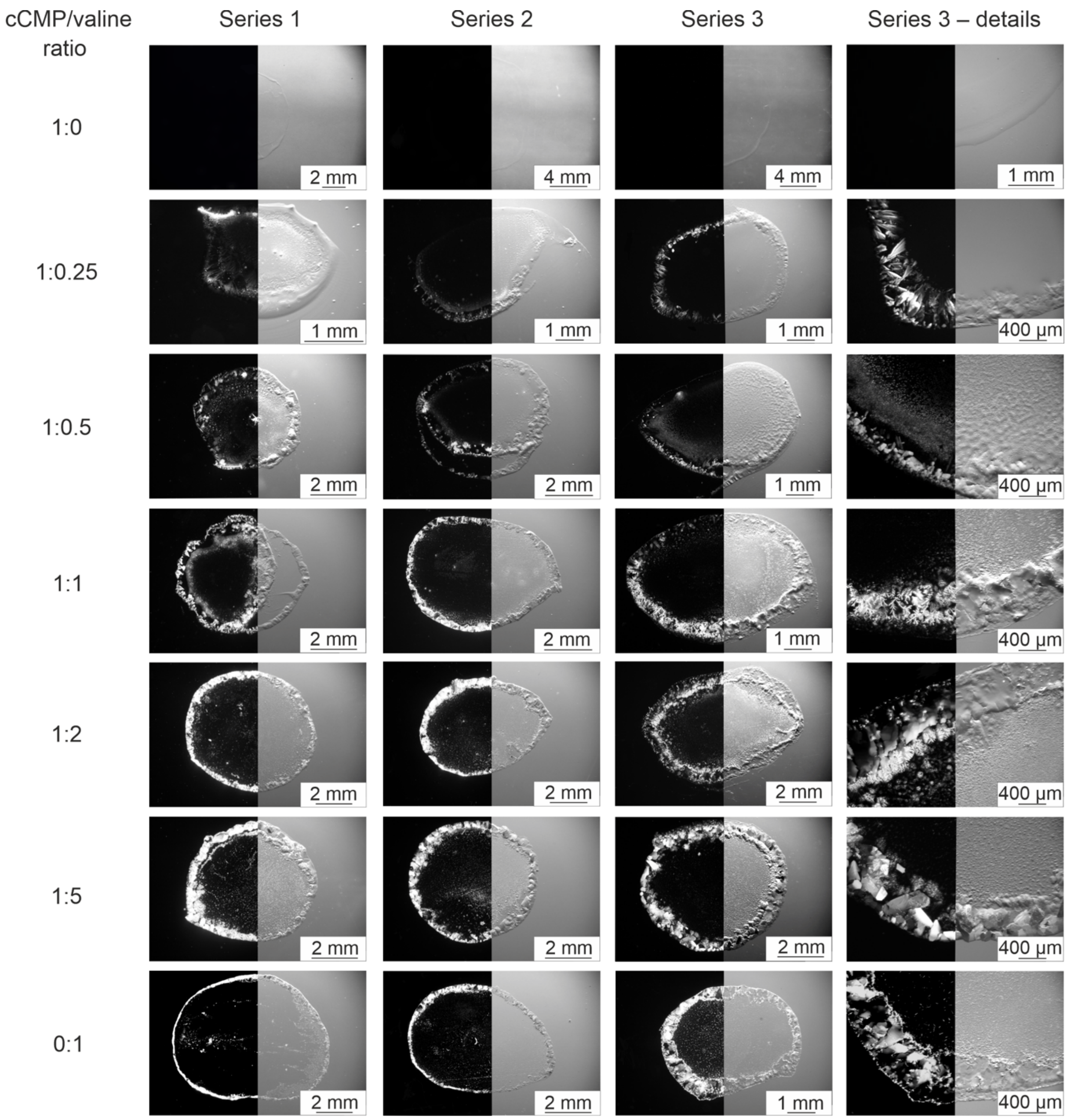

Figure S13. **Morphological characterisation of cCMP and valine mixtures with varying ratios at pH 10 by optical microscopy.** The solutions containing cCMP (100 nmol) and valine (25-500 nmol) were prepared at pH 10 and subjected to drying for 20 hours at room temperature. The left half of the images displays the internal structure of the samples viewed in cross-polarized light. Images were collected from three sets of experiments. Additionally, a detailed zoom-in image is also provided. As a result of the drying process, the distinct outer rim and the heterogeneity within the sample are clearly discernible, although the macroscopic appearance of the dried spot does not influence the oligomerisation chemistry (Fig. S6).

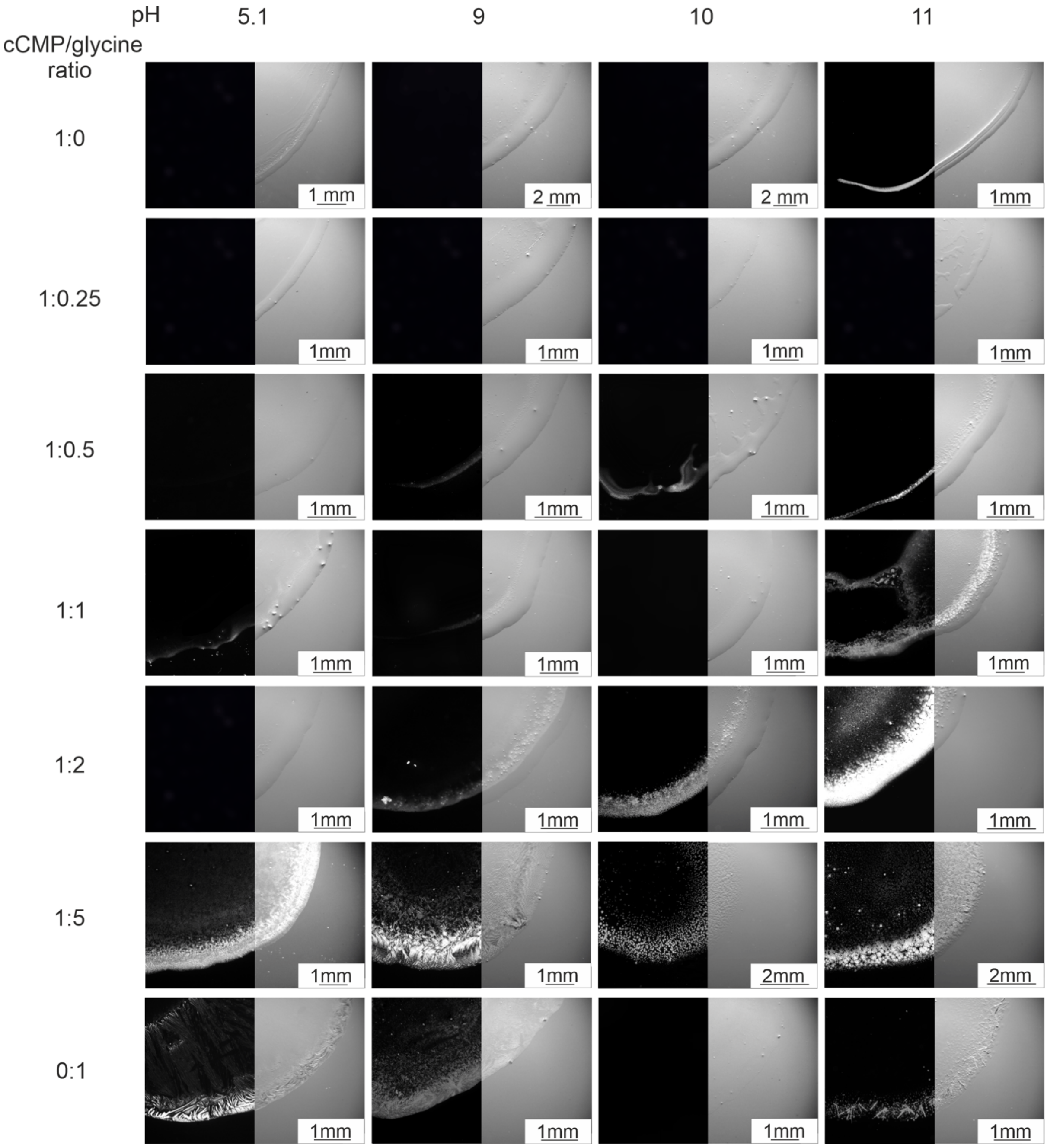

Figure S14. Morphological characterisation of cCMP and glycine mixtures with varying ratios across different pH ranges by optical microscopy. The solutions containing cCMP and glycine mixed in various ratios were prepared within a pH range 5.1-11 and subsequently incubated for 20 hours at room temperature. The left half of the images displays the internal structure of the samples viewed in cross-polarized light. In the experiment, 100 nmol of nucleotides and 25-500 nmol of valine were used.

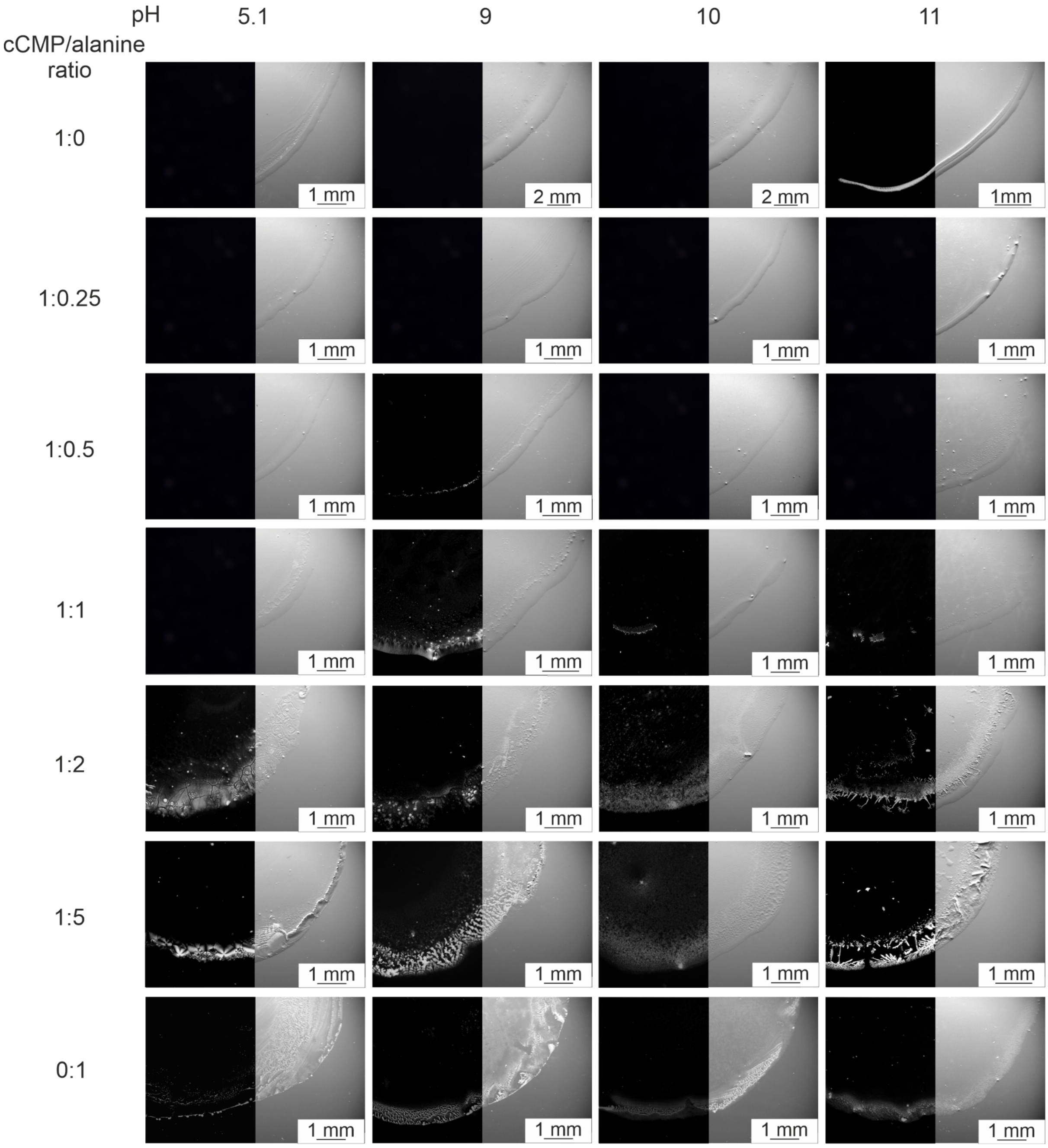

Figure S15. Morphological characterisation of cCMP and alanine mixtures with varying ratios across different pH ranges by optical microscopy. The solutions containing cCMP and alanine mixed in various ratios were prepared within a pH range 5.1-11 and subsequently incubated for 20 hours at room temperature. The left half of the images displays the internal structure of the samples viewed in cross-polarized light. In the experiment, 100 nmol of nucleotides and 25-500 nmol of valine were used.

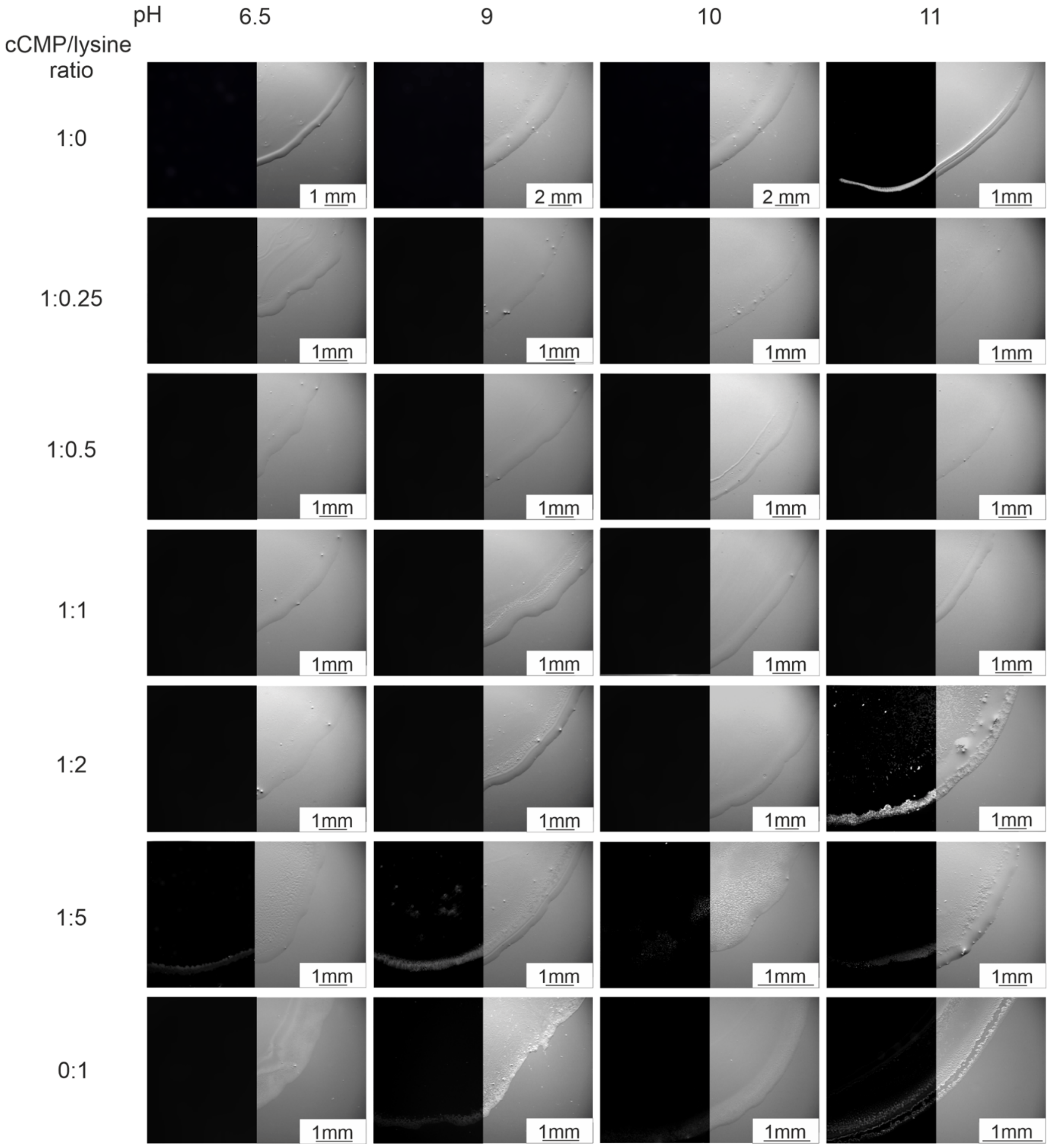

Figure S16. Morphological characterisation of cCMP and lysine mixtures with varying ratios across different pH ranges by optical microscopy. The solutions containing cCMP and lysine mixed in various ratios were prepared within a pH range 6.5-11 and subsequently incubated for 20 hours at room temperature. The left half of the images displays the internal structure of the samples viewed in cross-polarized light. In the experiment, 100 nmol of nucleotides and 25-500 nmol of valine were used.

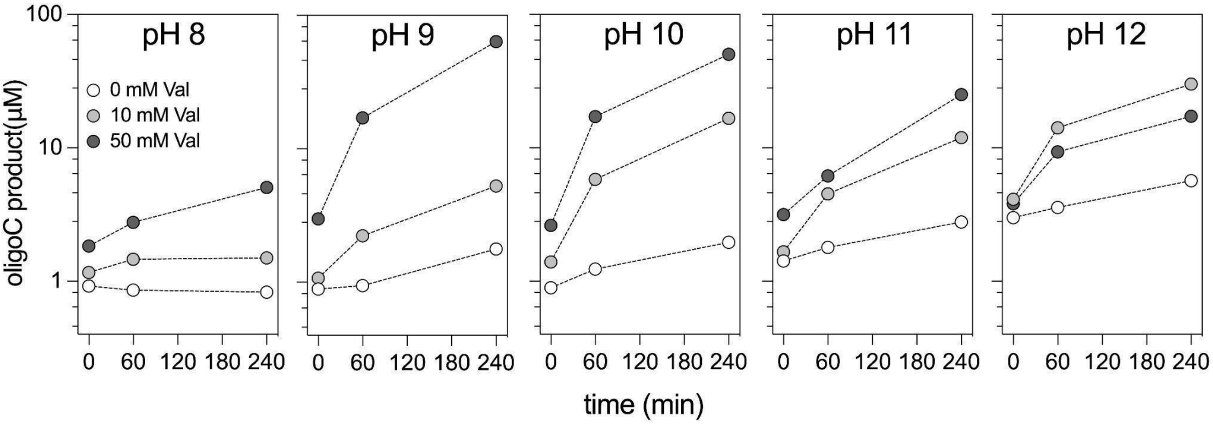

Figure S17. Kinetics of valine-assisted cyclic nucleotide oligomerisation at various pH ranges. Product evolution from cCMP oligomerisation is plotted (log-scale) in the absence and presence of valine across pH 8-12. All reactions were performed with 10 mM cNMP and 10-50 mM valine, with drying at room temperature (up to 4h). Quantitation was done on a reverse phase HPLC column coupled to ESI-TOF and by a custom-written LabVIEW program. The total product concentrations at different time points were used to calculate the average rate of the dry-state oligomerisation.

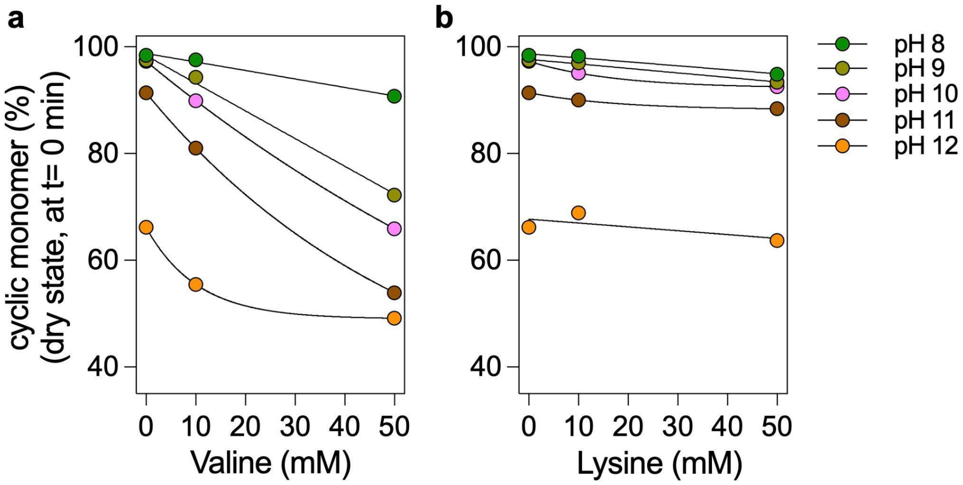

Figure S18. Effective cyclic nucleotide concentrations at the beginning of the dry state reactions. The percentage of active monomers with a cyclic phosphate end at t=0 minutes of the dry state is plotted for (**a**) valine and (**b**) lysine concentrations across different pH values (8–10). The solutions of cCMP (10 mM) and amino acids (10-50 mM) at various pH were dried at room temperature and promptly resuspended in nuclease-free water for MS analysis. The percentage was calculated by the ratio of cyc.P to (cyc.P+Lin.P) to determine the cyclic nucleotide hydrolysis. Higher pH and valine concentrations result in a reduction of the amount of cyclic nucleotides present at t=0 minutes in the dry state.

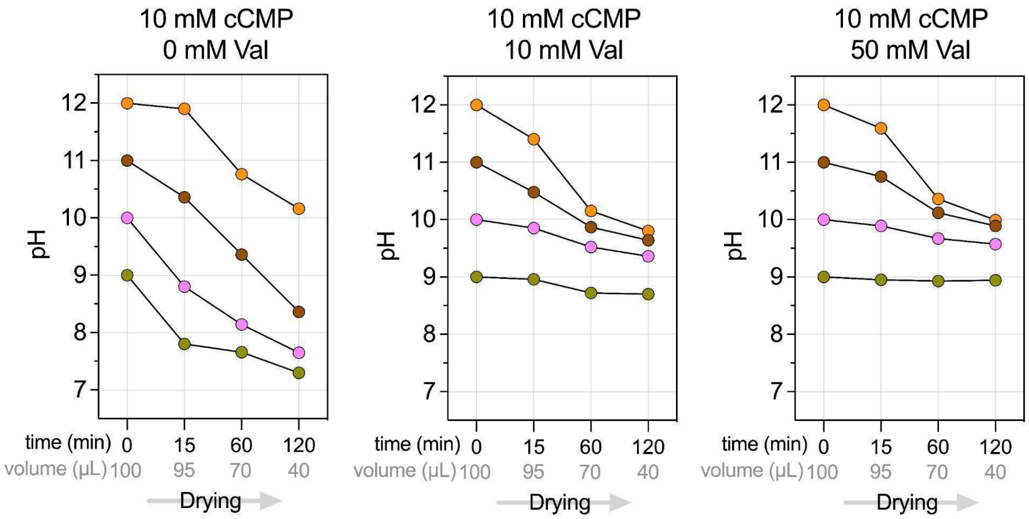

Figure S19. The pH of nucleotide and valine mixtures during the drying process. A 100 µL mixture of cCMP (10 mM) and valine (0-50 mM) at varying initial pH (9-12) was subjected to drying under airflow conditions at room temperature. The pH of the solution was measured at different drying times or volumes. The decrease in pH arises from air dissolution and the hydrolysis of cyclic monomers. While cCMP alone has no buffering effect due to the lack of an alkaline pK_a, valine resists pH changes in the nucleotide solution and tends to stabilise the pH closer to its amine pK_{aH}.

Detailed description of the results of quantum chemical calculations aimed to study the effect of bulk hydrophobicity/hydrophilicity on the reaction rates of amino acid catalysed transphosphorylation reactions

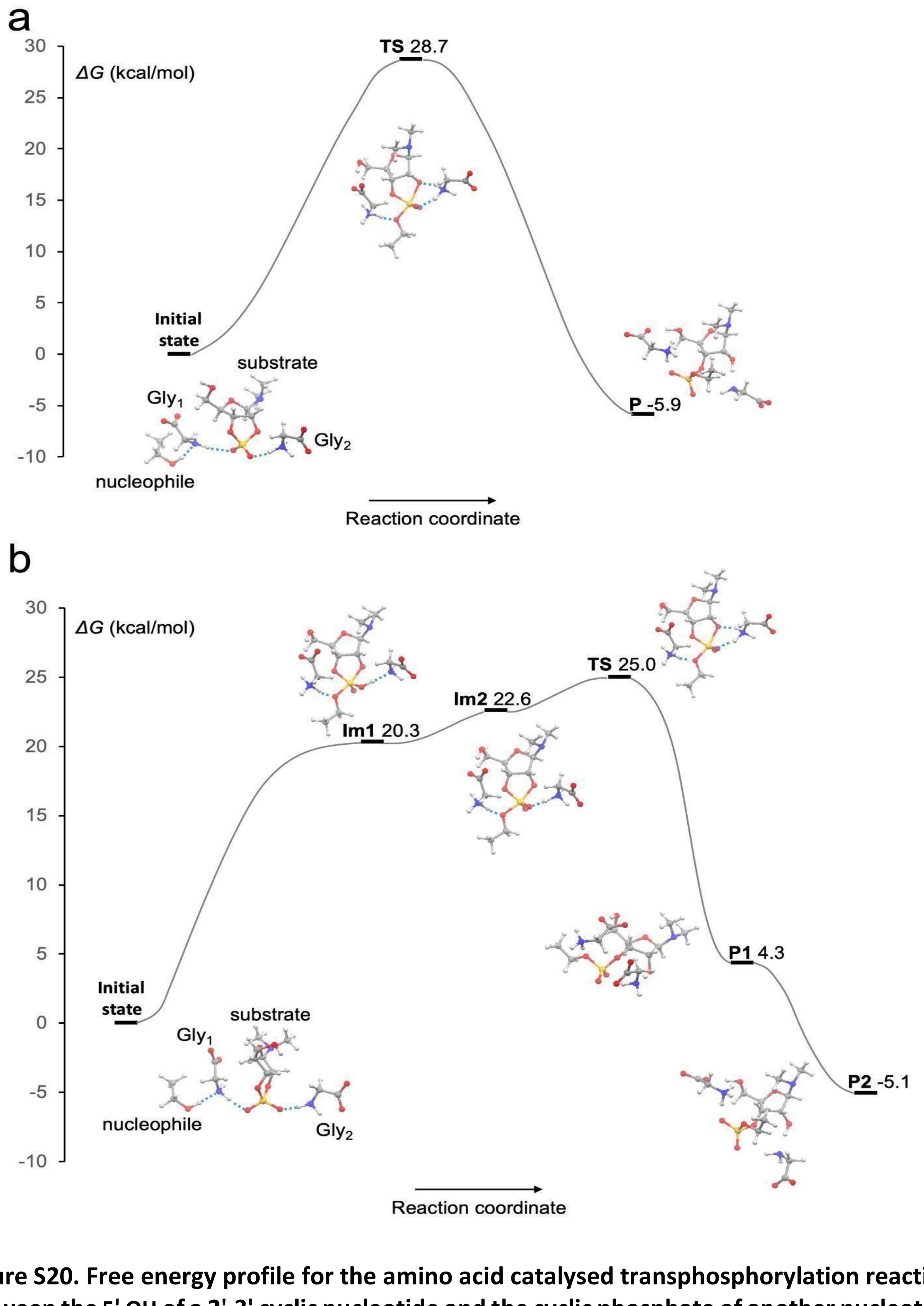

Figure S20. Free energy profile for the amino acid catalysed transphosphorylation reaction between the 5' OH of a 2',3' cyclic nucleotide and the cyclic phosphate of another nucleotide using a simplified model of the reaction complex. The ωB97XD/def2TZVPD free energy (ΔG) profile for the glycine-catalysed oligomerisation of cNMP was computed for **a**, hydrophobic

cyclohexane bulk medium (ε=2.0) and **b,** hydrophilic dimethyl sulfoxide bulk medium (ε= 46.8) using simplified models. The nucleophile is represented by an ethanol molecule, while β-1-dimethylamino-ribose-2',3'- cyclic phosphate stands for the substrate nucleotide. Computations were performed with the Gaussian09 computer code[12] and the PCM continuum solvation method[13]. For further technical details, see the Classical quantum chemical calculations part in the Materials and Methods section. *The free energy barriers do not represent absolute values for the reaction and should only be considered relatively within similar reaction schemes, as classical quantum chemical calculations convert electronic energies to free energies using molecular vibrational states but do not include statistical mechanics for the system's dynamic exploration of microstates.*

Our calculations indicate that in a **hydrophobic bulk medium** the reaction proceeds in a concerted manner, i.e. the two catalytic glycine molecules simultaneously mediate the proton transfer reactions involved in the mechanism: the glycine with an amino end deprotonates the nucleophile, while the protonated ammonium moiety of the zwitterionic glycine donates a proton to the leaving O2 group of the ribose. Overall, the reaction is exothermic (computed free energy change is -5.9 kcal/mol) and proceeds with an activation energy of 28.7 kcal/mol. For comparison, the computed activation energy of the uncatalysed reaction pathway is 54.0 kcal/mol.

The reaction pathway in a **hydrophilic bulk medium** is complex with multiple intermediates. The first intermediate (**Im1**) involves a proton transfer from the hydroxyl group of the nucleophile to the amino group of the anionic glycine molecule. This step is accompanied by the formation of a covalent bond between the phosphorus of the substrate nucleotide and the attacking oxygen of the nucleophile. However, due to the extremely flat potential energy surface near this intermediate state, locating the transition state geometry leading to **Im1** was technically infeasible. The second intermediate (**Im2**) is only 2.3 kcal/mol higher on the potential energy surface than **Im1** and differs from **Im1** only in the position of the proton shared between one of the phosphate oxygens and the amino end of the anionic glycine molecule. Again, due to the extremely flat potential energy hypersurface, identifying the transition state connecting **Im1** and **Im2** was impossible. The rate-determining step of the transphosphorylation reaction involves the cleavage of the O2-P bond in **Im2**. This step is initiated by a proton transfer from the zwitterionic glycine molecule (Gly2, see Figure S20) to the O2 oxygen of ribose, requiring an overall activation energy of 25.0 kcal/mol. In a hydrophilic bulk environment, the reaction is exothermic, with a free energy change of -5.1 kcal/mol.

Thus, the computations reveal that the hydrophobicity or hydrophilicity of the bulk medium might not influence the computed thermodynamics and activation free energies (28.7 and 25.0 kcal/mol for ε=2.0 and ε=46.8, respectively), suggesting no impact on the exponential part of the Arrhenius equation. The experimentally observed rate differences likely stem from the preexponential factor of the Arrhenius equation, which is proportional to the frequency of successful molecular collisions leading to chemical bond formation between reacting partners. This quantity was assessed by classical and quantum molecular dynamics simulations.

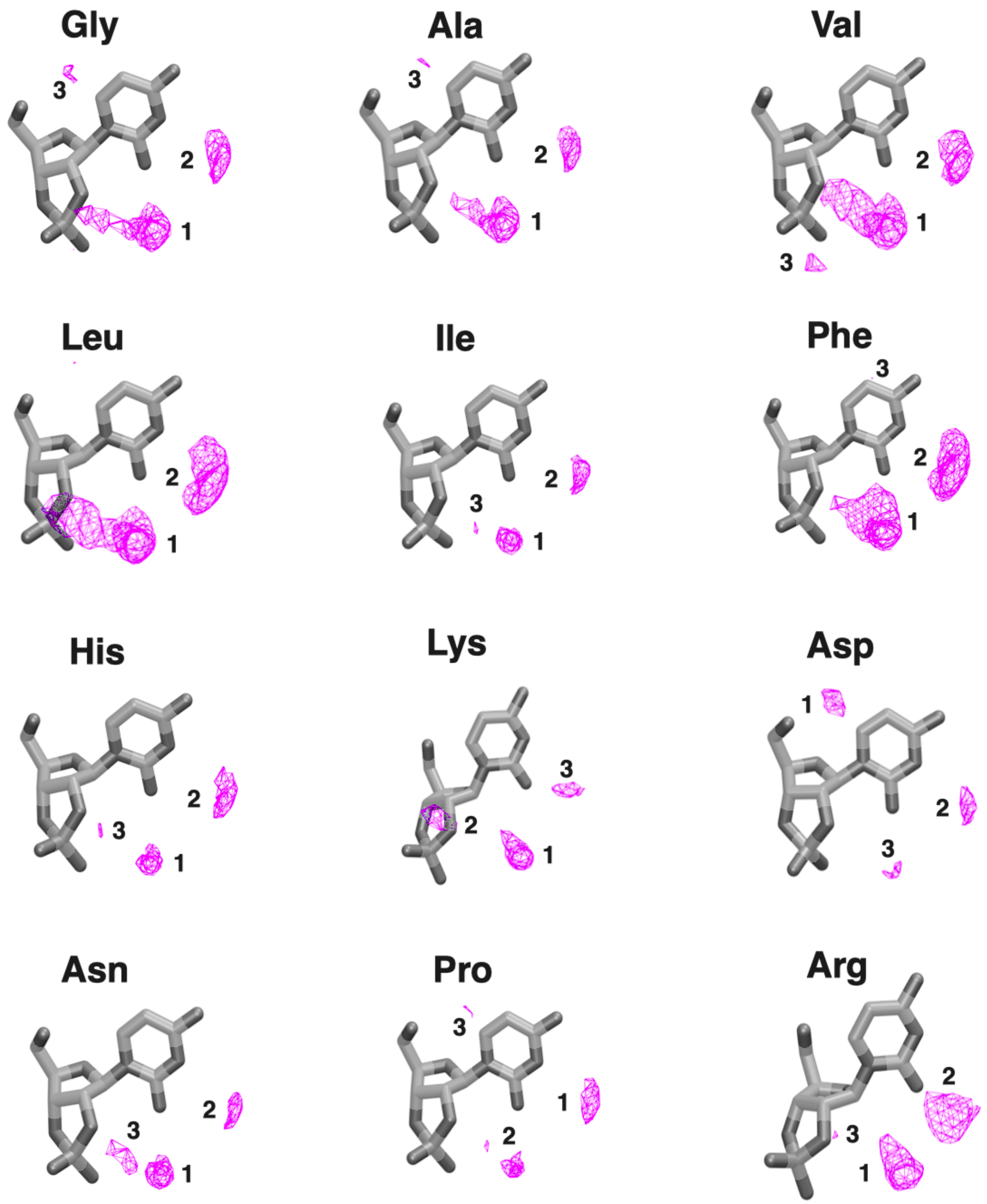

Figure S21. Distribution of the amino acids around the 2',3' cCMP nucleotide in molecular dynamics simulations. The simulations were performed using the AMBER22[21] program combined with the OL3[16] and ff14SB[18] force fields to describe the nucleotides and amino acids, respectively. For further technical details, see the Classical molecular dynamics simulations part (*vide infra*). The three highest-density regions (pink), indicating the most populated locations, are displayed for each amino acid. The density points are numbered from one to three, representing the frequency of occurrence in descending order.

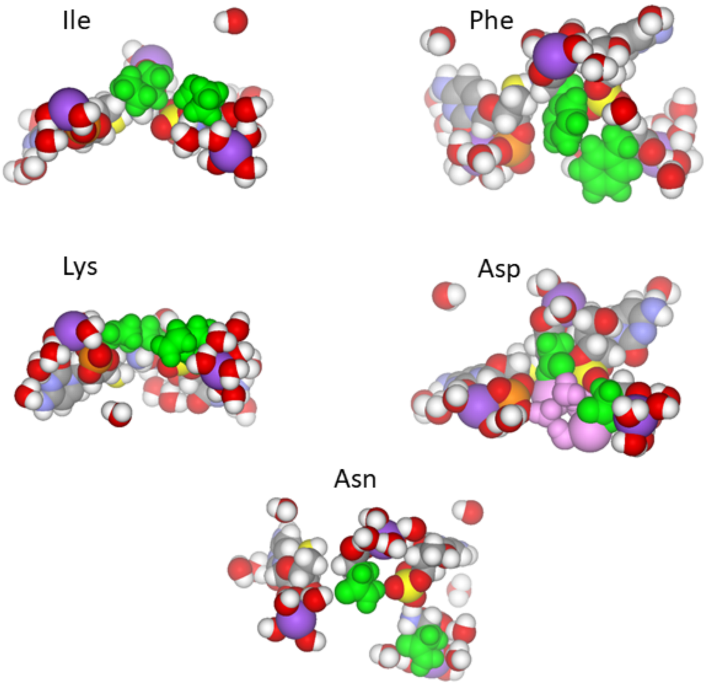

Figure S22. **Quantum molecular dynamics simulations of the reaction complexes formed by two 2',3'-cCMP anions and two amino acids.** The space-filling models show characteristic geometries adopted during a 2 ps long simulation. The size of the balls is proportional to the van-der-Waals radii of the atoms. The amino acid side chains are highlighted in green. The 5'O of the nucleophile and the phosphorus of the substrate are highlighted in yellow. Sodium cations and water molecules from their hydration sphere bound to the carboxylate moiety of Asp are highlighted in magenta. Color coding for the rest of the atoms: C-grey; N-blue; O-red; H-white; P-orange. Methodological details of the calculations are described in the Ab initio molecular dynamics part of the Materials and Methods sections (*vide infra*).

Reaction complexes containing amino acids with short hydrophobic side chains (like Val, Leu, Ile) do not undergo noticeable structural changes in the course of the simulations. In addition, the side chains do not significantly shrink the space available for the attack of the 5' OH group of the nucleophile at the substrate phosphate group. In contrast, the bulky side chains of Phe obstruct the space between the nucleophile and the phosphate group, preventing the attack. Besides its relatively low propensity to cluster near the phosphate of the nucleotide (see the classical molecular dynamics part in the main text), this obstruction might explain why Phe lacks catalytic activity in the transphosphorylation reaction. We note that the steric clash between the five-membered ring of proline and the phosphate of the substrate totally interferes with the definition of a reaction centre appropriate for the purpose of quantum molecular dynamics simulations.

While classical MD simulations predicted a similar binding preference for Lys as that of the catalytically most active amino acids (Val, Leu, Ile), its experimentally measured catalytic effect was noticeably weaker. Our *ab initio* molecular dynamics simulations indicated that the longer side chain of Lys does not significantly reduce the free space for the nucleophilic attack. However, the terminal NH_2 group of Lys is tightly linked to the 2',3' cyclic

phosphodiester linkage of the nucleotide serving as a nucleophile in the reaction, complicating the reorganisation of the reaction complex during the nucleophilic attack. This likely explains the decreased reactivity of Lys compared to Val, Leu, and Ile. Similarly, the side chain amino group of Asn establishes a very strong hydrogen bond with the phosphate group of the substrate, making it nearly impossible for the 5' OH group to attack the phosphate of the substrate.

The space inside the reaction complex available for the nucleophilic attack might also be shrunk by the presence of cations and their hydration shell. For example, the short anionic side chain of Asp constantly keeps at least one sodium cation coordinated in the vicinity of its carboxylate moiety and thus behaves as a relatively bulky group. This behaviour and the lowered preference of Asp to bind to the phosphate of the nucleotides might explain the very low catalytic activity observed for this amino acid in our experiments.

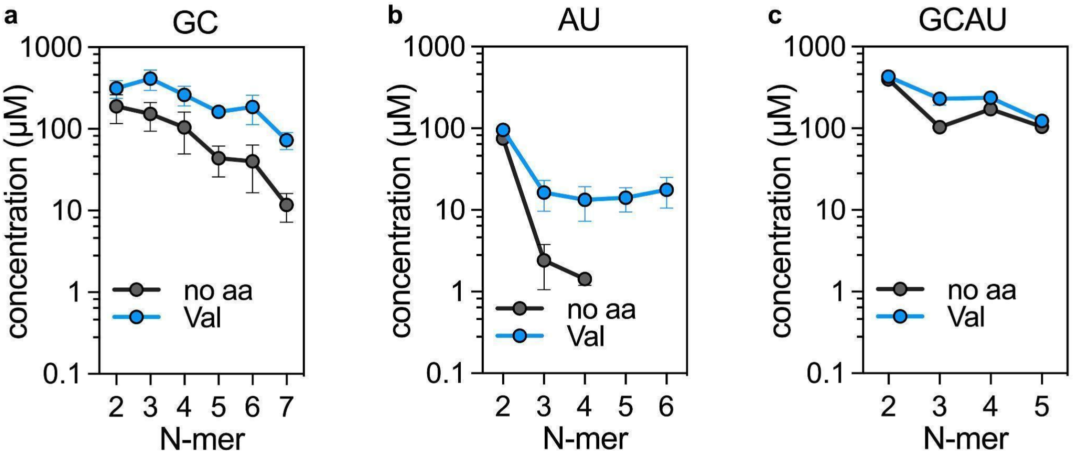

Figure S23. G/C, A/U, and G/C/A/U oligomers from 2',3'-cyclic nucleotides catalysed by valine. Concentrations of the oligomers of different lengths are plotted on a log scale for G/C/A/U oligomerisation in the absence and presence of valine. All reactions were performed with 40 mM cNMP and 100 mM valine at pH 10, incubated at room temperature for 20 hours. Quantitations were done on a reverse phase HPLC column coupled to ESI-TOF and by a custom-written LabVIEW program. Errors are given as S.D. of three independent experiments.

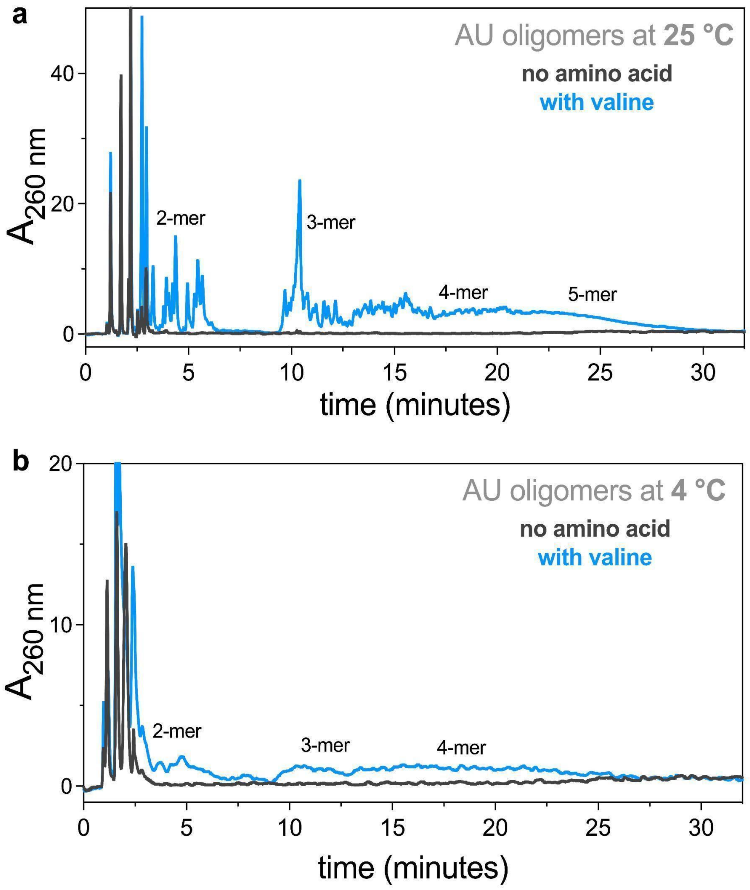

Figure S24. Analysis of the valine-catalysed A/U oligomerisation using HPLC. a, HPLC chromatograms at 260 nm illustrating AU oligomerisation at 25 °C. **b**, HPLC chromatograms of AU oligomerisation at 4 °C. In the experiments, 40 mM cyclic nucleotides (20 mM each of A and U) and 100 mM valine at pH 10 were rapidly dried and incubated for 20 h. Approximate elution times of oligomeric RNA with varying lengths and compositions are indicated on the plot. Quantitative estimations were conducted via ESI-TOF after ethanol precipitation of the oligomer products.

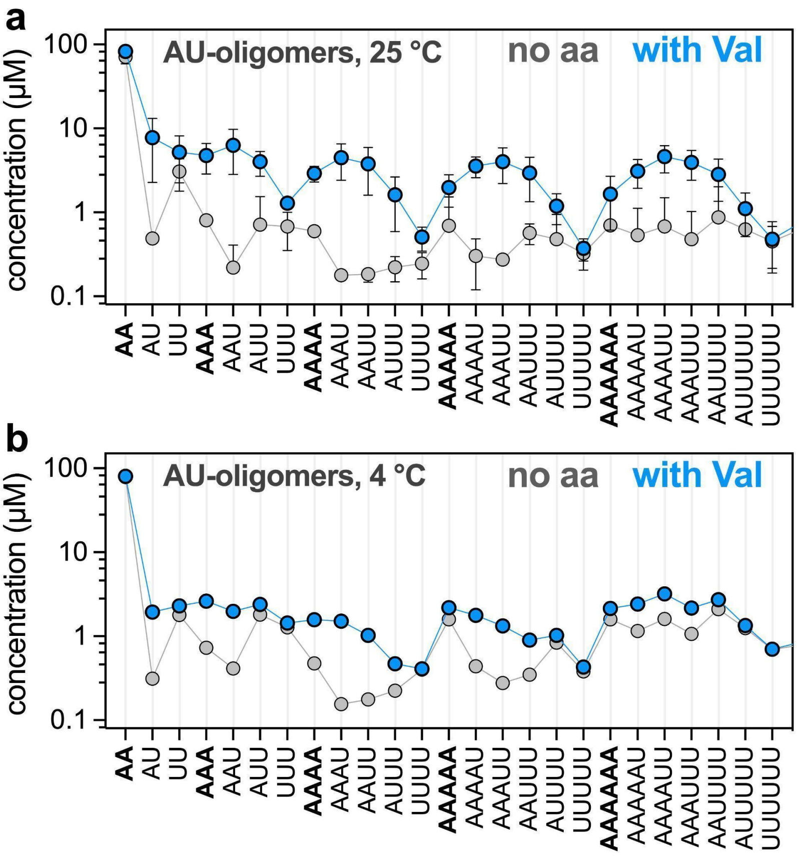

Figure S25. Valine-catalysed A/U oligomerisation. a, Concentrations of the AU-oligomers of different lengths and compositions from reactions conducted with and without valine at 25°C are plotted on a log scale. Errors are calculated as s.d. of results obtained from three independent experiments. **b,** Concentrations of the AU-oligomers of different lengths and compositions from reactions at 4°C. In the experiments, 40 mM cyclic nucleotides and 100 mM valine at pH 10 were rapidly dried and incubated for 20h. The resulting products underwent an ethanol precipitation step before the quantitation by HPLC-ESI-TOF.

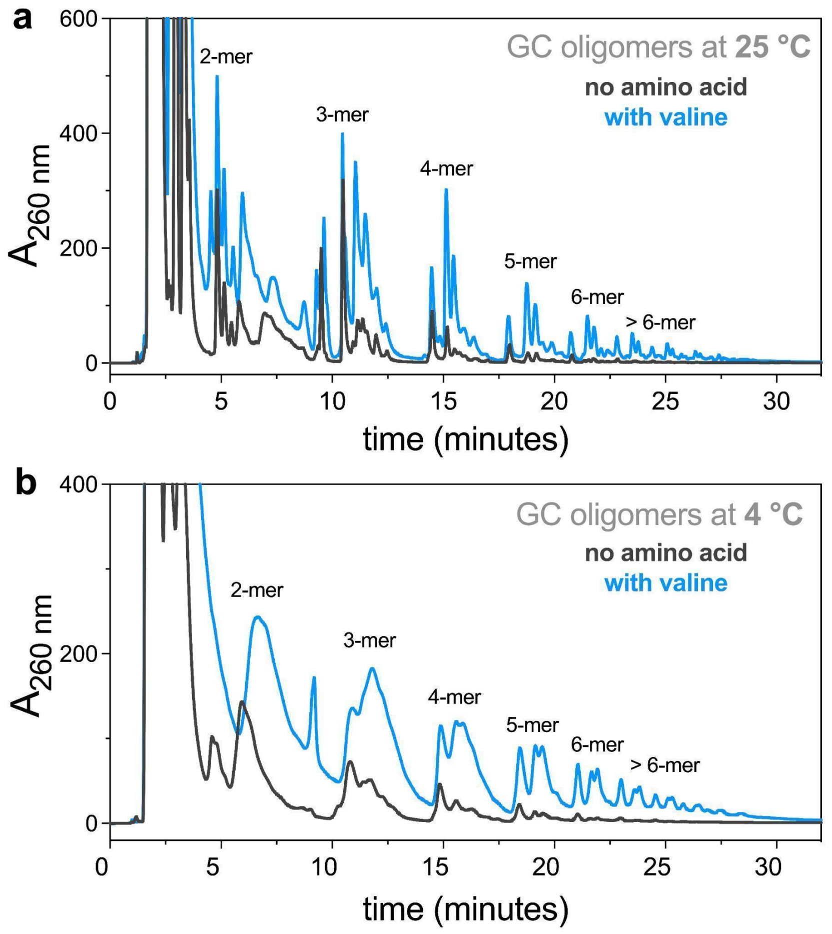

Figure S26. Analysis of the valine-catalysed G/C oligomerisation using HPLC. **a**, HPLC chromatograms at 260 nm illustrating GC oligomerisation at 25 °C. **b**, HPLC chromatograms of GC oligomerisation at 4 °C. In the experiments, 40 mM cyclic nucleotides (20 mM each of G and C) and 100 mM valine at pH 10 were rapidly dried and incubated for 20h. Approximate elution times of oligomeric RNA with varying lengths and compositions are indicated on the plot. Quantitative estimations were conducted via ESI-TOF after ethanol precipitation of the oligomer products.

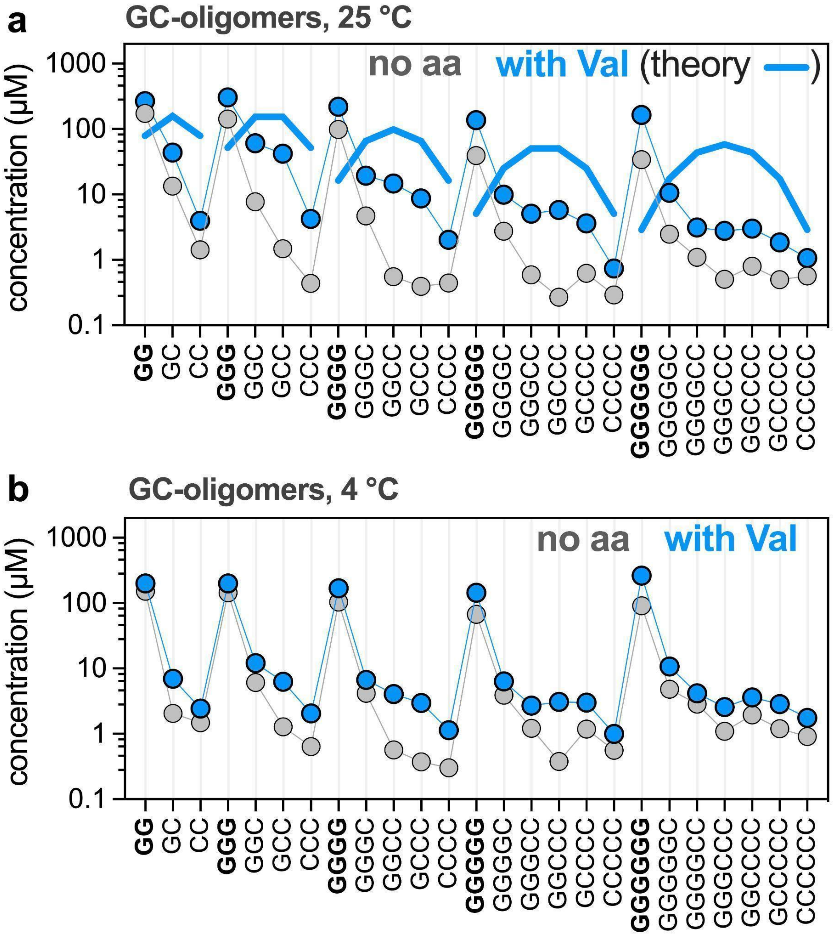

Figure S27. Valine-catalysed G/C oligomerisation. a, Concentrations of the GC-oligomers of different lengths and compositions from reactions conducted with and without valine at 25 °C are plotted on a log scale. Results represent the average from three independent experiments. The blue trace represents random oligomerisation in which G and C are equally efficient. **b,** Concentrations of the GC-oligomers of different lengths and compositions from reactions at 4°C. In the experiments, 40 mM cyclic nucleotides and 100 mM valine at pH 10 were rapidly dried and incubated for 20h. The resulting products underwent an ethanol precipitation step before the quantification by HPLC-ESI-TOF.

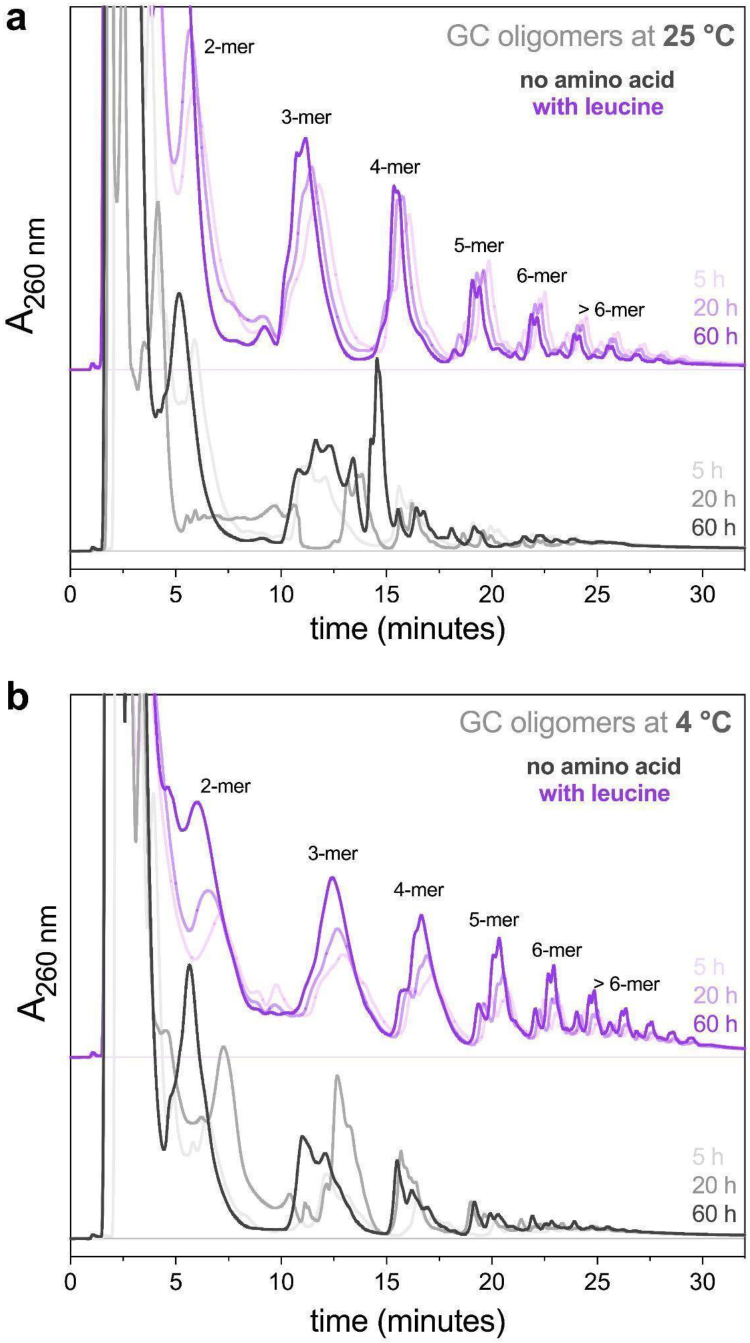

Figure S28. Analysis of the leucine-catalysed G/C oligomerisation using HPLC. a, HPLC chromatograms at 260 nm illustrating GC oligomerisation conducted with and without leucine at 25 °C. **b**, HPLC chromatograms of GC oligomerisation at 4 °C. In the experiments, 40 mM cyclic nucleotides (20 mM each of G and C) and 100 mM leucine at pH 10 were rapidly dried and incubated for 5h, 20h and 60h. Approximate elution times of oligomeric RNA with varying lengths and compositions are indicated on the plot. Quantitative estimations were conducted via ESI-TOF after ethanol precipitation of the oligomer products.

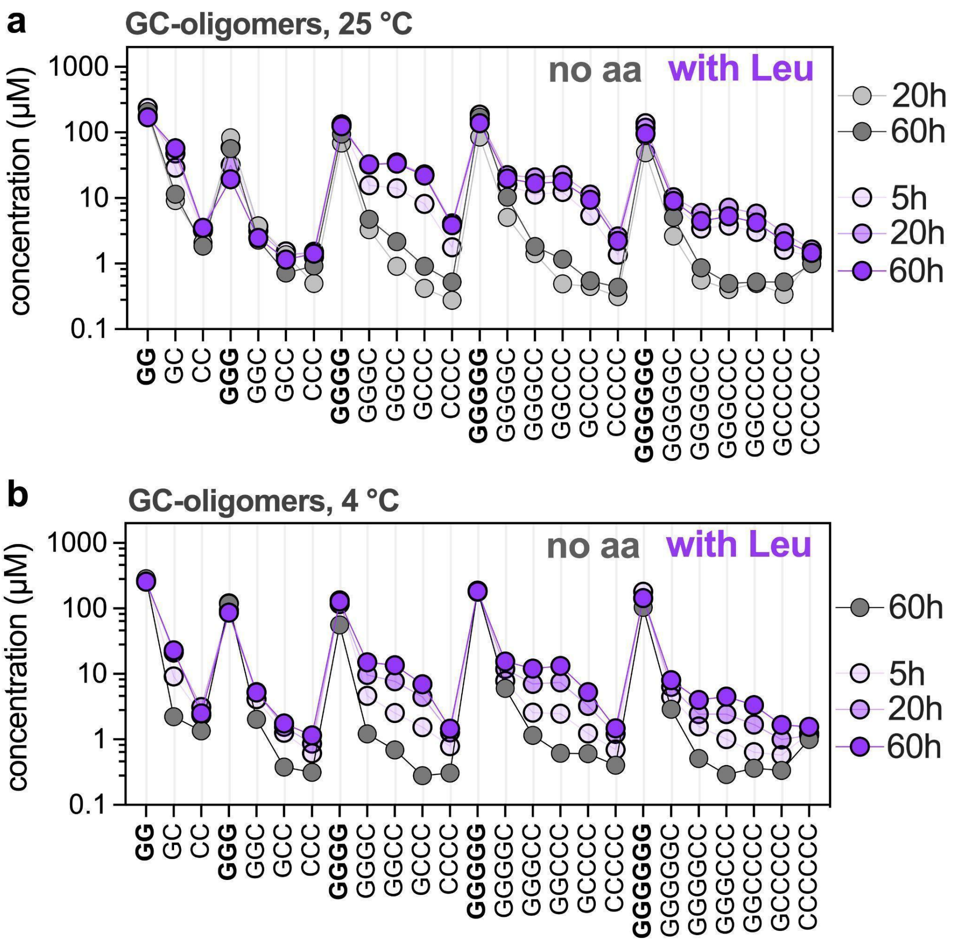

Figure S29. Leucine-catalysed G/C oligomerisation. a, Concentrations of the GC-oligomers of different lengths and compositions from reactions conducted with and without leucine at 25 °C are plotted on a log scale. **b,** Concentrations of the GC-oligomers of different lengths and compositions from reactions at 4°C. In the experiments, 40 mM cyclic nucleotides and 100 mM valine at pH 10 were rapidly dried and incubated for 5h, 20h and 60h. The resulting products underwent an ethanol precipitation step before the quantification by HPLC-ESI-TOF.

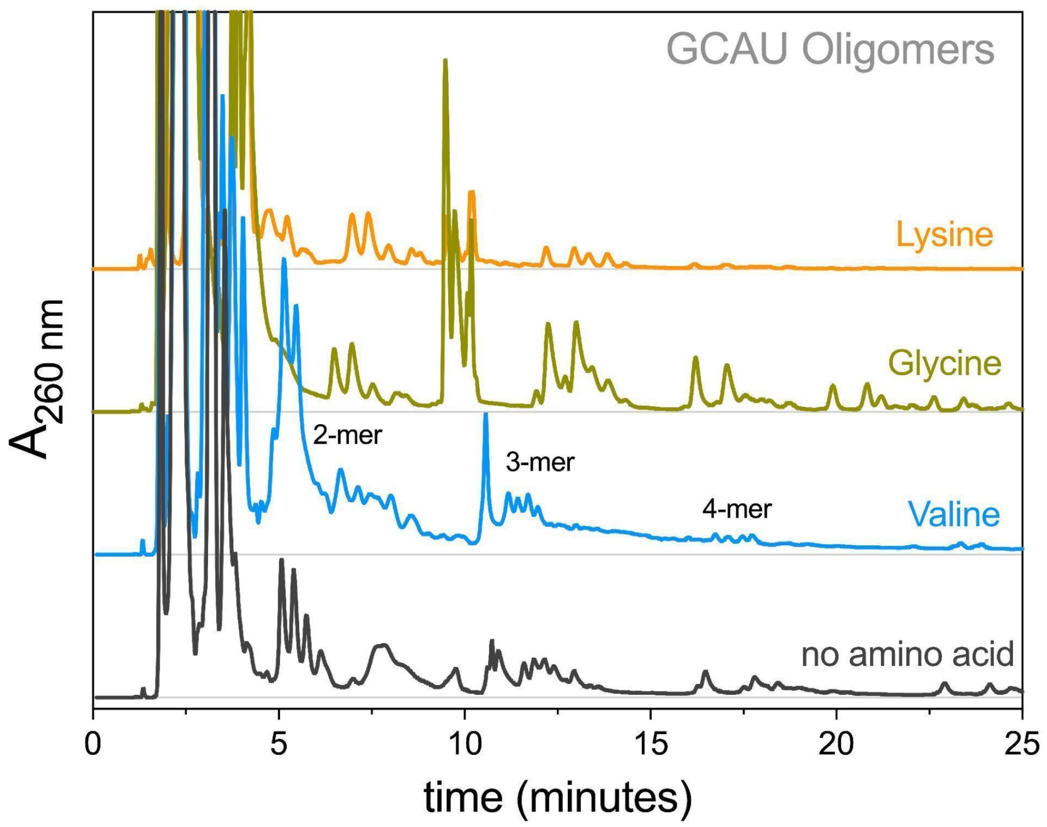

Figure S30. Analysis of the amino acid-catalysed G/C/A/U oligomerisation using HPLC. HPLC chromatograms at 260 nm illustrate GCAU oligomerisation conducted in the absence or presence of amino acids (valine, glycine and lysine) at room temperature. In the experiments, 40 mM cyclic nucleotides (10 mM each of G, C, A and U) and 100 mM valine at pH 10 were rapidly dried and incubated at 25 °C for 20h. Approximate elution times of oligomeric RNA with varying lengths and compositions are indicated on the plot. Quantitative estimations were conducted via ESI-TOF after ethanol precipitation of the oligomer products.

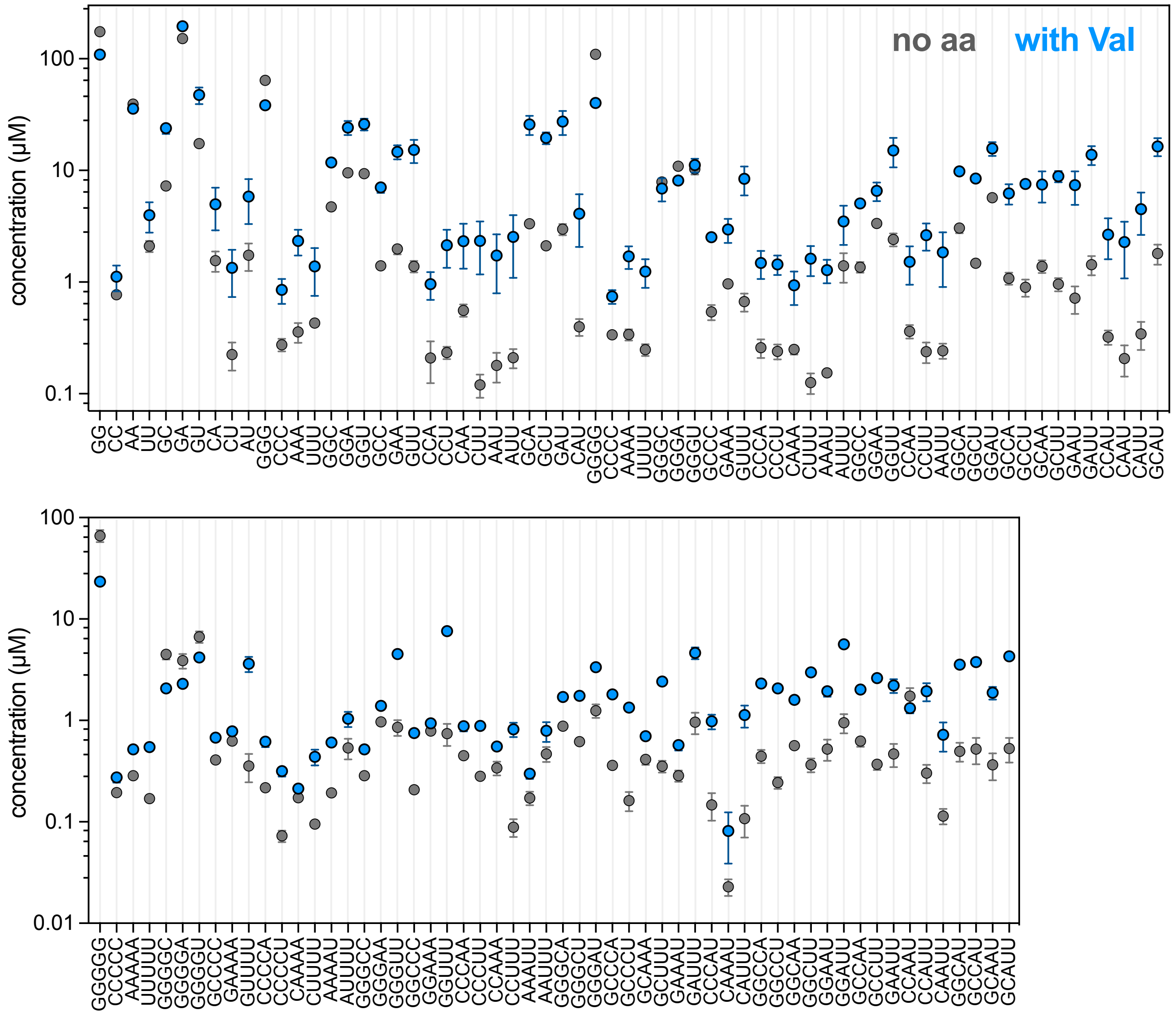

Figure S31. **Enhanced compositional diversity in the valine-catalysed co-oligomerisation of G/C/A/U nucleotides.** The concentrations of RNA oligomers (2-5 mer) are presented for various compositions for the GCAU reactions conducted with and without valine. In the experiments, 40 mM cyclic nucleotides (10 mM each of G, C, A and U) and 100 mM valine at pH 10 were rapidly dried and incubated for 20 h at 25 °C. The quantifications were done by LC-MS and a self-written LabVIEW program. The results are the mean with s.d. of three independent experiments.

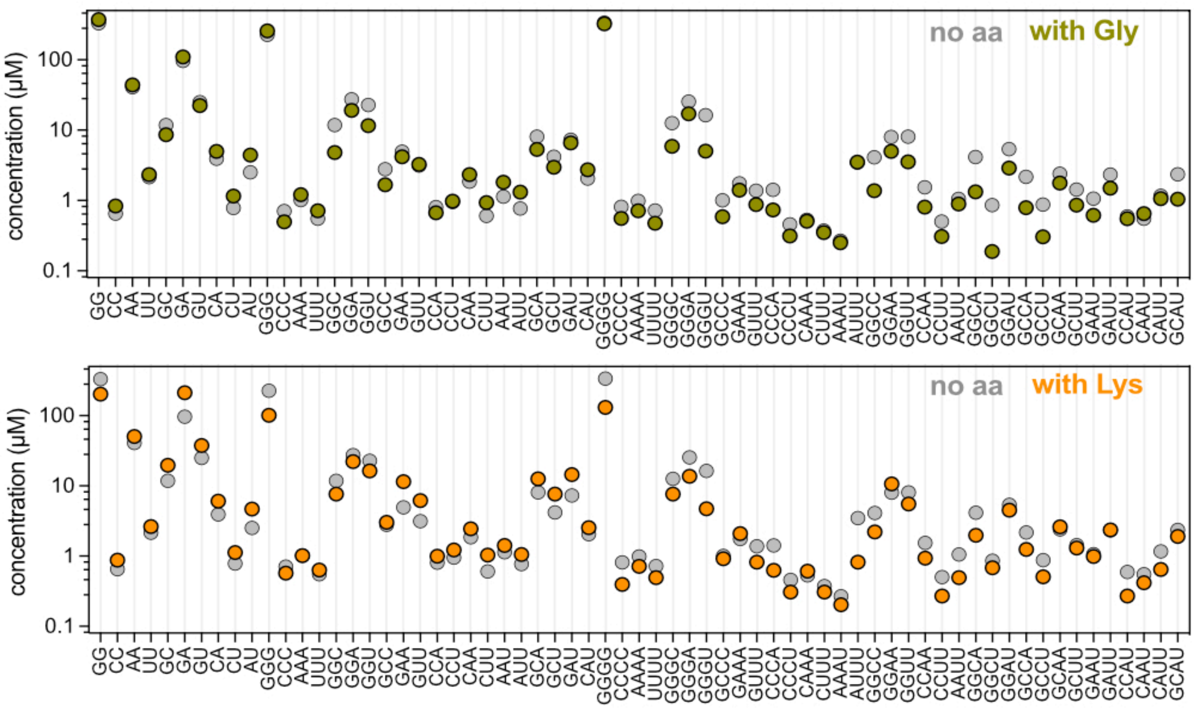

Figure S32. **Moderately enhanced compositional diversity in the glycine- and lysine-catalysed co-oligomerisation of G/C/A/U nucleotides.** The concentrations of RNA oligomers (2-5 mer) are presented for various compositions for the GCAU reactions conducted in the presence of glycine and lysine. In the experiments, 40 mM cyclic nucleotides (10 mM each of G, C, A and U) and 100 mM glycine or lysine at pH 10 were rapidly dried and incubated for 20 h at 25 °C. The quantifications were done by LC-MS and a self-written LabVIEW program. The results are the mean of two independent experiments.

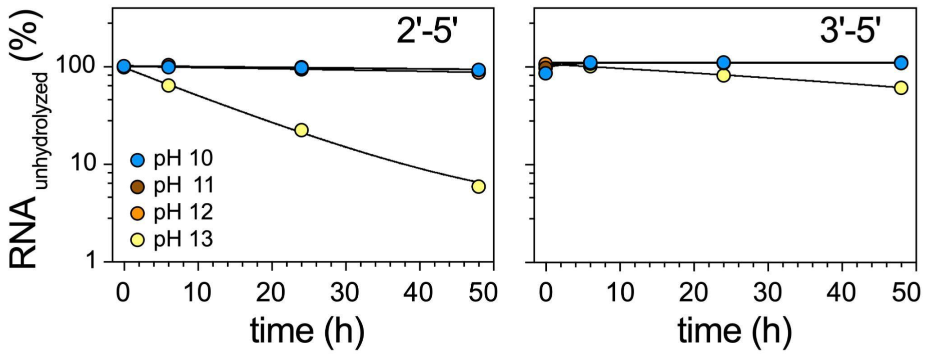

Figure S33. pH-dependent hydrolysis of oligoC at 25 °C investigated by HPLC. A 10 µM solution of custom-synthesized oligoC (3'-5': 18-mer and 2'-5': 15-mer) was incubated at pH 10-13 in 50 mM Arg as buffer for 48 h at room temperature. For HPLC analysis and to stop the hydrolysis reaction, the samples were diluted in 3x volume of 250 mM Tris at pH 6. The decrease in the oligoC peak area was quantitated, revealing that RNA with 2'-5' phosphodiester linkages degrades more quickly than RNA with 3'-5' linkages.

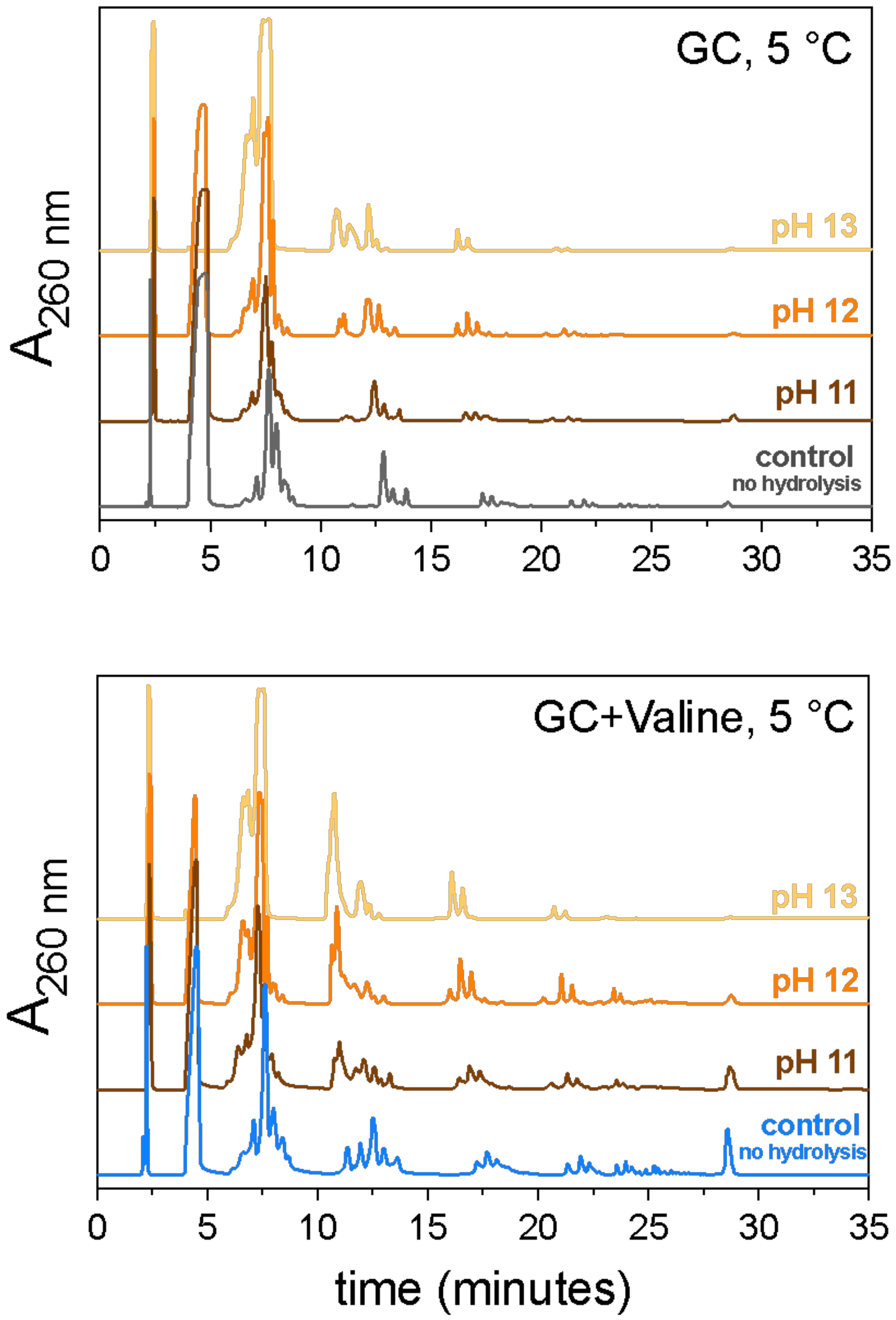

Figure S34. Hydrolysis of GC-oligomers at 5 °C investigated by HPLC. HPLC chromatograms at 260 nm illustrate the hydrolysis of GC oligomers incubated with 0.5M KCl at pH 11-13 and 5 °C for 24 h. The oligomers were synthesised at pH 10 using 40 mM cyclic nucleotides (20 mM each of G and C), with or without 100 mM valine at pH 10, after drying for 20 hours. The hydrolysis at higher pH is evident from the reduction of peak areas at higher elution times, corresponding to longer oligomers and an increase of peak areas at lower elution times, indicative of shorter oligomers and monomers. Further quantitative estimations were conducted by ESI-TOF.

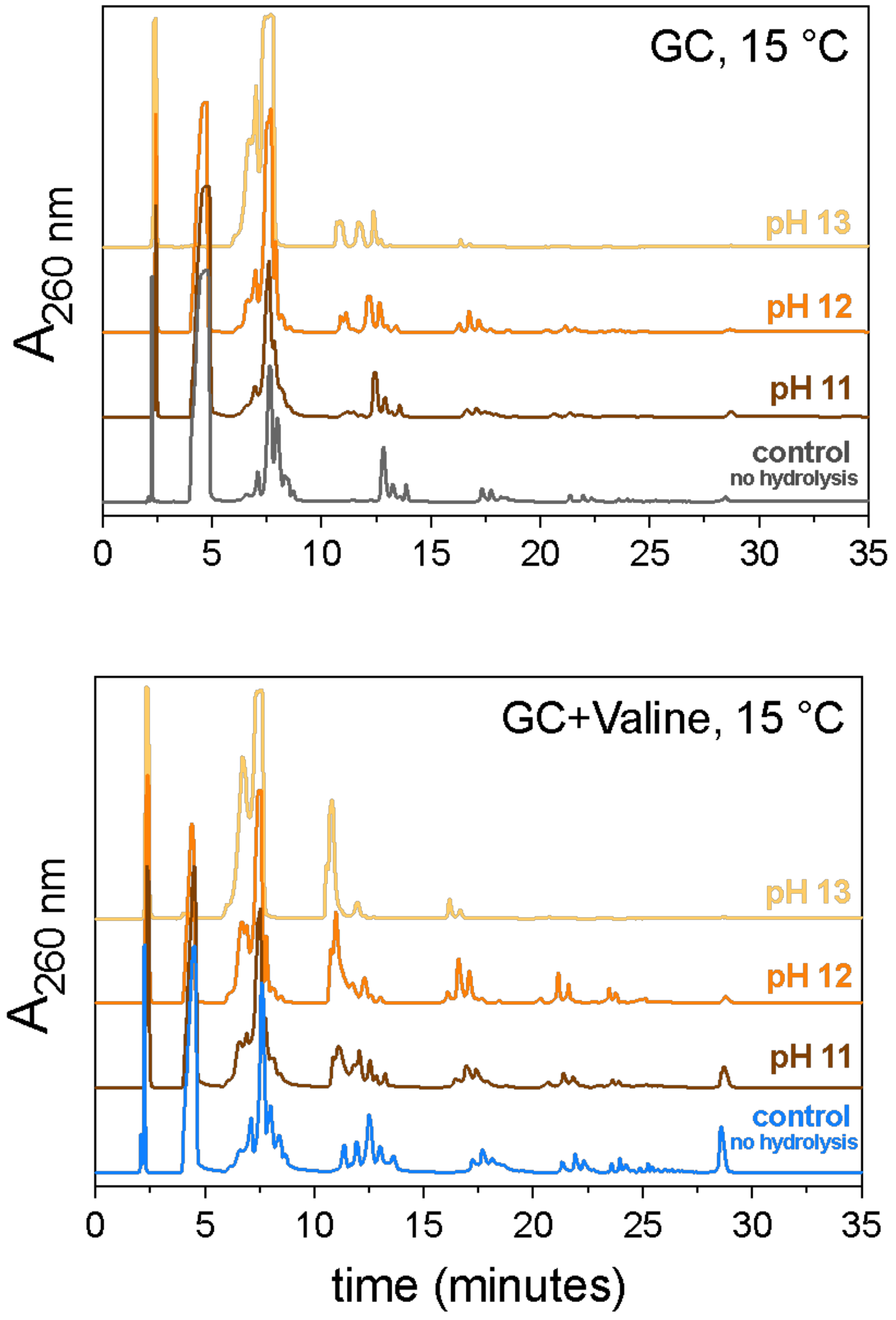

Figure S35. Hydrolysis of GC-oligomers at 15 °C investigated by HPLC. HPLC chromatograms at 260 nm illustrate the hydrolysis of GC oligomers incubated with 0.5M KCl at pH 11-13 and 15 °C for 24 h. The oligomers were synthesised at pH 10 using 40 mM cyclic nucleotides (20 mM each of G and C), with or without 100 mM valine at pH 10, after drying for 20 hours. The hydrolysis at higher pH is evident from the reduction of peak areas at higher elution times (for longer oligos) and an increase of peak areas at lower elution times (shorter oligos and monomers). Further quantitative estimations were conducted by ESI-TOF.

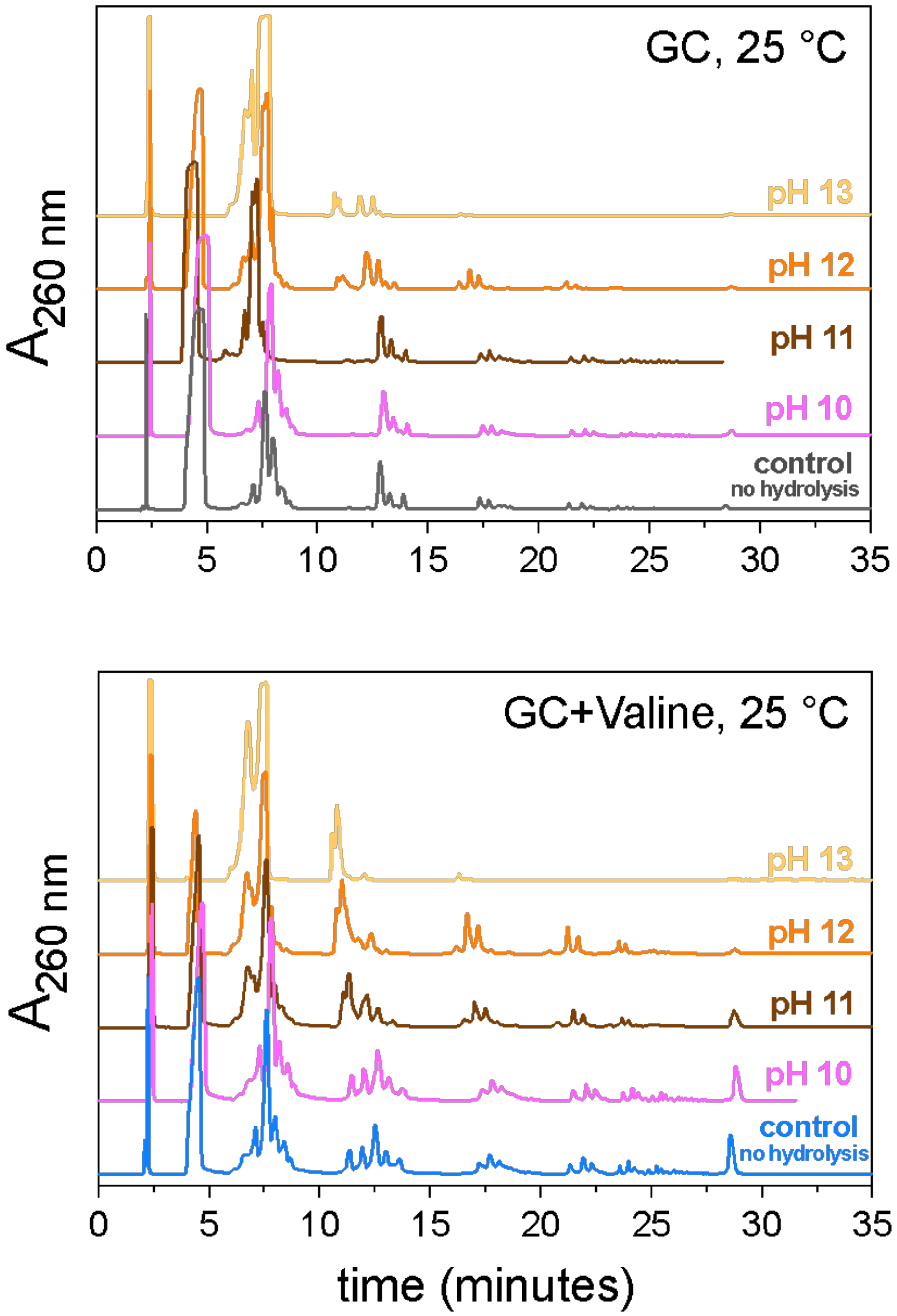

Figure S36. Hydrolysis of GC-oligomers at 25 °C investigated by HPLC. HPLC chromatograms at 260 nm illustrate the hydrolysis of GC oligomers incubated with 0.5M KCl at pH 10-13 and 25 °C for 24 h. The oligomers were synthesised at pH 10 using 40 mM cyclic nucleotides (20 mM each of G and C), with or without 100 mM valine at pH 10, after drying for 20 hours. The hydrolysis at higher pH is evident from the reduction of peak areas at higher elution times (for longer oligos) and an increase of peak areas at lower elution times (shorter oligos and monomers). Further quantitative estimations were conducted by ESI-TOF.

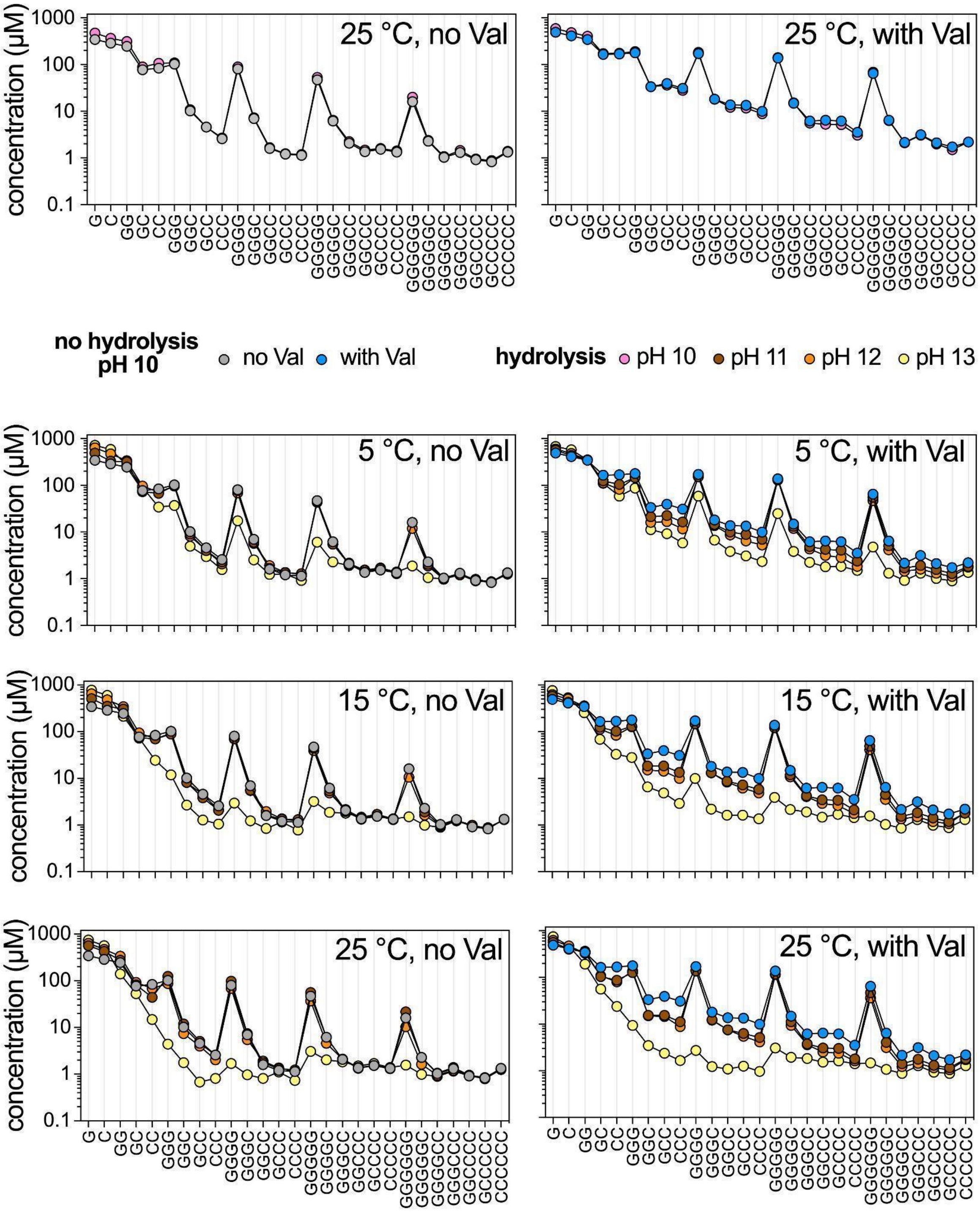

Figure S37. Temperature and pH-dependent hydrolysis of GC-oligomers. The concentrations of GC-oligomers in the absence (left panel) and presence (right panel) of valine are presented for various hydrolytic conditions (initial pH:10-13; temperature: 5 °C, 15 °C and 25 °C). The oligomers used for hydrolysis were synthesised at pH 10 using 40 mM cyclic nucleotides (20 mM each of G and C), with or without 100 mM valine at pH 10, after drying for 20 hours. These products were then incubated at different pH and temperature with 0.5M KCl for 24 h. The hydrolysis is evident from the reduction of longer oligo concentration and an increase of shorter oligo and monomer concentration at higher pH and temperature. All quantitative estimations were conducted by ESI-TOF.

It should be noted that at such a high pH, the RNA cleavage rate is expected to increase by a factor of 10 per unit of pH (see Ref. 40 in the main manuscript, Li, Y. and Breaker, R. R., JACS, 121, 5364–5372 (1999)). Our results of RNA not degrading significantly at very high pH are actually in accordance with the estimates provided in the Breaker study, suggesting that hydrolysis is not a dominant factor under the conditions used in our experiments. For example, extrapolating from the Breaker paper, we expect at pH 12 and 5 °C, a halftime of an 8mer of around 200h in liquid water. This calculation is based on the formula (e) from Li, Y. & Breaker, R. R., Kinetics of RNA Degradation by Specific Base Catalysis of Transesterification Involving the 2'-Hydroxyl Group. *J. Am. Chem. Soc.* 121, 5364–5372, (1999), and the equation below is reprinted with permission. (Copyright 1999 American Chemical Society)

$$k_{\text{projected}} = k_{\text{background}} \times 10^{\{0.983(\text{pH}-6)\}} \times 10^{\{-0.24(3.16-[\text{K}^+])\}} \times 69.3[\text{Mg}^{2+}]^{0.80} \times 3.57[\text{K}^+]^{-0.419} \times 10^{\{0.07(T_1-23)\}}$$

given in units of 1/min and per base linkage with a background rate of 1.3e-9/min. It can also be rewritten in Excel syntax with concentrations given in M and Temperature in °C:

$k_{\text{projected}}$=1.30E-09*10^(0.983*(pH-6))*10^(-0.24*(3.16-[K+]))*69.3*([Mg2+]^0.8)
3.57([K+]^-0.419)*10^(0.07*(T1-23))

Also, it should be noted that the dry-state reaction conditions may further mitigate the RNA hydrolysis.

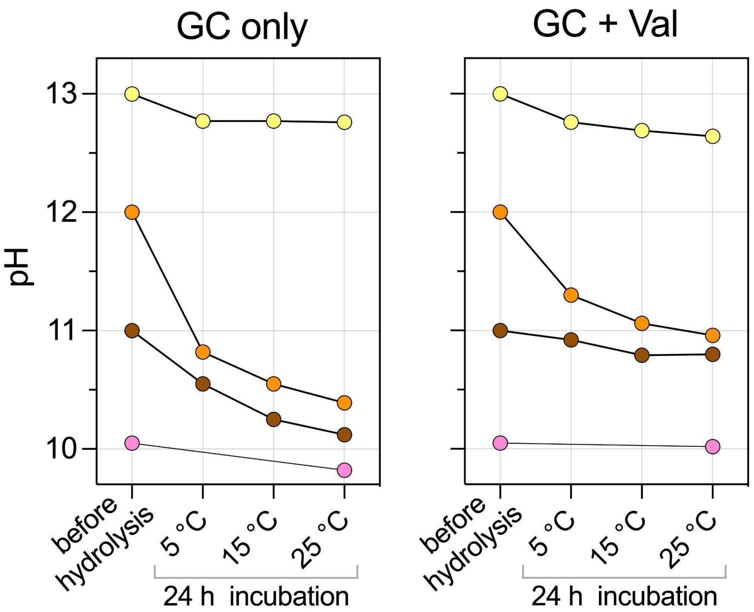

Figure S38. pH-drop as a result of hydrolysis. The pH of every hydrolytic condition explored in Figure S37 was measured for GC-oligomers, both in the absence (left) and presence (right) of valine, after a 24-hour incubation. Compared to GC-oligos without valine, valine appears to have a buffering effect and resists the pH shift more.

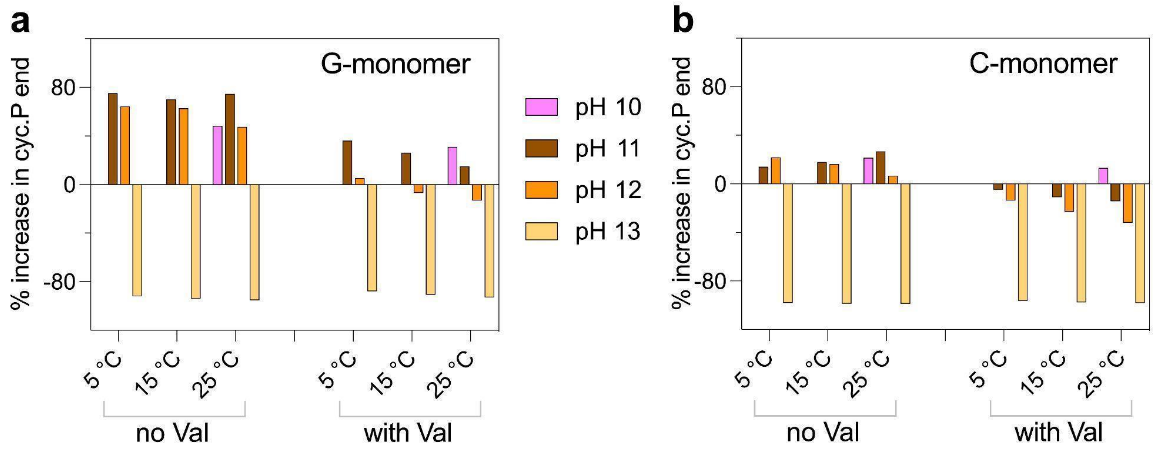

Figure S39. Increased nucleotide monomers with 2', 3'-cyclic phosphate (cNMP) as a result of hydrolysis. a, The percentage increase in concentrations of cGMP in GC-oligomer mixtures with and without valine is plotted for all hydrolytic conditions investigated in Figure S37. **b,** The percentage increase in concentrations of cCMP is plotted, similar to **a**. All quantitative estimations were conducted by ESI-TOF.

Table S1. The average number of amino acid molecules interacting with the cyclic phosphate moiety, O5' or nucleobase parts of 2',3' cCMP, as determined by classical molecular dynamics simulations. The quantities were derived by assessing the number of hydrogen bonds formed between the amino groups of the amino acids and the acceptor sites on the cyclic nucleotide. Hydrogen bond interactions were identified based on the N…O and N…N distances between the donor and acceptor atoms, with a distance cutoff of 3.5 Å.[a]

Amino acid	Phosphate	O5' group	Nucleobase
Gly	1.139	0.143	0.841
Ala	1.163	0.149	0.937
Val	1.364	0.113	1.120
Leu	1.140	0.123	1.036
Ile	1.182	0.110	1.100
Phe	0.676	0.085	1.001
Lys	1.648	0.094	0.804
Arg	0.607	0.089	0.467
His	0.617	0.092	0.731
Asp	0.329	0.095	0.471
Asn	1.123	0.094	0.818
Pro	1.041	0.120	0.974

[a]A population over 1 indicates two or more amino acids simultaneously interacting with the group on average.

Materials and methods

Chemicals

Ribonucleoside 2',3'-cyclic phosphates (including cAMP, cUMP, cGMP from Biolog Life Science Institute and cCMP from Sigma Aldrich), amino acids (from Sigma Aldrich), glycogen, and ammonium acetate (from Sigma Aldrich), as well as KOH, HCl, and ethanol (from Carl Roth), were used in the experiment. Polymerisation reactions were conducted using either glass slides with cavities (from Carl Roth) or in 24-well plates (from Greiner CELLSTAR®)

Dry-state polymerisation of ribonucleoside 2',3'-cyclic phosphates

Stock solutions of amino acids and nucleotides (100-200 mM) were prepared in nuclease-free water (Invitrogen™). The reaction mix was prepared by mixing the corresponding stock solutions to attain the required concentration and pH (adjusted with KOH or HCl).

For a typical polymerisation reaction, small volumes of the reaction mix (10µL in Figures 1 and 2, 50 µL in Figure 3) were placed on the glass slide or multi-well plate and were allowed to dry under airflow (ca 0.5-2h). The dried samples were then incubated at room temperature for 20 hours. After the incubation, the samples were extracted at least twice the volume with nuclease-free water. In some experiments, the samples were further purified by ethanol precipitation to enrich the oligonucleotides, as described in the following section.

For morphological characterisation of the dried nucleotide and amino acids mixtures, microscopic images were acquired using an Olympus SZX10 stereomicroscope equipped with an Olympus DF PLAPO 1.25X objective and a Canon EOS 2000D camera.

Ethanol precipitation

The extracted samples were mixed with glycogen (20 µg) and ammonium acetate (at a final concentration of 500 mM). To this, 2-3x volume cold 100% ethanol was added. After an overnight incubation at 4 °C, the sample was centrifuged at 4 °C for 30 minutes at 21000g. The supernatant was discarded, and the pellet was washed with 50 µl of cold 70% ethanol by mixing and centrifuging again at 4 °C for 30 minutes. The supernatant was removed, and the washed pellet was dissolved in nuclease-free water for the LC-MS analysis.

HPLC ESI-TOF analysis

Oligomer quantification was performed using high-performance liquid chromatography (Agilent 1260 Infinity II) coupled with an electrospray ionisation time-of-flight mass spectrometer (Agilent 6230B with Dual AJS ESI). To ensure accuracy and determine the timing of the HPLC, pre-formed oligomers (polyG and polyC) ranging from lengths 2-10 with 3' phosphate (from Biomers) were used as standards. Reverse-phase ion-pairing HPLC was employed to separate oligomers of different lengths on an Agilent AdvanceBio Oligonucleotide C18 Column (4.6 x 150 mm, 2.7 µm, heated to 60 °C), with a gradient elution at a flow rate of 1 mL/min. The eluents consisted of water (Bottle A) and 50:50 methanol-water (Bottle B) containing 8 mM triethylamine (TEA) and 200 mM hexafluoroisopropanol (HFIP). The separation process began with a 5-minute flush with 1% B, followed by a gradual

increase to 30% B over 22.5 minutes and then to 40% B over 15 minutes. Subsequently, the column was flushed with 100% B for 5 minutes before re-equilibration at 1% B for 6 minutes. Detection of eluted oligonucleotides was achieved using ESI-TOF in negative mode, employing specific source parameters: Gas temperature: 325 °C, Drying gas flow: 13 l/min, Sheath gas temperature: 400 °C, Sheath gas flow: 12 l/min, VCap: 3500V, Nozzle Voltage: 2000V.

MS data analysis by custom-written LabVIEW program

The MS data acquired from HPLC ESI-TOF was converted to .mz5 format using MSConvert, a component of ProteoWizard[1]. Subsequently, it was imported into a custom LabVIEW program (Spectral_browser_3.58) for detailed analysis (see Figure S40 below).

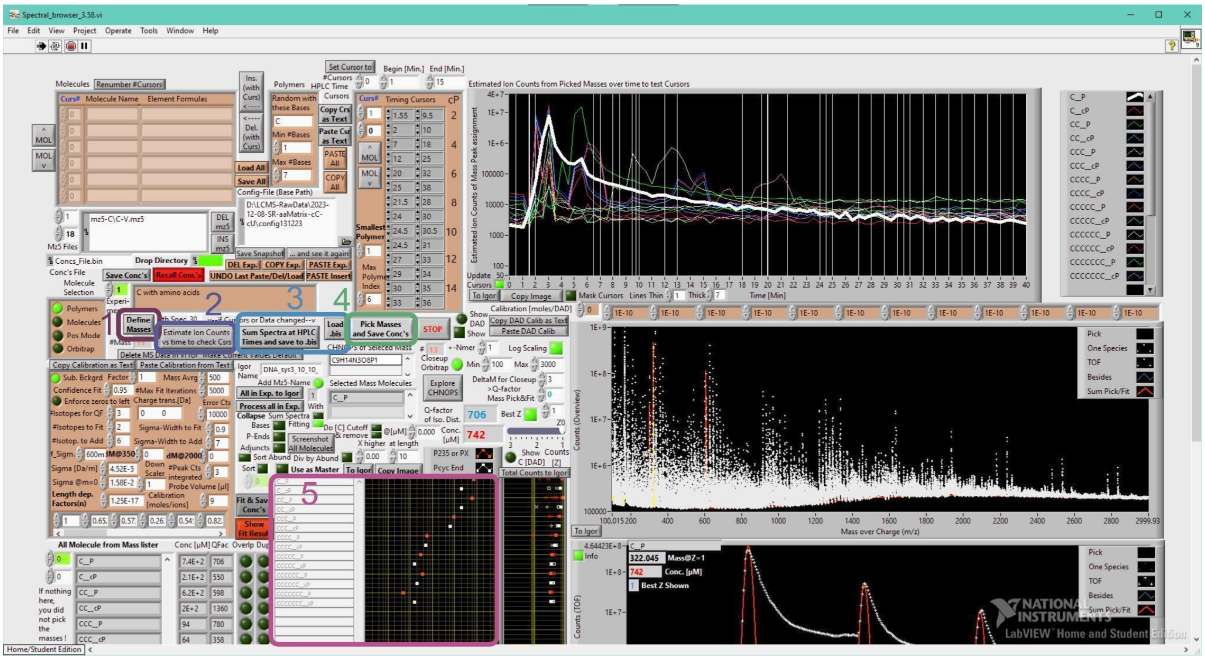

Figure S40. User interface of SpectralBrowser v3.58. The MS data analysis pipeline involves a systematic process denoted by numbers (1-5); each step is colour-coded and described below.

1. Mass List Generation

2. Data Loading and Mass Chromatogram Plotting

3. Summation of Raw Mass Spectra

4. Pick and Fit of Summed Isotope Distributions

5. Concentration Determination

1. Mass List Generation

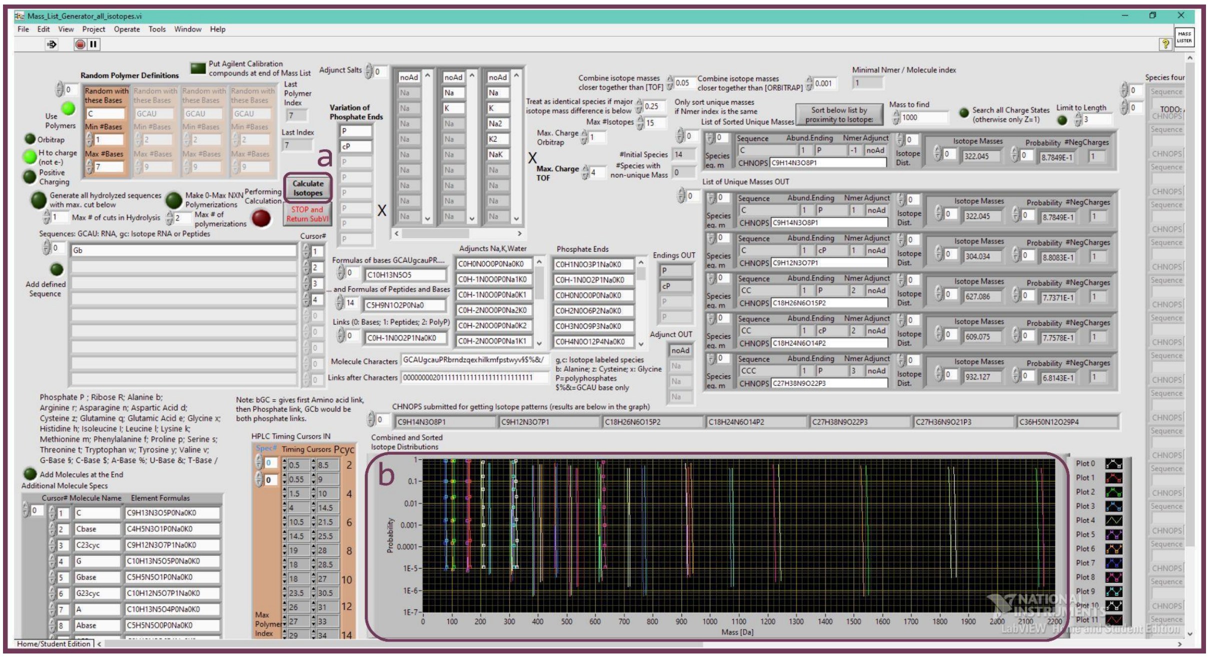

Define Masses: A sub-VI called "mass list generator" takes inputs such as nucleotide combinations, length range, charge states, and types of phosphate ends to generate a list of all chemical formulae **(Calculate Isotopes, a)**. This list is then passed via ZeroMQ message transfer protocol to Python, where IsoSpecPy [2] retrieves **theoretical isotope distributions (b)** for the corresponding chemical formulae.

2. Data Loading and Mass Chromatogram Plotting

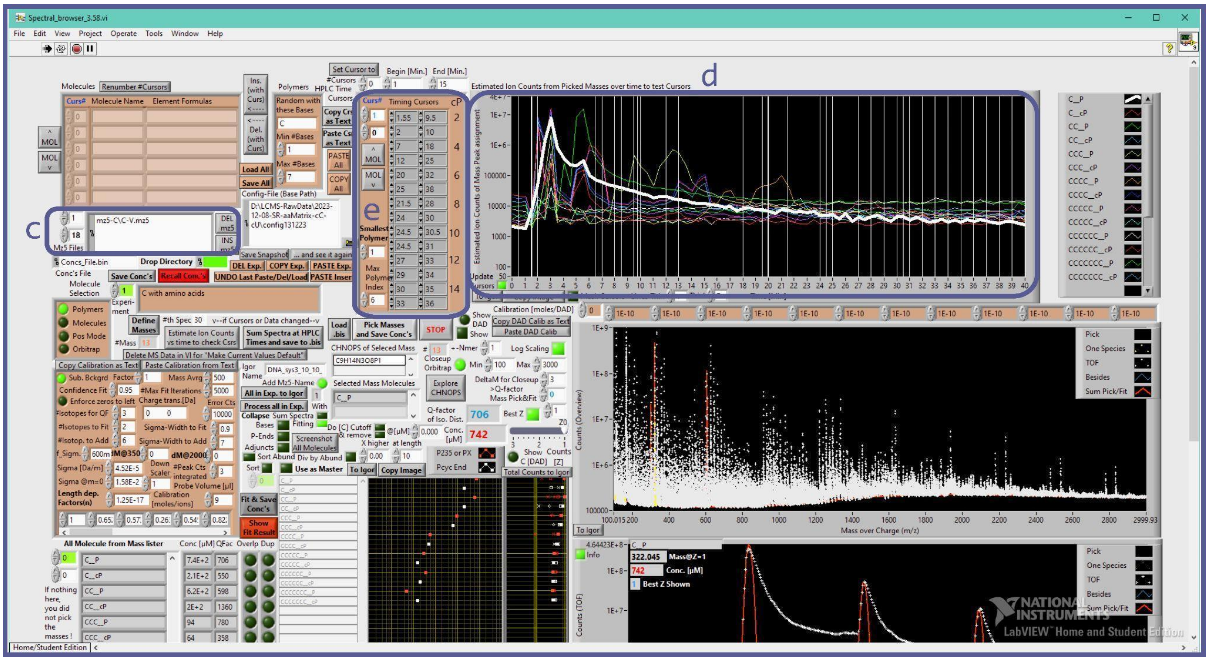

In the main VI, the **mz5 file is loaded (c)**, and mass chromatograms for the molecules are plotted **(Estimated Ion Counts, d)**. This step facilitates the determination of retention times

for different oligonucleotide lengths. The retention times of oligonucleotide standards are utilised to set **cursor positions (e)** for different-length oligomers.

3. Summation of Raw Mass Spectra

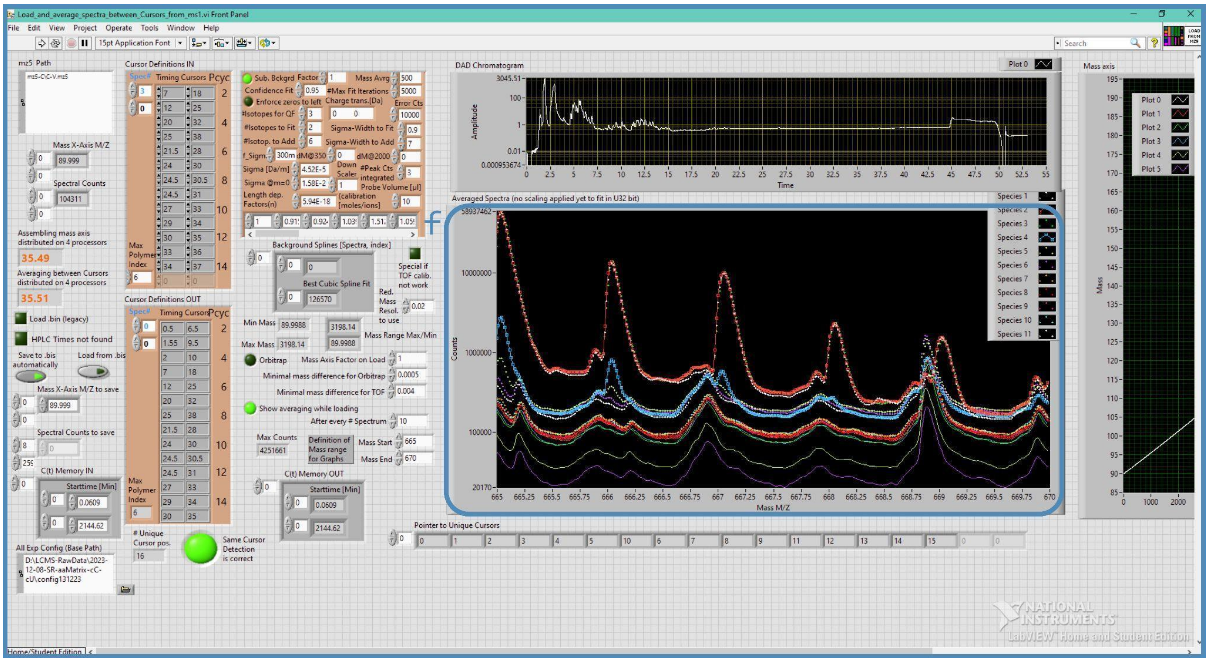

Raw mass spectra within the cursor positions are summed **(Sum Spectra, f)**. Additionally, the program saves the summed spectra into a binary file format (.bis), which optimises memory usage and enhances data loading time for future analysis. This feature allows for efficient retrieval and utilisation of the summed spectra in subsequent analyses through the **"load bis"** functionality.

4. Pick and Fit of Summed Isotope Distributions

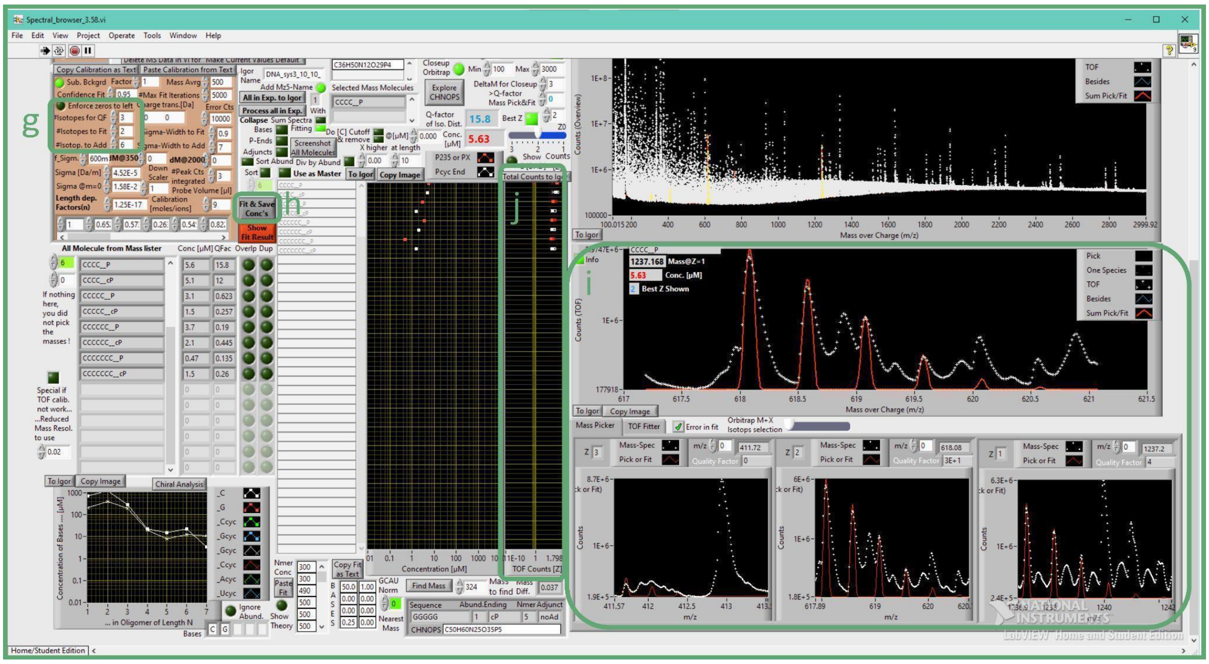

The theoretical isotope distributions for the molecules at the cursor positions are assigned to the corresponding m/z in the summed spectra using the most abundant isotope **(Pick Masses)**. Depending on the number of isotopes selected **(# isotope to fit, g)**, their theoretical distribution is fit to the measured data **(Fit & Save Conc, h)** to generate the **TOF counts (i, j).** The sum of TOF ion counts at different charge states is calculated for each molecule based on the fitted distributions.

5. Concentration Determination

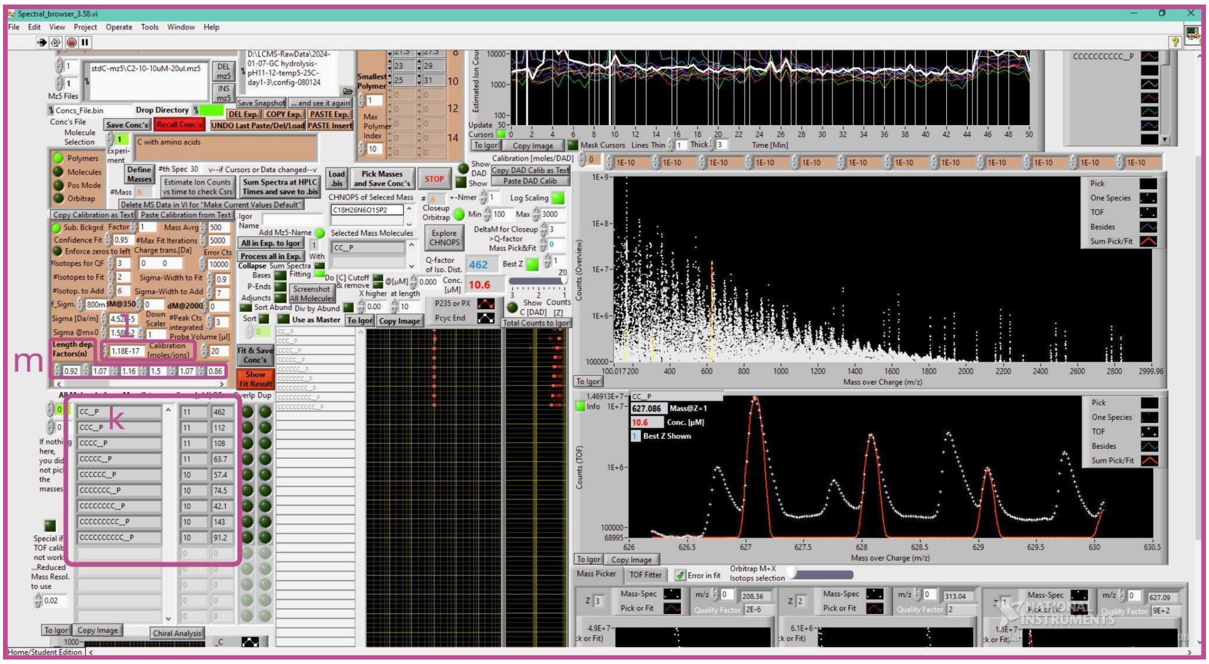

The concentration of the oligonucleotides in the sample is determined for the volume analysed using the TOF counts for **oligonucleotide standards of 2-10 mer (k)**. Calibration curves (moles/ion counts) for each of the standard oligos are generated for known concentrations. The mean of the slopes **(Calibration, moles/ions, l)** is calculated, and the corresponding ratio to the mean for each length is used as **length dependent factors (m)**.

NMR measurements

The dry-state reactions of Nucleoside 2', 3'-cyclic monophosphates were carried out in the presence of 5 equiv. valine under pH 10 conditions (adjusted with KOH) at room temperature for 20 h. The oligomers were solubilised in water and 8M urea with 5% D_2O and subjected to NMR measurements. The 1D- ^{31}P NMR spectra were acquired on a Bruker Avance III 300 MHz spectrometer. The data analysis was performed on Bruker TopSpin 4.3.0 to quantify the regio-isomers (3'-5' and 2'-5' linkages) in the RNA products. The peak assignments were done by comparison with the details presented in Ref 3.

Classical quantum chemical calculations

Quantum chemical calculations were performed using a simplified model of the reaction complex in which ethanol and β-1-dimethylamino-ribose-2',3'-cyclic phosphate served as substitutes for the nucleotides acting as nucleophile and substrate, respectively, in the transphosphorylation reaction. The model also included two glycine molecules: one in zwitterionic form, and another in the -1 charged anionic form. Given that the reaction's optimum pH is 10 (close to the pKa of glycine's amino group), the ratio of zwitterionic to amino forms of glycine is approximately 1:1, resulting in a total charge of -2 for the entire model system.

We justify the use of the simplified model described above as a similar model has been used in Ref 4 to model enzyme-catalyzed RNA-cleavage transesterification reactions (reverse reaction of the phosphodiester bond formation studied in the current work). The activation energies reported in Ref 3 were in excellent agreement with the experimentally obtained values in Ref 5.

Preliminary calculations were performed with the 6-31+G** basis set of atomic orbitals using Becke's three parameter exchange[6] and Lee-Yang-Parr's correlation functional[7]. Stationary points found with this approximation were used as starting geometries in higher level calculations performed with the ωB97XD long-range-corrected density functional[8] combined with the def2TZVPD basis set[9-10]. A very similar approach has been successfully applied for computational modelling of the polymerisation of 2',3' cyclic nucleotides without added catalysts in Ref 11. All computations were executed with the Gaussian09 program package[12]. All intermediates and transition states found were verified with frequency calculations. We note that the extreme flatness of the potential energy surface around the transition states and high-energy intermediates made the calculations very time-consuming: this is the reason why we have not attempted the calculation of the free energy profiles for other amino acids.

Two sets of calculations were conducted, differing in the hydrophobicity of the bulk medium represented by the PCM dielectric solvation model[13]. As a more hydrophobic environment, we have considered solvation in cyclohexane (ε=2.0), whereas a partially hydrated (i.e. more hydrophilic) environment was modeled assuming solvation in dimethylsulfoxide (ε=46.8).

Classical molecular dynamics simulations

Attempts to construct computational models for molecular dynamics simulations

Our initial attempts to employ standard molecular dynamics (MD) simulation protocols were unsuccessful due to the very small size and unusual composition of the systems. In explicit-solvent simulations using periodic boundary conditions (PBC), we observed that the amino acids interacted preferentially with other amino acids from neighbouring periodic cells rather than the nucleotide. This interaction pattern does not reflect the dynamics of a real system but is instead an artefact caused by the limited number of water molecules, resulting in small box sizes and high solute concentrations. *In our second attempt*, we tried to avoid the PBC issue by using a continuum solvent model for the simulations. While this approach effectively eliminated the border bias seen with PBC, the absence of explicit solvent molecules masked

any differences between individual amino acids. This strongly indicated that explicitly representing water molecules is essential in this case to accurately reproduce experimental observations. *For our third attempt*, we constructed the system using established protocols to create a sphere of explicit water molecules around the solutes while maintaining the sphere's integrity with a capping potential. However, similar to the PBC simulations, we encountered severe border bias as the amino acids preferentially interacted with the border area and the vacuum beyond the sphere rather than the nucleotide.

System building and simulation protocol

Finally, the following attempt appeared to be successful in performing the simulations presented in the paper.

To address the need for explicit representation of solvent molecules while also avoiding the border bias, we constructed systems with cytidine 2',3'-cyclic monophosphate (cCMP) surrounded by fifty water molecules. The partial charges for the cyclic phosphate moiety were obtained by first calculating the electrostatic potential of the phosphate with two of the oxygens joined by a -CH_2-CH_2- group using Gaussian 09 (rev. A02)[12] and HF/6-31G* level of theory. Finally, this was followed by partial-charge fitting and residue assignment in antechamber via the RESP procedure.[14-15] The finally fitted partial charges obtained for the individual atoms were: P 1.253301, OP1/OP2 -0.792882, O2' -0.528835, and O3' -0.548001. The rest of the nucleotide was described using standard partial charges from the OL3[16] RNA force field. For systems exploring interactions with amino acids and nucleobases, we added five amino acids and two nucleobases, respectively. The amino acids were modelled in zwitterionic form, while the nucleobases were constructed by removing phosphate and sugar atoms from the standard nucleotides and adding a methyl group cap on the N9 (purines) or N1 (pyrimidines) atoms. The partial charges of the methyl group caps were manually adjusted to obtain a zero net-charge for the nucleobases. The minimal amount of sodium or chloride ions[17] was added to obtain net-neutral conditions. This corresponded to one sodium ion, except for the systems with charged amino acids. We used the OL3[16] and ff14SB[18] force fields to describe the nucleotides and amino acids, respectively. The partial atomic charges for zwitterionic amino acids were obtained from Ref. 19 with the residue libraries adjusted to utilise the ff14SB dihedrals.

In total, sixteen different systems were constructed, with twelve involving amino acids and four involving nucleobases. The initial positions of the molecules surrounding the cCMP were randomly assigned to be as close as possible to the cCMP while avoiding steric overlaps. To keep the surrounding molecules in the vicinity of the nucleotides, we applied linear distance potentials with a force constant of 1000 kcal/mol/Å. This resulted in a steep force potential acting above 12 Å for the water molecules and ions (defined as the distance between the ions/water oxygen atoms and the C1' atom of the cCMP) and beyond 10 Å for the amino acids and nucleobases (defined as the distance between the CA and C1' atoms of the amino acids and nucleobases, respectively, and the C1' atom of the cCMP). Shorter distances for the amino acids and nucleobases were applied due to their larger bulk size and to minimise the border bias. The systems constructed in this fashion were finally immersed in continuum solvent[20] to further reduce the border bias. All the systems were minimised and equilibrated using the sander.MPI module of AMBER22[21]. We used 1000 steps of steepest descent method, followed by 1000 steps of conjugated gradient minimisation. Production simulations were then performed for 1 µs using the GPU-accelerated pmemd.cuda module[22-23]. A Langevin thermostat was used to maintain the temperature of the systems at 300 K. The

cut-off distance for nonbonded interactions was set to infinity, and the SHAKE algorithm[24] was utilised, allowing the use of a 2 fs integration step.

Analysis of the MD simulations

All the trajectories were extensively visually analysed using the VMD software[25]. The systems with amino acids were evaluated by analysing the interactions between the amino acid backbone charged amino groups and the different parts of the system. The *hbond* command of cpptraj[26] was utilised for this purpose. For the systems with nucleobases, we analysed the H-bonds formed between the nucleobases and cCMP using the *hbond* command of cpptraj with default settings.

***Ab initio* molecular dynamics**

We utilised the CP2K[27] software package, which is based on the Born-Oppenheimer approach, to perform *ab initio* molecular dynamics (AIMD) simulations. Each system comprised two 2',3'- cyclic CMP nucleotide anions, two amino acid molecules and a minimum number of water molecules to approximate the hydration state of the dry amorphous solid materials used in the experiments (~ 5-10 water molecules/nucleotide). Thus, our model systems, with the exception of Asp, included 22 water molecules and 3 charge-compensating Na^+ ions. Due to the anionic side chain of Asp, in this latter case, 24 water molecules and 5 charge-compensating Na^+ ions were included in the computational model. The starting arrangement of the nucleotides and amino acids was derived from the optimised geometry of the reaction complex with glycine, obtained from preliminary calculations performed at Becke3LYP/6-31+G** level of theory for the simplified model described in the "Classical quantum chemical calculations" section. For the amino acids Pro and Arg we were unable to define starting geometries using this method due to significant steric clashes of the side chains. The simulated models were embedded in rectangular parallelepiped cells with side parameters equal to 40.00, 25.00, and 25.00 Å along the x, y, and z Cartesian axes, respectively. Each simulated system underwent dynamics for at least 10 ps, resulting in a cumulative global simulation time exceeding 100 ps.

Wavefunctions of the atomic species were expanded in the TZVP basis set with Goedecker-Teter-Hutter (GTH) pseudopotentials using the GPW method[28]. A plane-wave cutoff of 400 Ry was applied. Exchange and correlation (XC) effects were treated with the gradient-corrected Becke-Lee-Yang-Parr (BLYP)[7,29] density functional. To account for dispersion interactions, we employed the dispersion-corrected version of BLYP (i.e., BLYP+D3(BJ))[30-31]. A nominal temperature of 300 K has been simulated. The dynamics of ions were simulated classically within a constant number, volume, and temperature (NVT) ensemble using the Verlet algorithm. Canonical sampling was performed using a canonical sampling-through-velocity-rescaling thermostat (CSVR)[32], set with a time constant equal to 50 fs.

Supplementary References

1. Kessner, D. et al. ProteoWizard: open source software for rapid proteomics tools development. *Bioinformatics*, 24, 2534-2536 (2008).
2. Łącki, M. K., Valkenborg, D. & Startek, M. P. Isospec2: Ultrafast fine structure calculator. *Anal. Chem*. 92, 9472–9475 (2020).
3. Motsch, S., Tremmel, P. & Richert, C. Regioselective Formation of RNA strands in the absence of magnesium ions. *Nucleic Acids Research* 48, 1097–1107 (2019).
4. Wong et al. Characterization of the reaction path and transition States for RNA transphosphorylation models from theory and experiment. *Angew. Chem. Int. Ed*. 51, 647–651 (2012)
5. Harris et al. Kinetic Isotope Effects for RNA Cleavage by 2′-O- Transphosphorylation: Nucleophilic Activation by Specific Base. *J. Am. Chem. Soc*. 132, 11613–11621 (2010)
6. Becke, A. D. Density-functional thermochemistry. III. The role of exact exchange. *J. Chem. Phys*. 98, 5648-5652 (1993).
7. Lee, C., Yang, W. & Parr, R. G. Development of the Colle-Salvetti correlation-energy formula into a functional of the electron density. *Phys. Rev*. B 37, 785-789 (1988).
8. Chai, J.-D. & Head-Gordon, M. Systematic optimization of long-range corrected hybrid density functionals. *J. Chem. Phys*. 128, 084106 (2008).
9. Rappoport, D. & Furche, F. Property-optimized Gaussian basis sets for molecular response calculations. *J. Chem. Phys*. 133, 134105 (2010).
10. Weigend, F. & Ahlrichs, R. Balanced basis sets of split valence, triple zeta valence and quadruple zeta valence quality for H to Rn: design and assessment of accuracy. *Phys. Chem. Chem. Phys*. 7, 3297-3305 (2005).
11. Dass, A. V. et al. RNA oligomerisation without added catalyst from 2′,3′-cyclic nucleotides by drying at air-water interfaces. *ChemSystemsChem* 5, e202200026 (2023).
12. Gaussian 09, Revision A.02 (Gaussian, Inc., Wallingford CT, 2016).
13. Tomasi, J., Mennucci, B. & Cammi, R. Quantum mechanical continuum solvation models. *Chem. Rev*. 105, 2999-3094 (2005).
14. Cornell, W. D., Cieplak, P., Bayly, C. I., & Kollman, P. A. Application of RESP charges to calculate conformational energies, hydrogen bond energies, and free energies of solvation. *J. Am. Chem. Soc.*, 115, 9620–9631 (1993).
15. Bayly, C. I., Cieplak, P., Cornell, W., & Kollman, P. A. A well-behaved electrostatic potential based method using charge restraints for deriving atomic charges: the RESP model. *J. Phys. Chem.*, 97, 10269–10280 (1993).
16. Zgarbova, M. et al. Refinement of the Cornell et al. nucleic acids force field based on reference quantum chemical calculations of glycosidic torsion profiles. *J. Chem. Theory Comput.* 7, 2886-2902 (2011).
17. Joung, I. S. & Cheatham, T. E. Determination of alkali and halide monovalent ion parameters for use in explicitly solvated biomolecular simulations. *J. Phys. Chem. B* 112, 9020-9041 (2008).
18. Maier, J. A. et al. ff14SB: Improving the accuracy of protein side chain and backbone parameters from ff99sb. *J. Chem. Theory Comput.* 11, 3696-3713 (2015).
19. Horn, A. H. C. A consistent force field parameter set for zwitterionic amino acid residues. *J. Mol. Model*. 20, 2478 (2014).

20. Hawkins, G. D., Cramer, C. J. & Truhlar, D. G. Parametrized models of aqueous free energies of solvation based on pairwise descreening of solute atomic charges from a dielectric medium. *J. Phys. Chem*. 100, 19824-19839 (1996).
21. D.A. Case et al. Amber 22. (2022).
22. Le Grand, S., Götz, A. W. & Walker, R. C. SPFP: Speed without compromise—a mixed precision model for GPU accelerated molecular dynamics simulations. *Comput. Phys. Commun.* 184, 374-380 (2013).
23. Gotz, A. W. et al. Routine microsecond molecular dynamics simulations with AMBER on GPUs. 1. Generalized Born. *J. Chem. Theory. Comput*. 8, 1542-1555 (2012).
24. Ryckaert, J. P., Ciccotti, G. & Berendsen, H. J. C. Numerical-integration of cartesian equations of motion of a system with constraints - molecular-dynamics of n-alkanes. *J. Comput. Phys*. 23, 327-341 (1977).
25. Humphrey, W., Dalke, A. & Schulten, K. VMD: visual molecular dynamics. *J. Mol. Graph.* 14, 33-38 (1996).
26. Roe, D. R. & Cheatham, T. E. PTRAJ and CPPTRAJ: software for processing and analysis of molecular dynamics trajectory data. *J. Chem. Theory Comput*. 9, 3084-3095 (2013).
27. Kühne, T. D. et al. CP2K: An electronic structure and molecular dynamics software package - Quickstep: Efficient and accurate electronic structure calculations. *J. Chem. Phys.* 152, 194103 (2020).
28. Krack, M. Pseudopotentials for H to Kr optimized for gradient-corrected exchange-correlation functionals. *Theor. Chem. Acc*. 114, 145-152 (2005).
29. Becke, A. D. Density-functional exchange-energy approximation with correct asymptotic behavior. *Phys. Rev. A* 38, 3098-3100 (1988).
30. Grimme, S., Antony, J., Ehrlich, S. & Krieg, H. A consistent and accurate ab initio parametrization of density functional dispersion correction (DFT-D) for the 94 elements H-Pu. *J. Chem. Phys*. 132, 154104 (2010).
31. Grimme, S., Ehrlich, S. & Goerigk, L. Effect of the damping function in dispersion corrected density functional theory. *J. Comput. Chem*. 32, 1456-1465 (2011).
32. Bussi, G., Donadio, D. & Parrinello, M. Canonical sampling through velocity rescaling. *J. Chem. Phys*. 126, 014101 (2007).
