# Supplementary material for: Amino acids catalyse RNA formation under ambient alkaline conditions
Source: Nat Commun. 2025 Jun 4;16:5193. doi: 10.1038/s41467-025-60359-3 (PMC12137669; doi:10.1038/s41467-025-60359-3)
Supplement: Supplementary file 2 — Description of Additional Supplementary Files [file 41467_2025_60359_MOESM2_ESM.pdf]

Description of Additional Supplementary Files

Supplementary Data 1: Mass spectra of all RNA products, along with corresponding isotope distribution fits, related to Figure 1.

Supplementary Data 2: Mass spectra of all RNA products, along with corresponding isotope distribution fits, related to Figure 2.

Supplementary Data 3: Mass spectra of all RNA products, along with corresponding isotope distribution fits, related to Figure 4.
