# Supplementary figures and images for: Amino acids catalyse RNA formation under ambient alkaline conditions (part 2 of 2)
Source: Nat Commun. 2025 Jun 4;16:5193. doi: 10.1038/s41467-025-60359-3 (PMC12137669; doi:10.1038/s41467-025-60359-3)

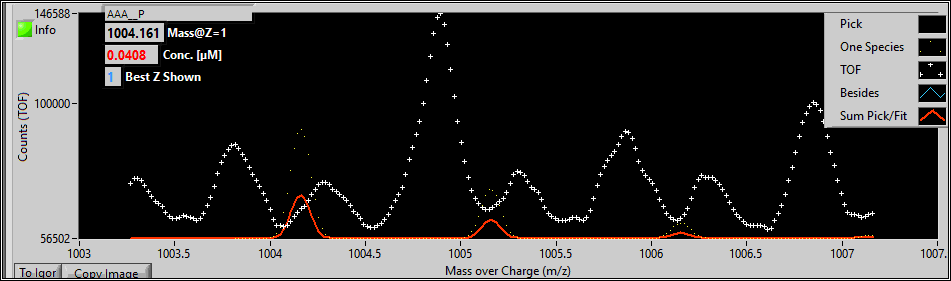

Supplement: Supplementary file 3 — Supplementary Data 1, 2 and 3 [file 41467_2025_60359_MOESM3_ESM.zip › Supplementary Data/Supplementary Data 3/AU-no aa/000006_AAA__P.bmp]

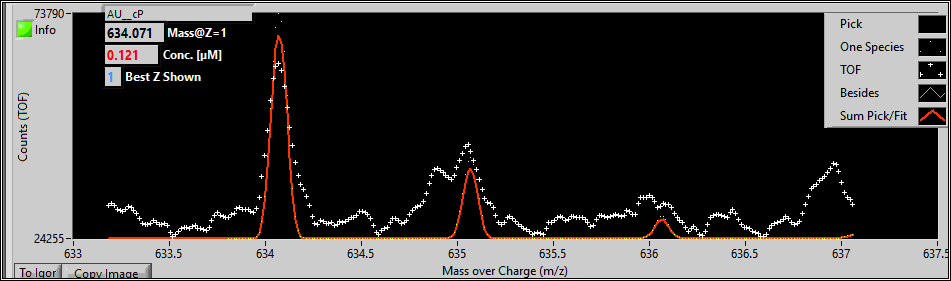

Supplement: Supplementary file 3 — Supplementary Data 1, 2 and 3 [file 41467_2025_60359_MOESM3_ESM.zip › Supplementary Data/Supplementary Data 3/AU-no aa/000003_AU__cP.bmp]

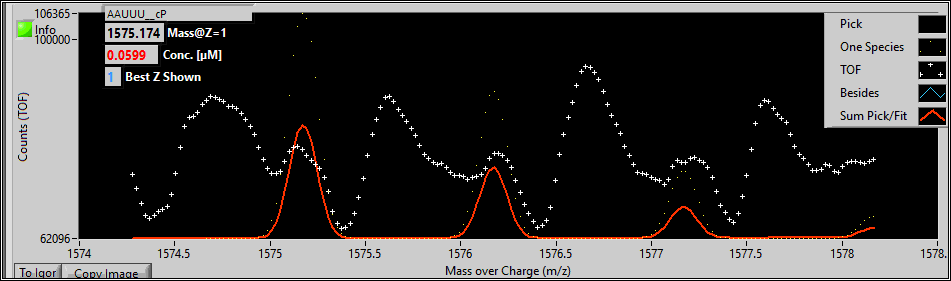

Supplement: Supplementary file 3 — Supplementary Data 1, 2 and 3 [file 41467_2025_60359_MOESM3_ESM.zip › Supplementary Data/Supplementary Data 3/AU-no aa/000031_AAUUU__cP.bmp]

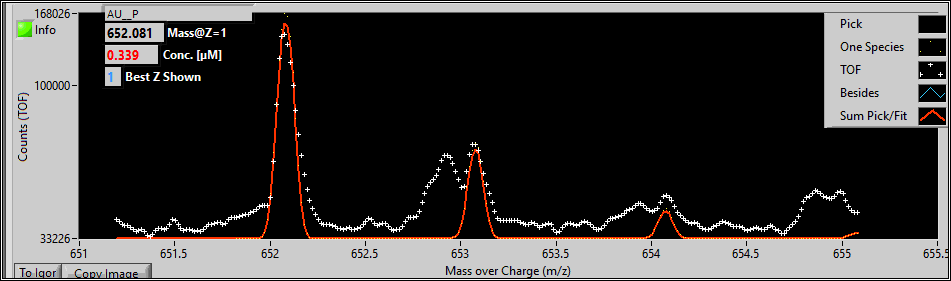

Supplement: Supplementary file 3 — Supplementary Data 1, 2 and 3 [file 41467_2025_60359_MOESM3_ESM.zip › Supplementary Data/Supplementary Data 3/AU-no aa/000002_AU__P.bmp]

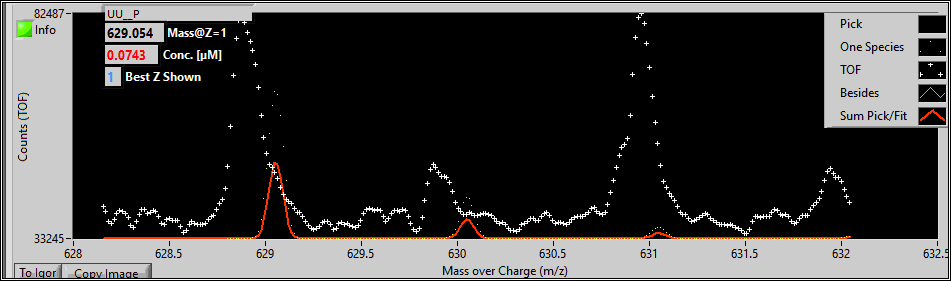

Supplement: Supplementary file 3 — Supplementary Data 1, 2 and 3 [file 41467_2025_60359_MOESM3_ESM.zip › Supplementary Data/Supplementary Data 3/AU-no aa/000004_UU__P.bmp]

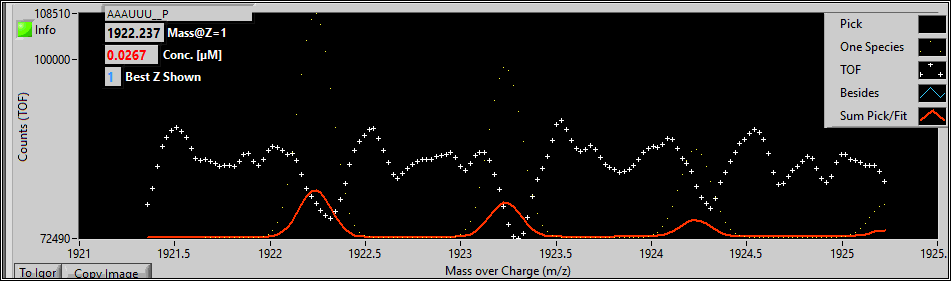

Supplement: Supplementary file 3 — Supplementary Data 1, 2 and 3 [file 41467_2025_60359_MOESM3_ESM.zip › Supplementary Data/Supplementary Data 3/AU-no aa/000042_AAAUUU__P.bmp]

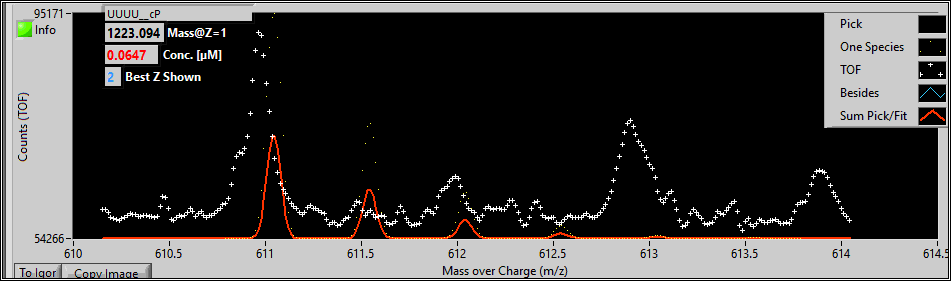

Supplement: Supplementary file 3 — Supplementary Data 1, 2 and 3 [file 41467_2025_60359_MOESM3_ESM.zip › Supplementary Data/Supplementary Data 3/AU-no aa/000023_UUUU__cP.bmp]

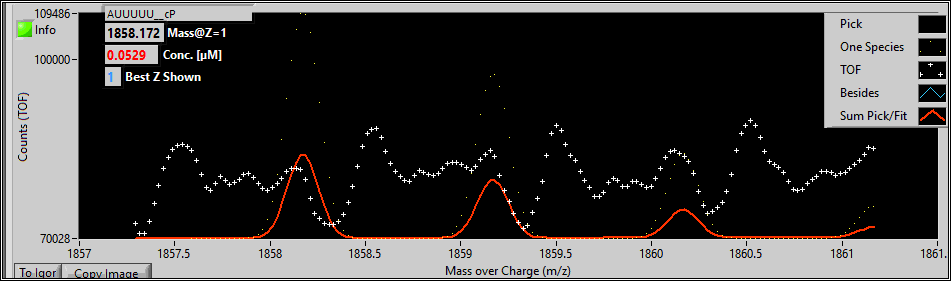

Supplement: Supplementary file 3 — Supplementary Data 1, 2 and 3 [file 41467_2025_60359_MOESM3_ESM.zip › Supplementary Data/Supplementary Data 3/AU-no aa/000047_AUUUUU__cP.bmp]

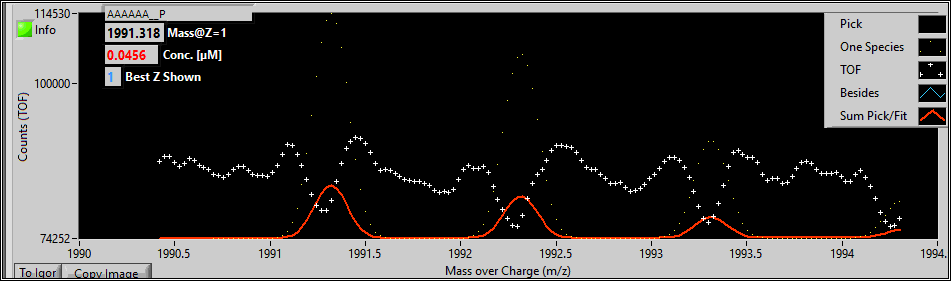

Supplement: Supplementary file 3 — Supplementary Data 1, 2 and 3 [file 41467_2025_60359_MOESM3_ESM.zip › Supplementary Data/Supplementary Data 3/AU-no aa/000036_AAAAAA__P.bmp]

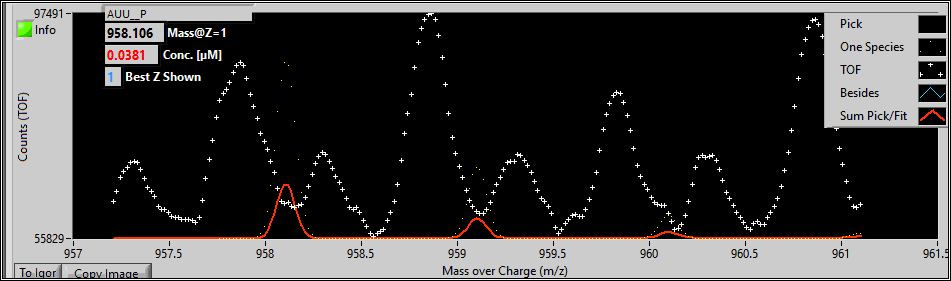

Supplement: Supplementary file 3 — Supplementary Data 1, 2 and 3 [file 41467_2025_60359_MOESM3_ESM.zip › Supplementary Data/Supplementary Data 3/AU-no aa/000010_AUU__P.bmp]

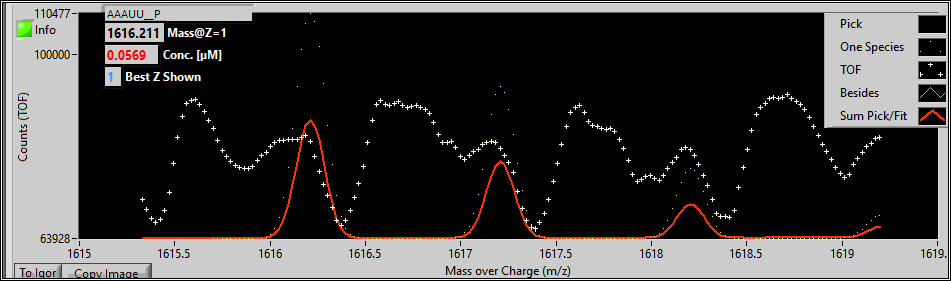

Supplement: Supplementary file 3 — Supplementary Data 1, 2 and 3 [file 41467_2025_60359_MOESM3_ESM.zip › Supplementary Data/Supplementary Data 3/AU-no aa/000028_AAAUU__P.bmp]

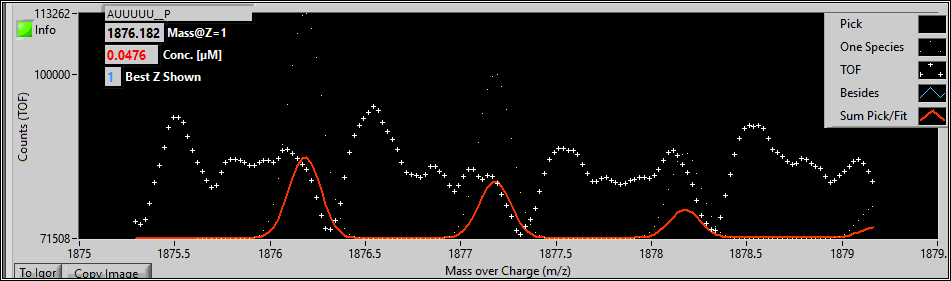

Supplement: Supplementary file 3 — Supplementary Data 1, 2 and 3 [file 41467_2025_60359_MOESM3_ESM.zip › Supplementary Data/Supplementary Data 3/AU-no aa/000046_AUUUUU__P.bmp]

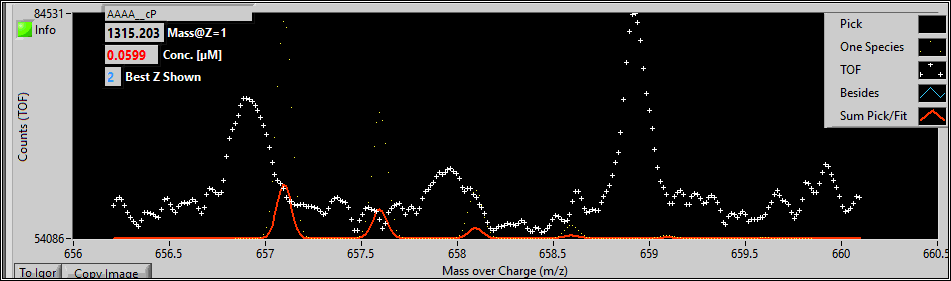

Supplement: Supplementary file 3 — Supplementary Data 1, 2 and 3 [file 41467_2025_60359_MOESM3_ESM.zip › Supplementary Data/Supplementary Data 3/AU-no aa/000015_AAAA__cP.bmp]

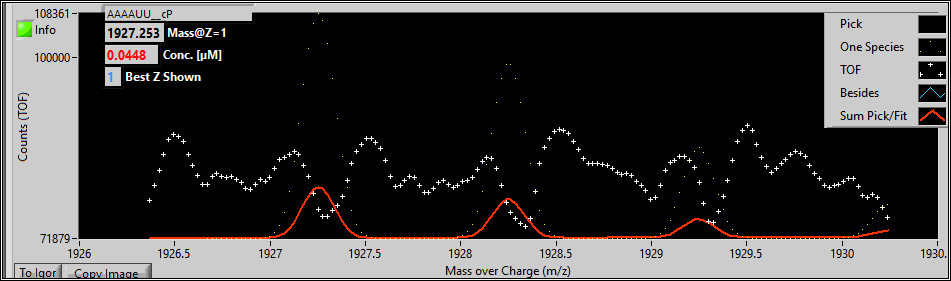

Supplement: Supplementary file 3 — Supplementary Data 1, 2 and 3 [file 41467_2025_60359_MOESM3_ESM.zip › Supplementary Data/Supplementary Data 3/AU-no aa/000041_AAAAUU__cP.bmp]

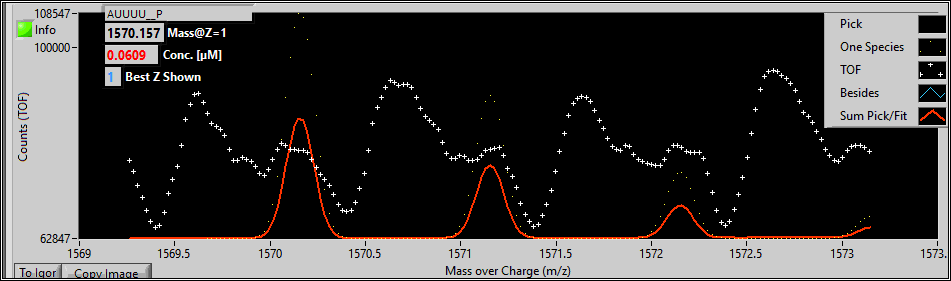

Supplement: Supplementary file 3 — Supplementary Data 1, 2 and 3 [file 41467_2025_60359_MOESM3_ESM.zip › Supplementary Data/Supplementary Data 3/AU-no aa/000032_AUUUU__P.bmp]

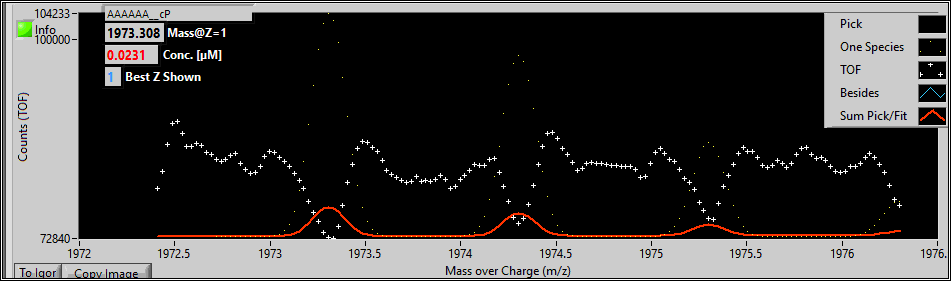

Supplement: Supplementary file 3 — Supplementary Data 1, 2 and 3 [file 41467_2025_60359_MOESM3_ESM.zip › Supplementary Data/Supplementary Data 3/AU-no aa/000037_AAAAAA__cP.bmp]

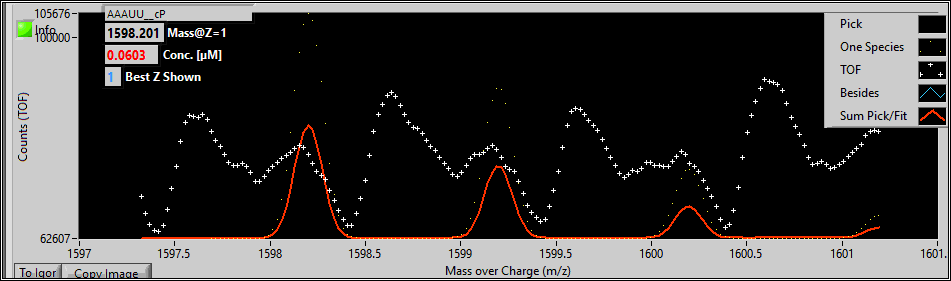

Supplement: Supplementary file 3 — Supplementary Data 1, 2 and 3 [file 41467_2025_60359_MOESM3_ESM.zip › Supplementary Data/Supplementary Data 3/AU-no aa/000029_AAAUU__cP.bmp]

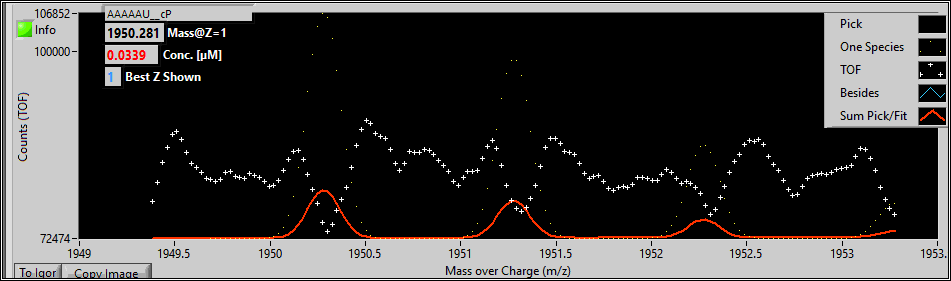

Supplement: Supplementary file 3 — Supplementary Data 1, 2 and 3 [file 41467_2025_60359_MOESM3_ESM.zip › Supplementary Data/Supplementary Data 3/AU-no aa/000039_AAAAAU__cP.bmp]

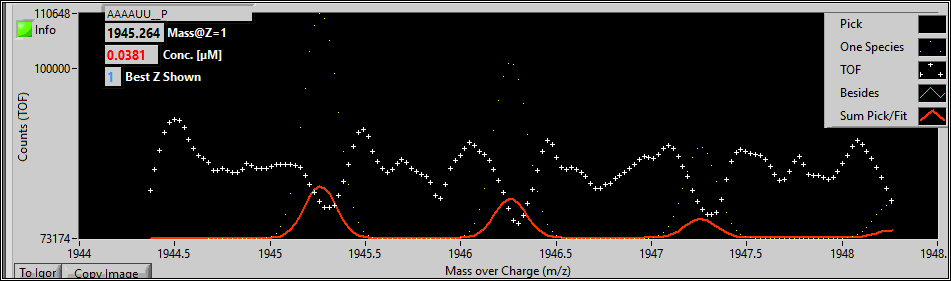

Supplement: Supplementary file 3 — Supplementary Data 1, 2 and 3 [file 41467_2025_60359_MOESM3_ESM.zip › Supplementary Data/Supplementary Data 3/AU-no aa/000040_AAAAUU__P.bmp]

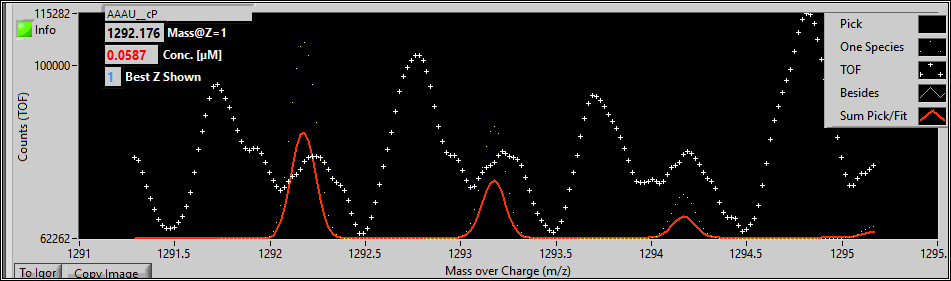

Supplement: Supplementary file 3 — Supplementary Data 1, 2 and 3 [file 41467_2025_60359_MOESM3_ESM.zip › Supplementary Data/Supplementary Data 3/AU-no aa/000017_AAAU__cP.bmp]

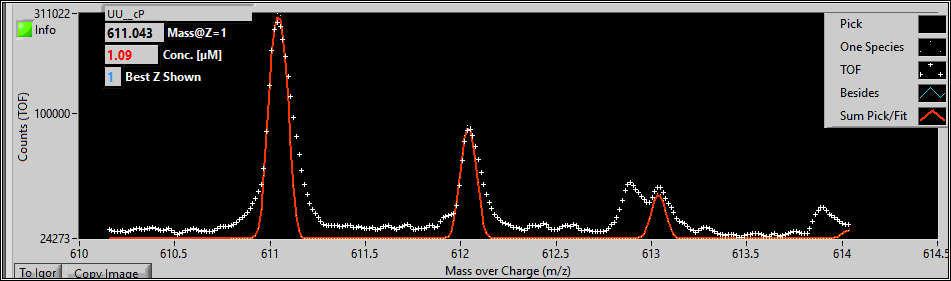

Supplement: Supplementary file 3 — Supplementary Data 1, 2 and 3 [file 41467_2025_60359_MOESM3_ESM.zip › Supplementary Data/Supplementary Data 3/AU-no aa/000005_UU__cP.bmp]

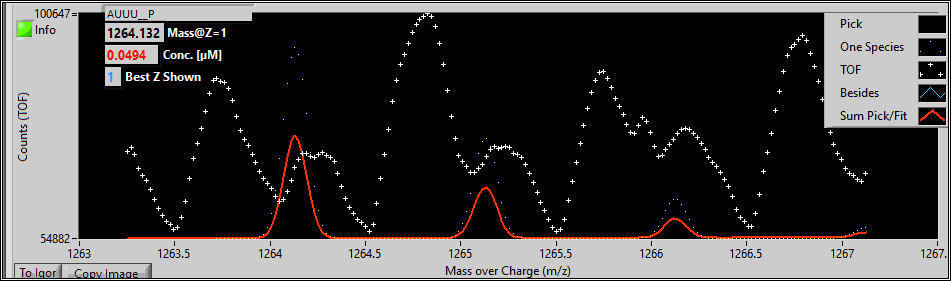

Supplement: Supplementary file 3 — Supplementary Data 1, 2 and 3 [file 41467_2025_60359_MOESM3_ESM.zip › Supplementary Data/Supplementary Data 3/AU-no aa/000020_AUUU__P.bmp]

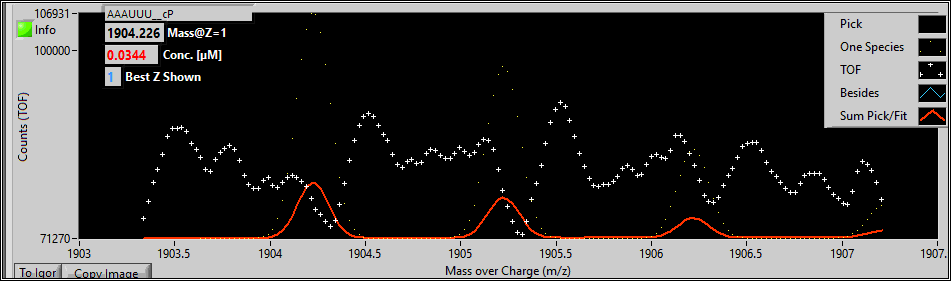

Supplement: Supplementary file 3 — Supplementary Data 1, 2 and 3 [file 41467_2025_60359_MOESM3_ESM.zip › Supplementary Data/Supplementary Data 3/AU-no aa/000043_AAAUUU__cP.bmp]

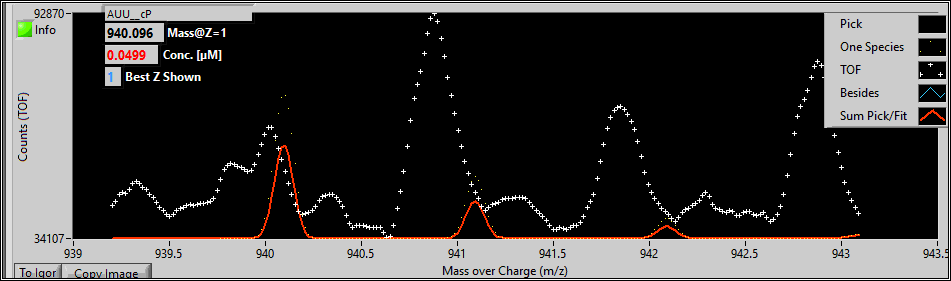

Supplement: Supplementary file 3 — Supplementary Data 1, 2 and 3 [file 41467_2025_60359_MOESM3_ESM.zip › Supplementary Data/Supplementary Data 3/AU-no aa/000011_AUU__cP.bmp]

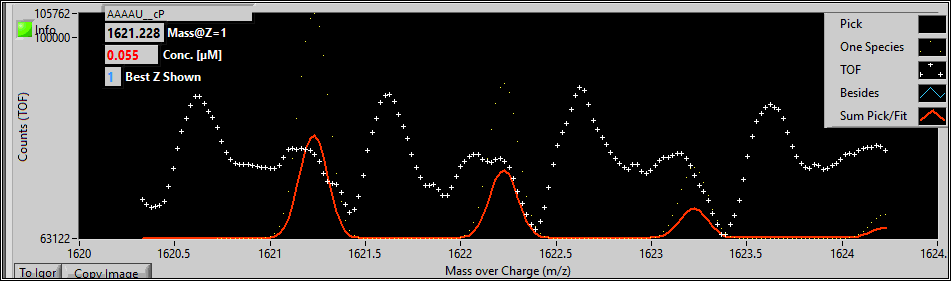

Supplement: Supplementary file 3 — Supplementary Data 1, 2 and 3 [file 41467_2025_60359_MOESM3_ESM.zip › Supplementary Data/Supplementary Data 3/AU-no aa/000027_AAAAU__cP.bmp]

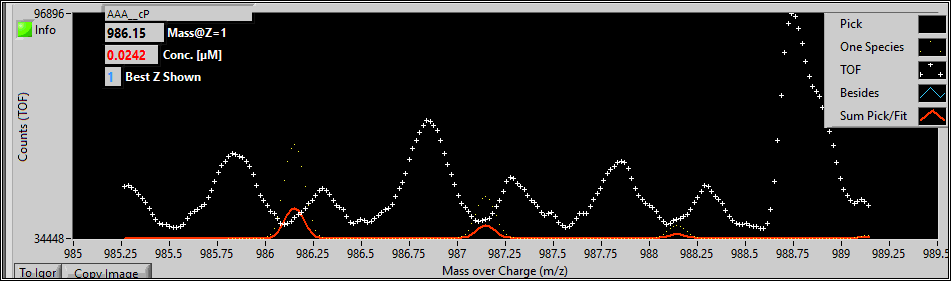

Supplement: Supplementary file 3 — Supplementary Data 1, 2 and 3 [file 41467_2025_60359_MOESM3_ESM.zip › Supplementary Data/Supplementary Data 3/AU-no aa/000007_AAA__cP.bmp]

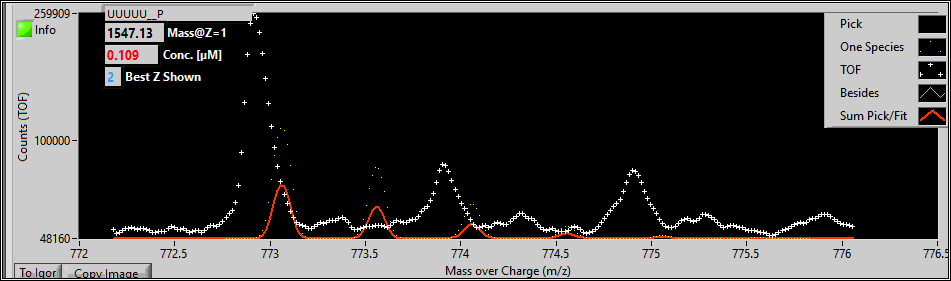

Supplement: Supplementary file 3 — Supplementary Data 1, 2 and 3 [file 41467_2025_60359_MOESM3_ESM.zip › Supplementary Data/Supplementary Data 3/AU-no aa/000034_UUUUU__P.bmp]

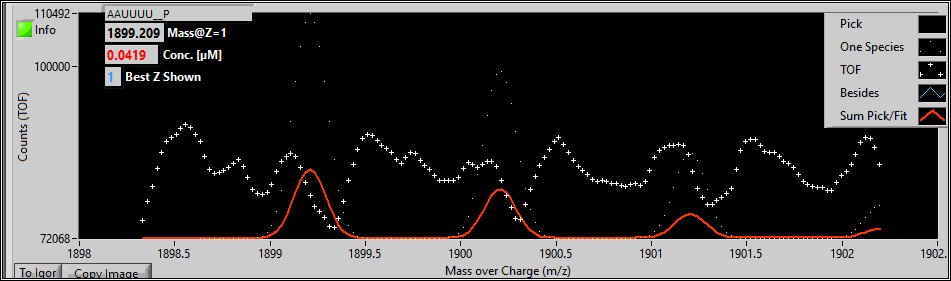

Supplement: Supplementary file 3 — Supplementary Data 1, 2 and 3 [file 41467_2025_60359_MOESM3_ESM.zip › Supplementary Data/Supplementary Data 3/AU-no aa/000044_AAUUUU__P.bmp]

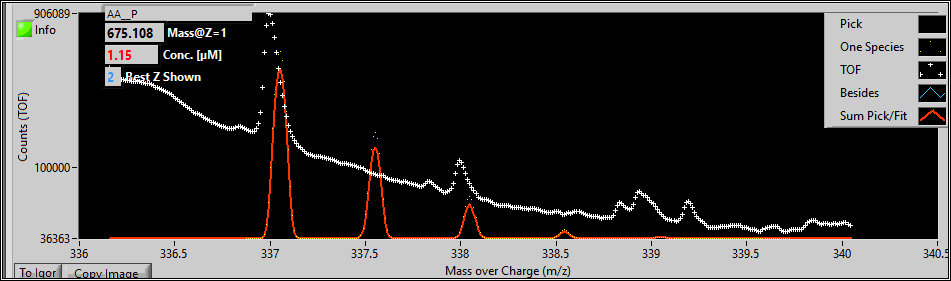

Supplement: Supplementary file 3 — Supplementary Data 1, 2 and 3 [file 41467_2025_60359_MOESM3_ESM.zip › Supplementary Data/Supplementary Data 3/AU-no aa/000000_AA__P.bmp]

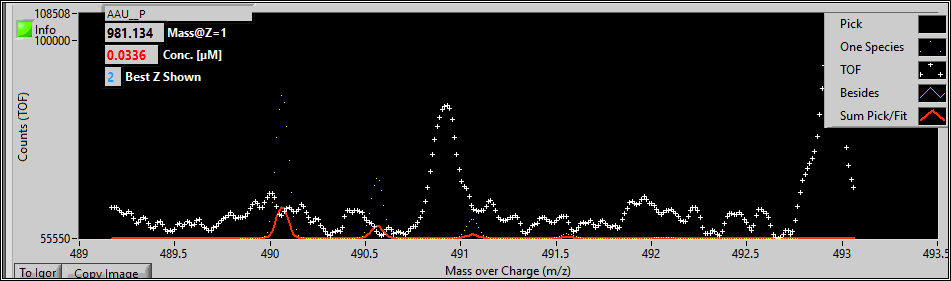

Supplement: Supplementary file 3 — Supplementary Data 1, 2 and 3 [file 41467_2025_60359_MOESM3_ESM.zip › Supplementary Data/Supplementary Data 3/AU-no aa/000008_AAU__P.bmp]

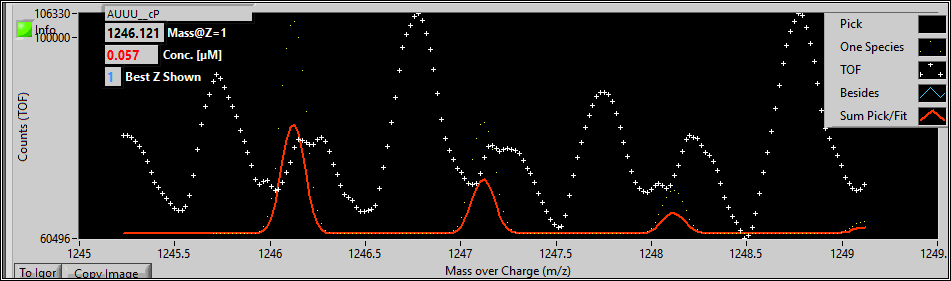

Supplement: Supplementary file 3 — Supplementary Data 1, 2 and 3 [file 41467_2025_60359_MOESM3_ESM.zip › Supplementary Data/Supplementary Data 3/AU-no aa/000021_AUUU__cP.bmp]

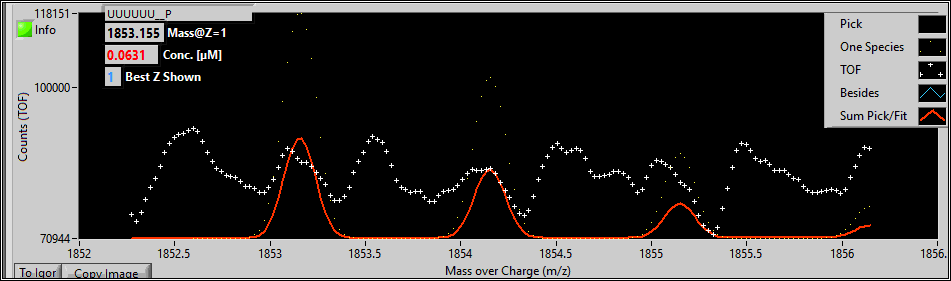

Supplement: Supplementary file 3 — Supplementary Data 1, 2 and 3 [file 41467_2025_60359_MOESM3_ESM.zip › Supplementary Data/Supplementary Data 3/AU-no aa/000048_UUUUUU__P.bmp]

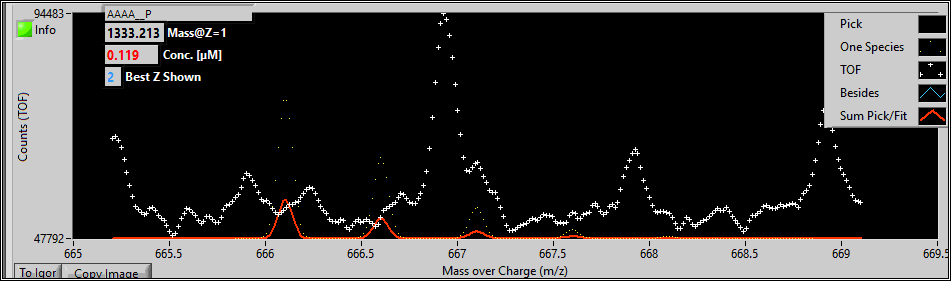

Supplement: Supplementary file 3 — Supplementary Data 1, 2 and 3 [file 41467_2025_60359_MOESM3_ESM.zip › Supplementary Data/Supplementary Data 3/AU-no aa/000014_AAAA__P.bmp]

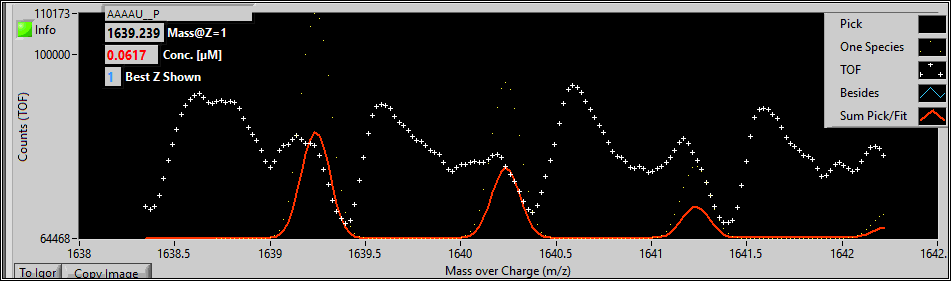

Supplement: Supplementary file 3 — Supplementary Data 1, 2 and 3 [file 41467_2025_60359_MOESM3_ESM.zip › Supplementary Data/Supplementary Data 3/AU-no aa/000026_AAAAU__P.bmp]

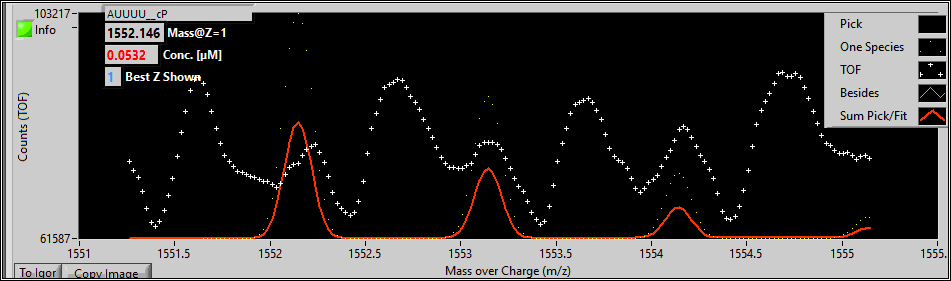

Supplement: Supplementary file 3 — Supplementary Data 1, 2 and 3 [file 41467_2025_60359_MOESM3_ESM.zip › Supplementary Data/Supplementary Data 3/AU-no aa/000033_AUUUU__cP.bmp]

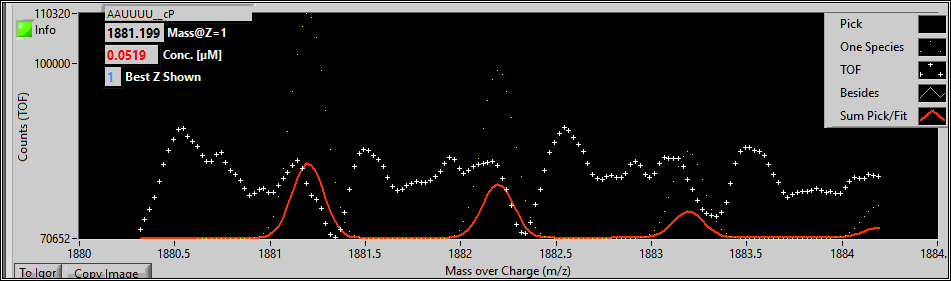

Supplement: Supplementary file 3 — Supplementary Data 1, 2 and 3 [file 41467_2025_60359_MOESM3_ESM.zip › Supplementary Data/Supplementary Data 3/AU-no aa/000045_AAUUUU__cP.bmp]

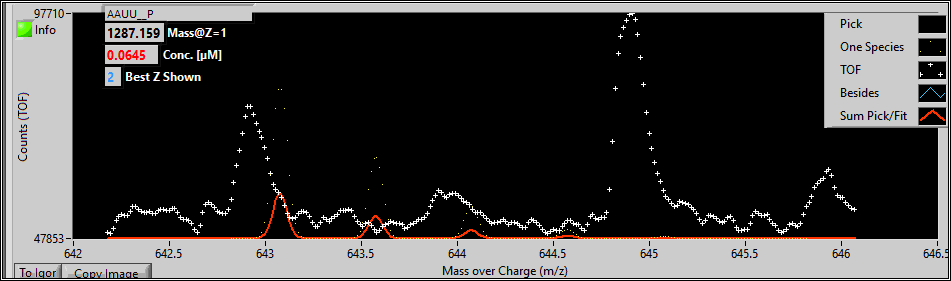

Supplement: Supplementary file 3 — Supplementary Data 1, 2 and 3 [file 41467_2025_60359_MOESM3_ESM.zip › Supplementary Data/Supplementary Data 3/AU-no aa/000018_AAUU__P.bmp]

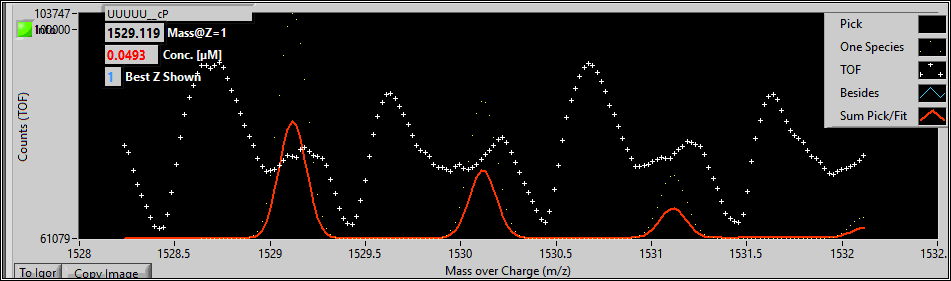

Supplement: Supplementary file 3 — Supplementary Data 1, 2 and 3 [file 41467_2025_60359_MOESM3_ESM.zip › Supplementary Data/Supplementary Data 3/AU-no aa/000035_UUUUU__cP.bmp]

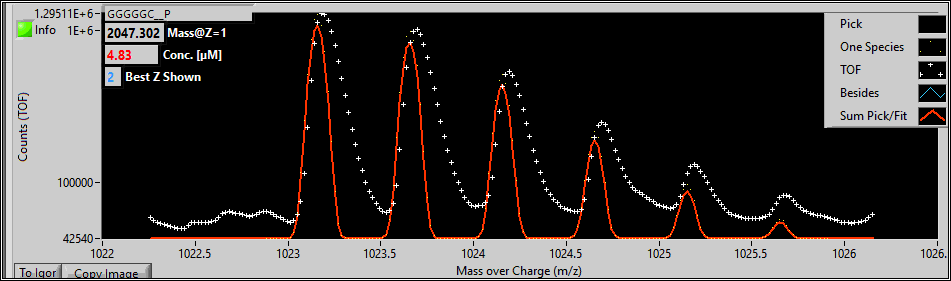

Supplement: Supplementary file 3 — Supplementary Data 1, 2 and 3 [file 41467_2025_60359_MOESM3_ESM.zip › Supplementary Data/Supplementary Data 3/GC-Val-SI/000042_GGGGGC__P.bmp]

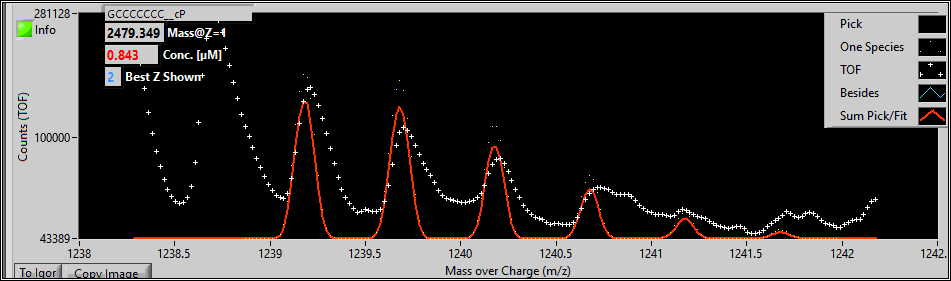

Supplement: Supplementary file 3 — Supplementary Data 1, 2 and 3 [file 41467_2025_60359_MOESM3_ESM.zip › Supplementary Data/Supplementary Data 3/GC-Val-SI/000085_GCCCCCCC__cP.bmp]

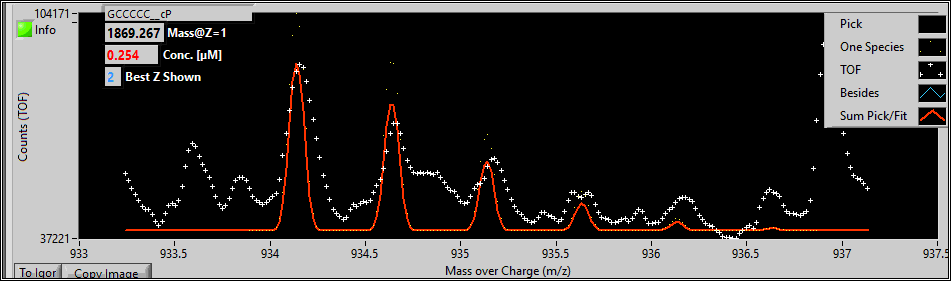

Supplement: Supplementary file 3 — Supplementary Data 1, 2 and 3 [file 41467_2025_60359_MOESM3_ESM.zip › Supplementary Data/Supplementary Data 3/GC-Val-SI/000051_GCCCCC__cP.bmp]

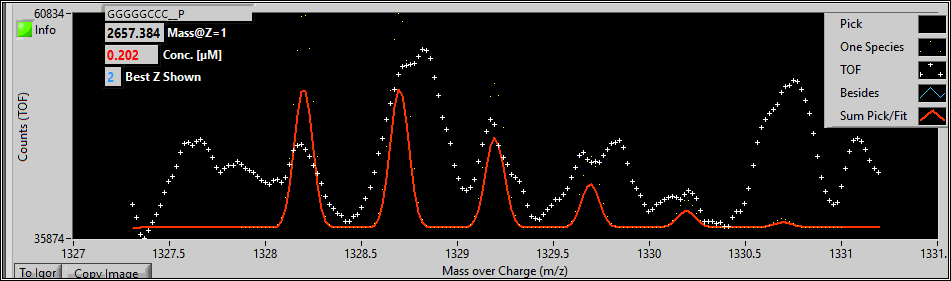

Supplement: Supplementary file 3 — Supplementary Data 1, 2 and 3 [file 41467_2025_60359_MOESM3_ESM.zip › Supplementary Data/Supplementary Data 3/GC-Val-SI/000076_GGGGGCCC__P.bmp]

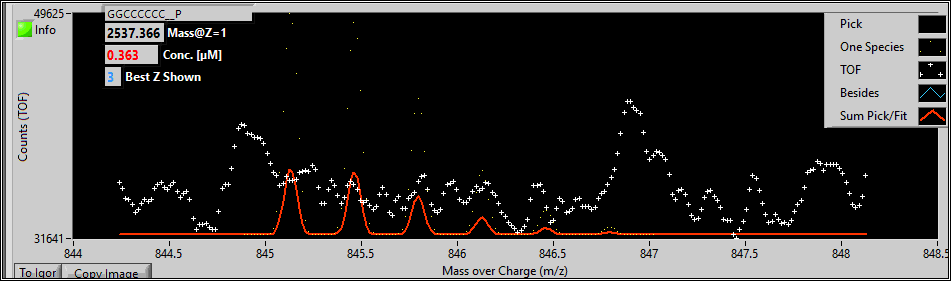

Supplement: Supplementary file 3 — Supplementary Data 1, 2 and 3 [file 41467_2025_60359_MOESM3_ESM.zip › Supplementary Data/Supplementary Data 3/GC-Val-SI/000082_GGCCCCCC__P.bmp]

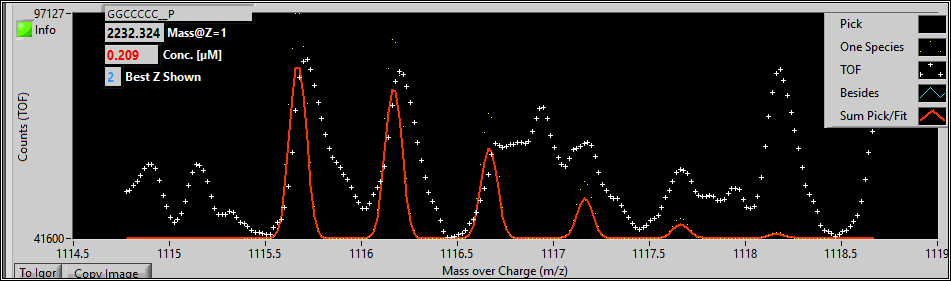

Supplement: Supplementary file 3 — Supplementary Data 1, 2 and 3 [file 41467_2025_60359_MOESM3_ESM.zip › Supplementary Data/Supplementary Data 3/GC-Val-SI/000064_GGCCCCC__P.bmp]

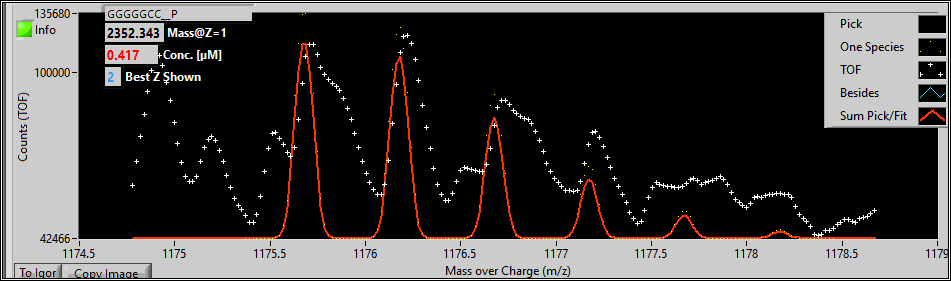

Supplement: Supplementary file 3 — Supplementary Data 1, 2 and 3 [file 41467_2025_60359_MOESM3_ESM.zip › Supplementary Data/Supplementary Data 3/GC-Val-SI/000058_GGGGGCC__P.bmp]

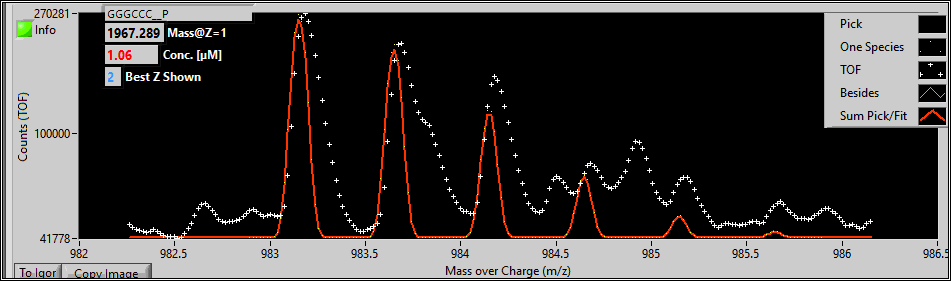

Supplement: Supplementary file 3 — Supplementary Data 1, 2 and 3 [file 41467_2025_60359_MOESM3_ESM.zip › Supplementary Data/Supplementary Data 3/GC-Val-SI/000046_GGGCCC__P.bmp]

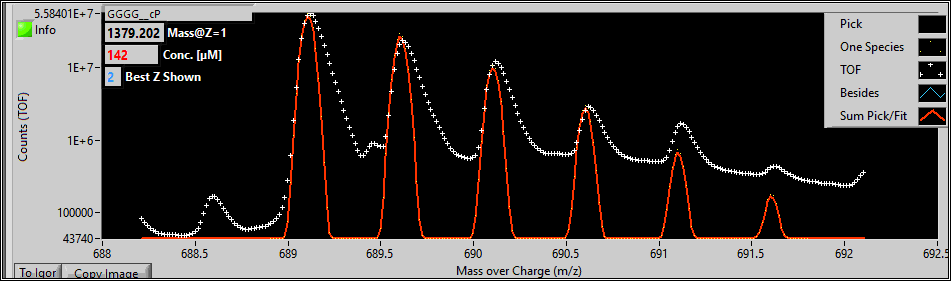

Supplement: Supplementary file 3 — Supplementary Data 1, 2 and 3 [file 41467_2025_60359_MOESM3_ESM.zip › Supplementary Data/Supplementary Data 3/GC-Val-SI/000019_GGGG__cP.bmp]

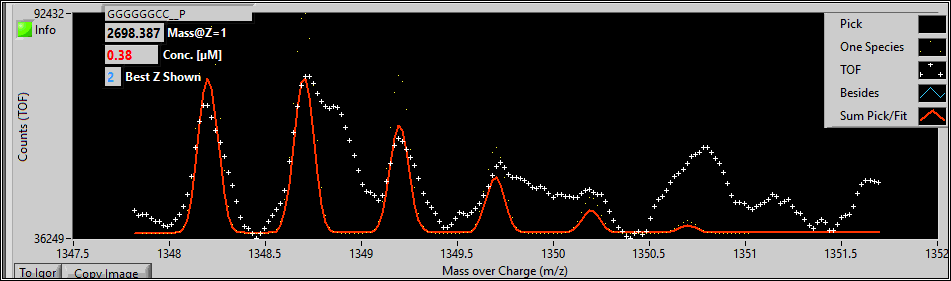

Supplement: Supplementary file 3 — Supplementary Data 1, 2 and 3 [file 41467_2025_60359_MOESM3_ESM.zip › Supplementary Data/Supplementary Data 3/GC-Val-SI/000074_GGGGGGCC__P.bmp]

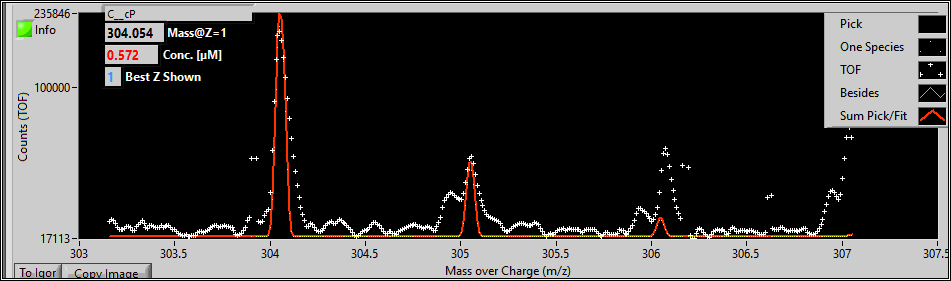

Supplement: Supplementary file 3 — Supplementary Data 1, 2 and 3 [file 41467_2025_60359_MOESM3_ESM.zip › Supplementary Data/Supplementary Data 3/GC-Val-SI/000003_C__cP.bmp]

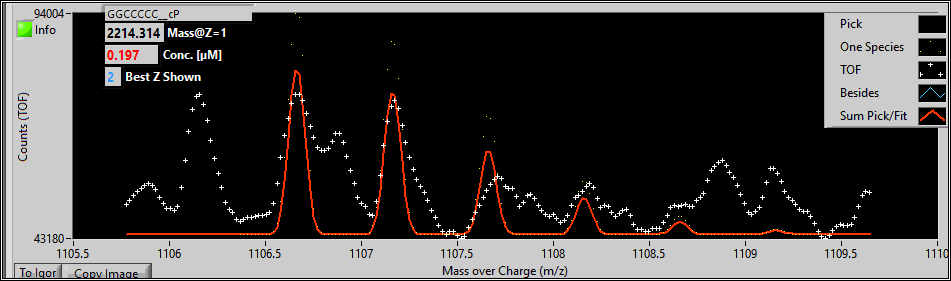

Supplement: Supplementary file 3 — Supplementary Data 1, 2 and 3 [file 41467_2025_60359_MOESM3_ESM.zip › Supplementary Data/Supplementary Data 3/GC-Val-SI/000065_GGCCCCC__cP.bmp]

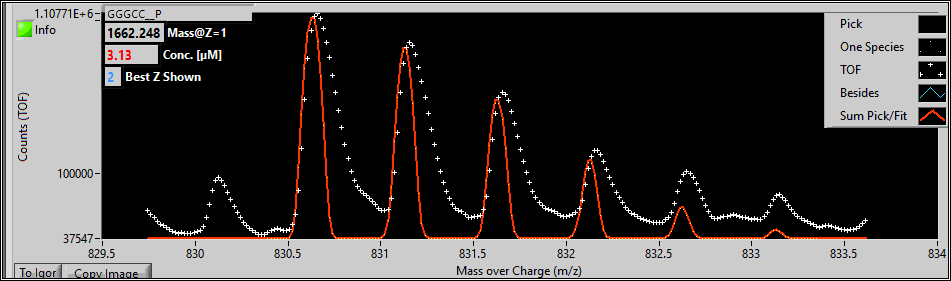

Supplement: Supplementary file 3 — Supplementary Data 1, 2 and 3 [file 41467_2025_60359_MOESM3_ESM.zip › Supplementary Data/Supplementary Data 3/GC-Val-SI/000032_GGGCC__P.bmp]

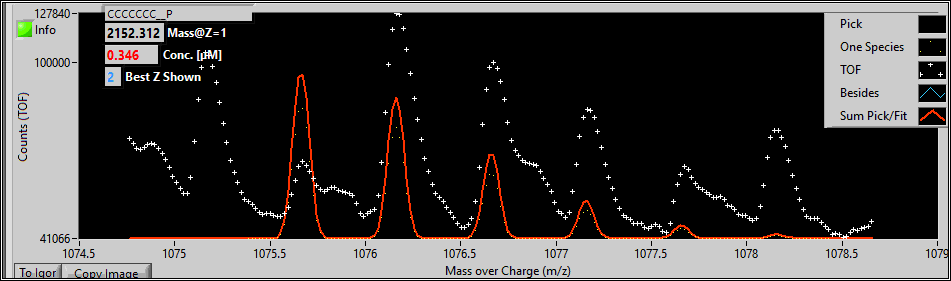

Supplement: Supplementary file 3 — Supplementary Data 1, 2 and 3 [file 41467_2025_60359_MOESM3_ESM.zip › Supplementary Data/Supplementary Data 3/GC-Val-SI/000068_CCCCCCC__P.bmp]

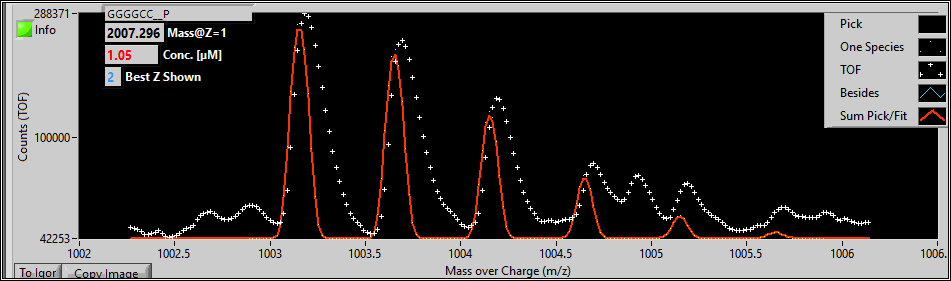

Supplement: Supplementary file 3 — Supplementary Data 1, 2 and 3 [file 41467_2025_60359_MOESM3_ESM.zip › Supplementary Data/Supplementary Data 3/GC-Val-SI/000044_GGGGCC__P.bmp]

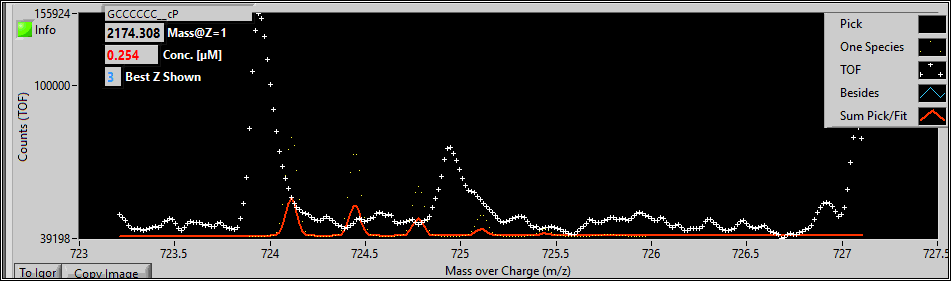

Supplement: Supplementary file 3 — Supplementary Data 1, 2 and 3 [file 41467_2025_60359_MOESM3_ESM.zip › Supplementary Data/Supplementary Data 3/GC-Val-SI/000067_GCCCCCC__cP.bmp]

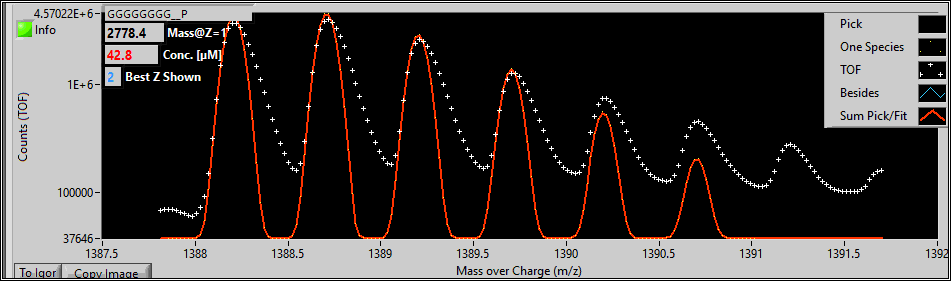

Supplement: Supplementary file 3 — Supplementary Data 1, 2 and 3 [file 41467_2025_60359_MOESM3_ESM.zip › Supplementary Data/Supplementary Data 3/GC-Val-SI/000070_GGGGGGGG__P.bmp]

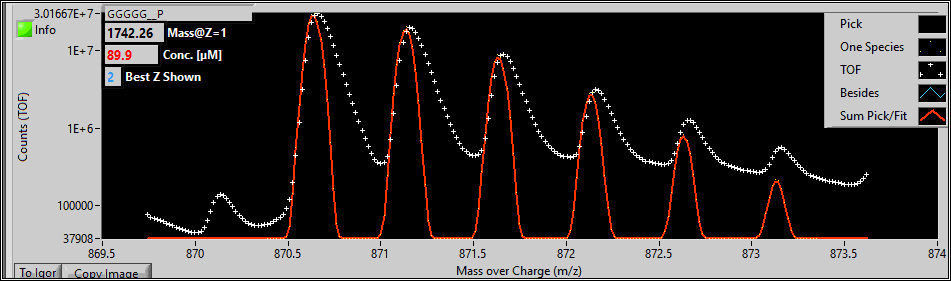

Supplement: Supplementary file 3 — Supplementary Data 1, 2 and 3 [file 41467_2025_60359_MOESM3_ESM.zip › Supplementary Data/Supplementary Data 3/GC-Val-SI/000028_GGGGG__P.bmp]

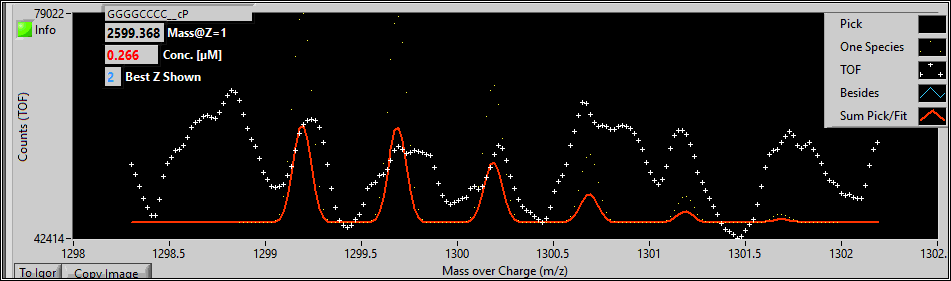

Supplement: Supplementary file 3 — Supplementary Data 1, 2 and 3 [file 41467_2025_60359_MOESM3_ESM.zip › Supplementary Data/Supplementary Data 3/GC-Val-SI/000079_GGGGCCCC__cP.bmp]

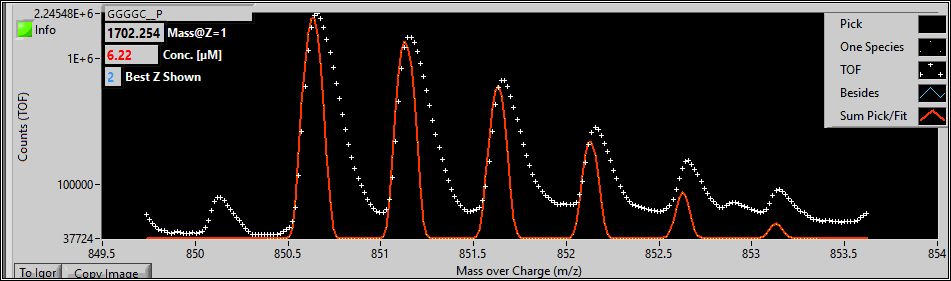

Supplement: Supplementary file 3 — Supplementary Data 1, 2 and 3 [file 41467_2025_60359_MOESM3_ESM.zip › Supplementary Data/Supplementary Data 3/GC-Val-SI/000030_GGGGC__P.bmp]

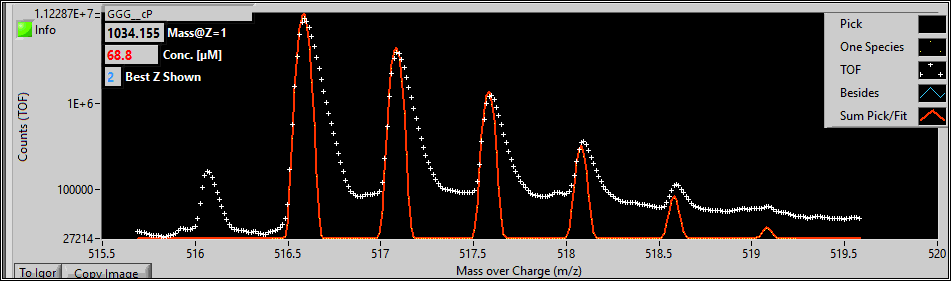

Supplement: Supplementary file 3 — Supplementary Data 1, 2 and 3 [file 41467_2025_60359_MOESM3_ESM.zip › Supplementary Data/Supplementary Data 3/GC-Val-SI/000011_GGG__cP.bmp]

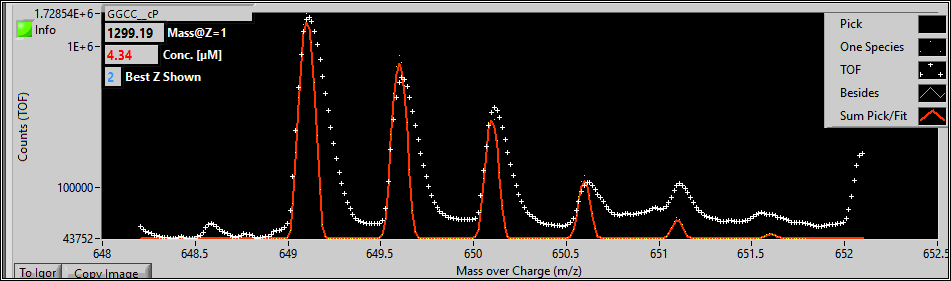

Supplement: Supplementary file 3 — Supplementary Data 1, 2 and 3 [file 41467_2025_60359_MOESM3_ESM.zip › Supplementary Data/Supplementary Data 3/GC-Val-SI/000023_GGCC__cP.bmp]

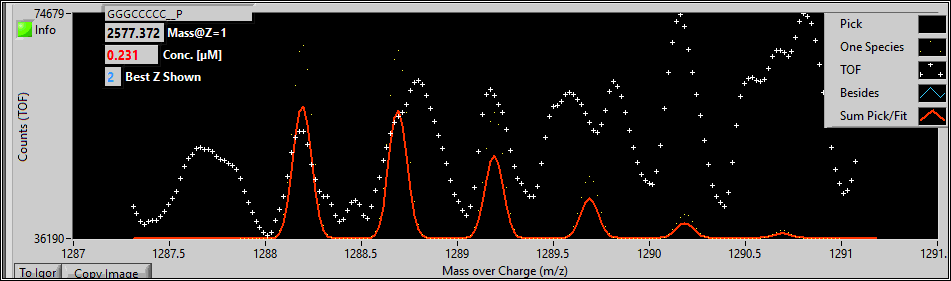

Supplement: Supplementary file 3 — Supplementary Data 1, 2 and 3 [file 41467_2025_60359_MOESM3_ESM.zip › Supplementary Data/Supplementary Data 3/GC-Val-SI/000080_GGGCCCCC__P.bmp]

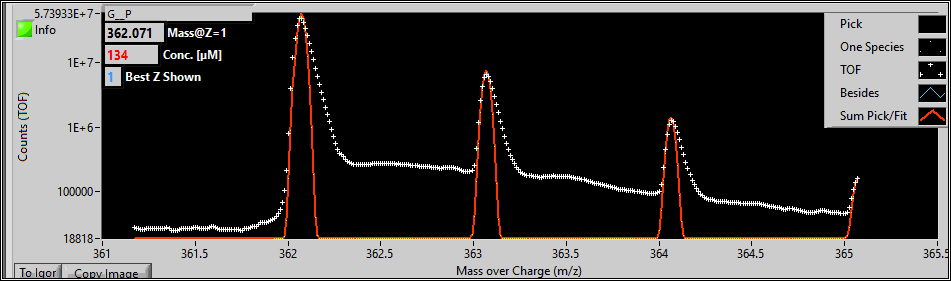

Supplement: Supplementary file 3 — Supplementary Data 1, 2 and 3 [file 41467_2025_60359_MOESM3_ESM.zip › Supplementary Data/Supplementary Data 3/GC-Val-SI/000000_G__P.bmp]

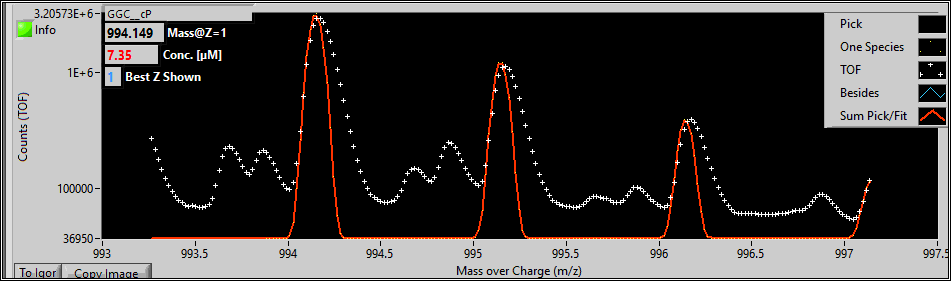

Supplement: Supplementary file 3 — Supplementary Data 1, 2 and 3 [file 41467_2025_60359_MOESM3_ESM.zip › Supplementary Data/Supplementary Data 3/GC-Val-SI/000013_GGC__cP.bmp]

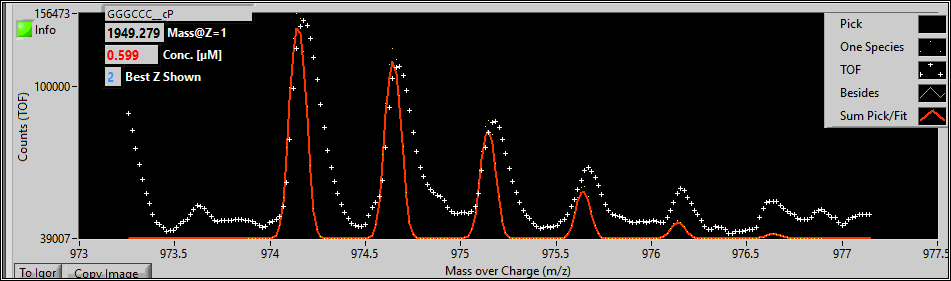

Supplement: Supplementary file 3 — Supplementary Data 1, 2 and 3 [file 41467_2025_60359_MOESM3_ESM.zip › Supplementary Data/Supplementary Data 3/GC-Val-SI/000047_GGGCCC__cP.bmp]

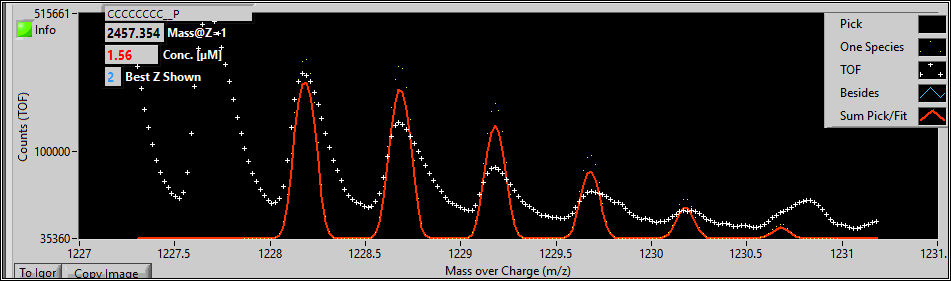

Supplement: Supplementary file 3 — Supplementary Data 1, 2 and 3 [file 41467_2025_60359_MOESM3_ESM.zip › Supplementary Data/Supplementary Data 3/GC-Val-SI/000086_CCCCCCCC__P.bmp]

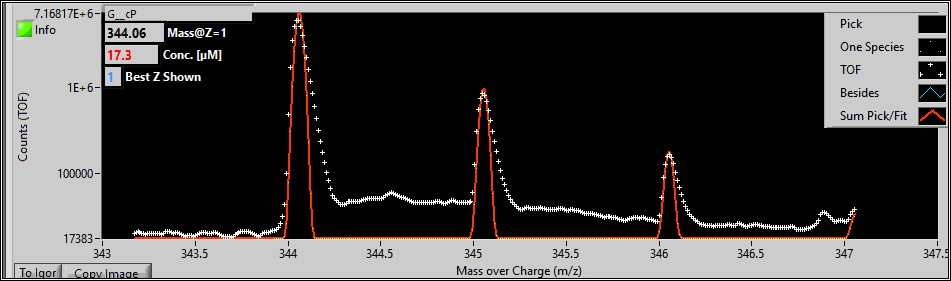

Supplement: Supplementary file 3 — Supplementary Data 1, 2 and 3 [file 41467_2025_60359_MOESM3_ESM.zip › Supplementary Data/Supplementary Data 3/GC-Val-SI/000001_G__cP.bmp]

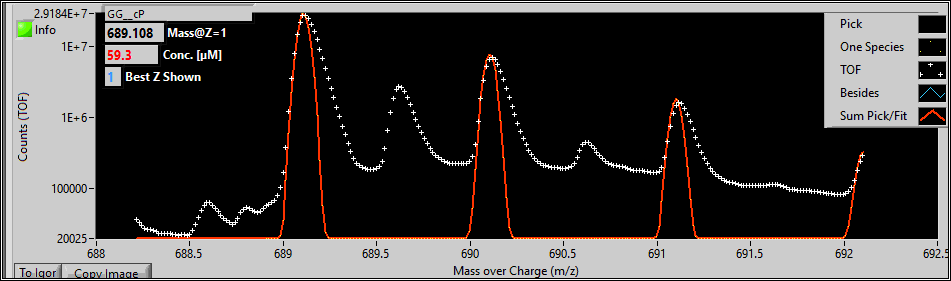

Supplement: Supplementary file 3 — Supplementary Data 1, 2 and 3 [file 41467_2025_60359_MOESM3_ESM.zip › Supplementary Data/Supplementary Data 3/GC-Val-SI/000005_GG__cP.bmp]

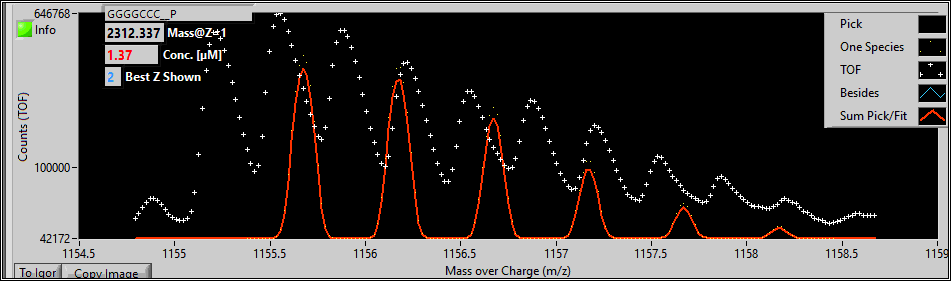

Supplement: Supplementary file 3 — Supplementary Data 1, 2 and 3 [file 41467_2025_60359_MOESM3_ESM.zip › Supplementary Data/Supplementary Data 3/GC-Val-SI/000060_GGGGCCC__P.bmp]

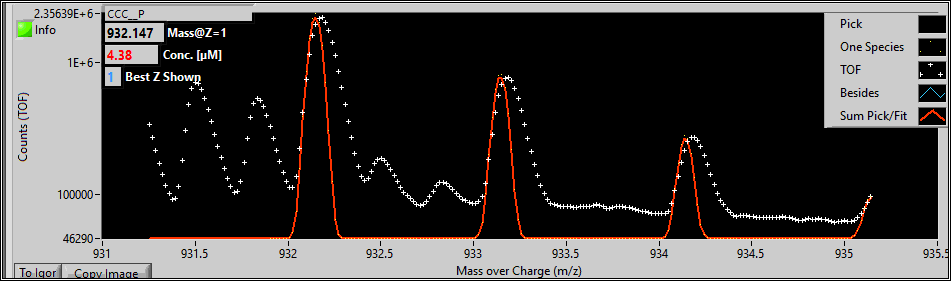

Supplement: Supplementary file 3 — Supplementary Data 1, 2 and 3 [file 41467_2025_60359_MOESM3_ESM.zip › Supplementary Data/Supplementary Data 3/GC-Val-SI/000016_CCC__P.bmp]

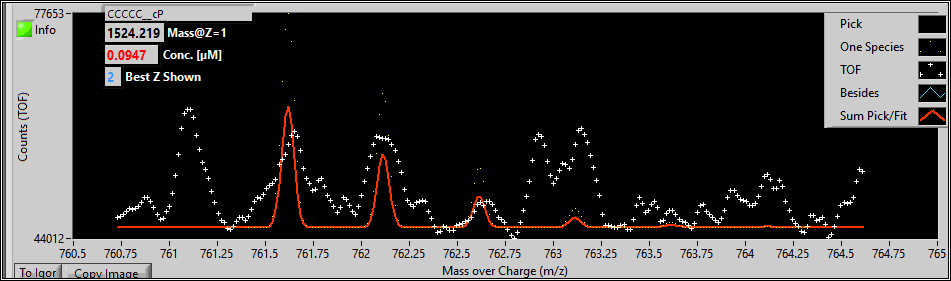

Supplement: Supplementary file 3 — Supplementary Data 1, 2 and 3 [file 41467_2025_60359_MOESM3_ESM.zip › Supplementary Data/Supplementary Data 3/GC-Val-SI/000039_CCCCC__cP.bmp]

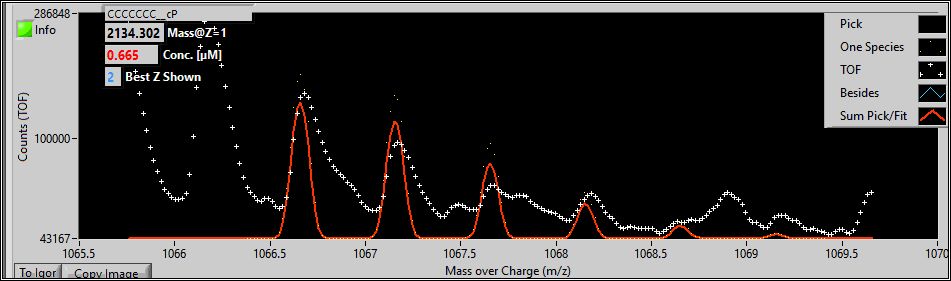

Supplement: Supplementary file 3 — Supplementary Data 1, 2 and 3 [file 41467_2025_60359_MOESM3_ESM.zip › Supplementary Data/Supplementary Data 3/GC-Val-SI/000069_CCCCCCC__cP.bmp]

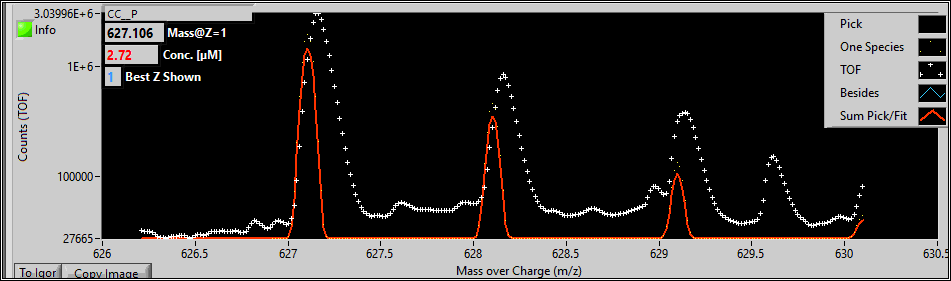

Supplement: Supplementary file 3 — Supplementary Data 1, 2 and 3 [file 41467_2025_60359_MOESM3_ESM.zip › Supplementary Data/Supplementary Data 3/GC-Val-SI/000008_CC__P.bmp]

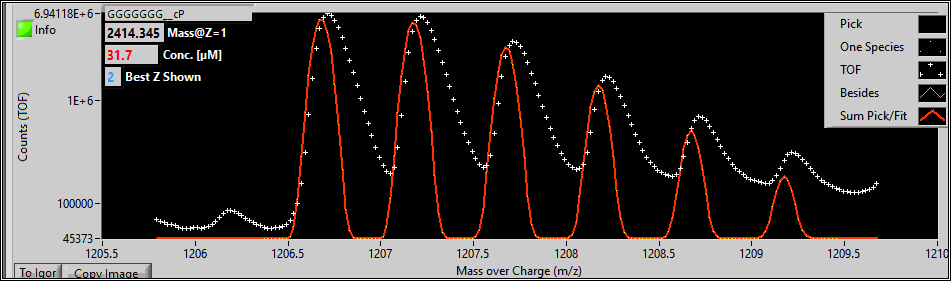

Supplement: Supplementary file 3 — Supplementary Data 1, 2 and 3 [file 41467_2025_60359_MOESM3_ESM.zip › Supplementary Data/Supplementary Data 3/GC-Val-SI/000055_GGGGGGG__cP.bmp]

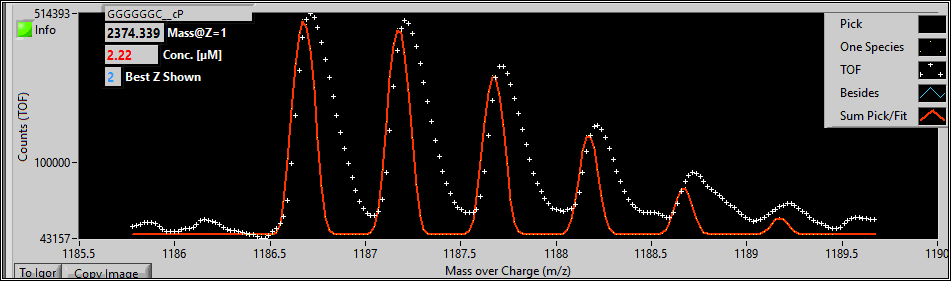

Supplement: Supplementary file 3 — Supplementary Data 1, 2 and 3 [file 41467_2025_60359_MOESM3_ESM.zip › Supplementary Data/Supplementary Data 3/GC-Val-SI/000057_GGGGGGC__cP.bmp]

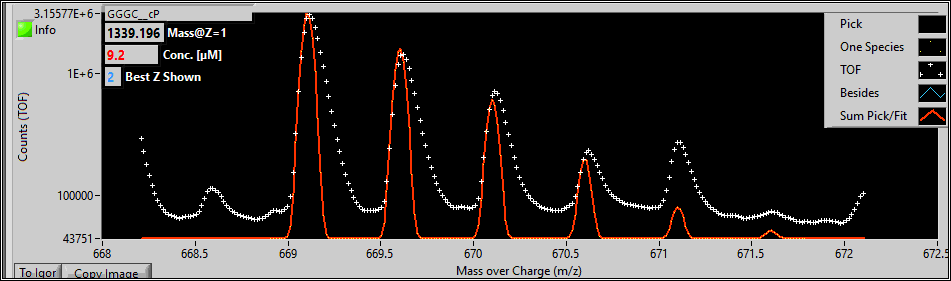

Supplement: Supplementary file 3 — Supplementary Data 1, 2 and 3 [file 41467_2025_60359_MOESM3_ESM.zip › Supplementary Data/Supplementary Data 3/GC-Val-SI/000021_GGGC__cP.bmp]

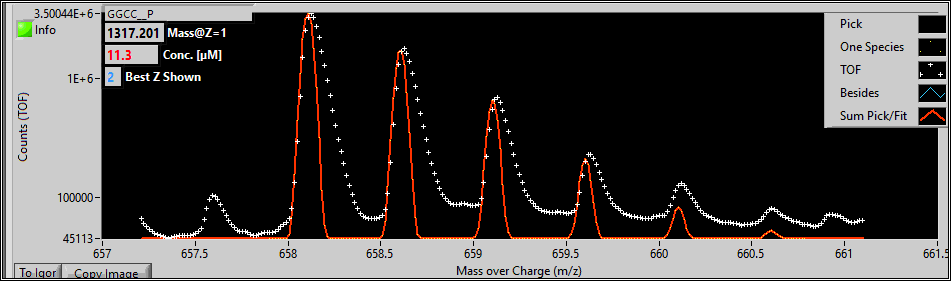

Supplement: Supplementary file 3 — Supplementary Data 1, 2 and 3 [file 41467_2025_60359_MOESM3_ESM.zip › Supplementary Data/Supplementary Data 3/GC-Val-SI/000022_GGCC__P.bmp]

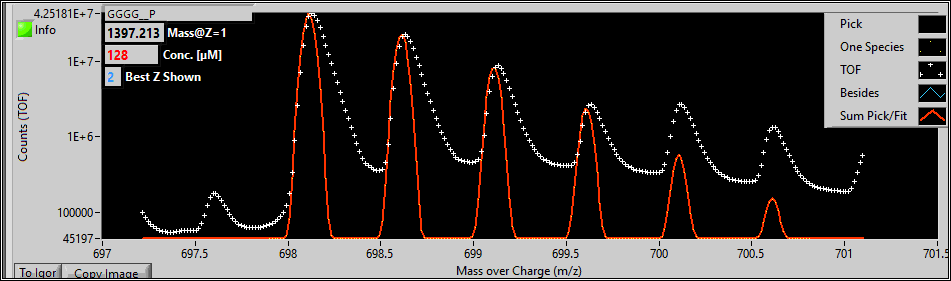

Supplement: Supplementary file 3 — Supplementary Data 1, 2 and 3 [file 41467_2025_60359_MOESM3_ESM.zip › Supplementary Data/Supplementary Data 3/GC-Val-SI/000018_GGGG__P.bmp]

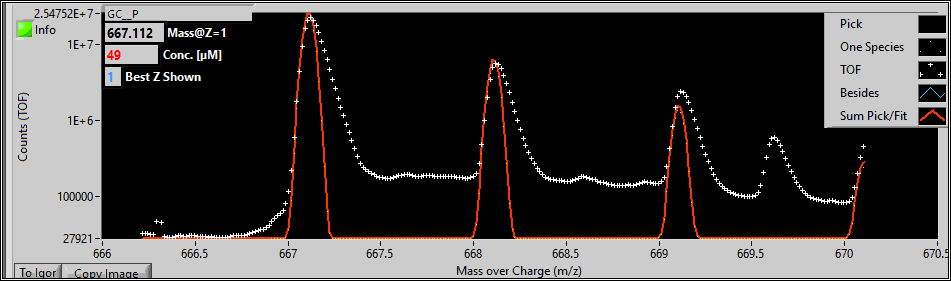

Supplement: Supplementary file 3 — Supplementary Data 1, 2 and 3 [file 41467_2025_60359_MOESM3_ESM.zip › Supplementary Data/Supplementary Data 3/GC-Val-SI/000006_GC__P.bmp]

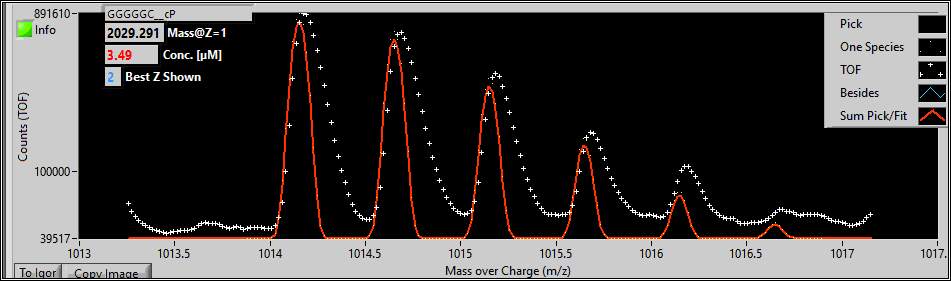

Supplement: Supplementary file 3 — Supplementary Data 1, 2 and 3 [file 41467_2025_60359_MOESM3_ESM.zip › Supplementary Data/Supplementary Data 3/GC-Val-SI/000043_GGGGGC__cP.bmp]

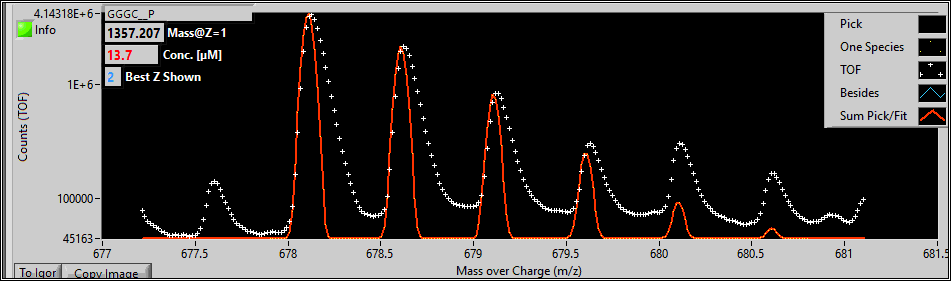

Supplement: Supplementary file 3 — Supplementary Data 1, 2 and 3 [file 41467_2025_60359_MOESM3_ESM.zip › Supplementary Data/Supplementary Data 3/GC-Val-SI/000020_GGGC__P.bmp]

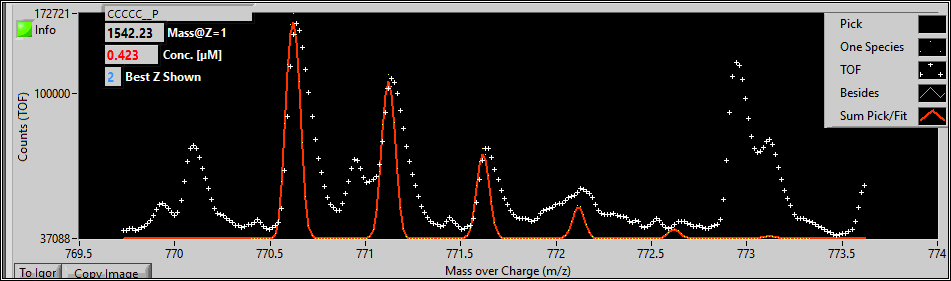

Supplement: Supplementary file 3 — Supplementary Data 1, 2 and 3 [file 41467_2025_60359_MOESM3_ESM.zip › Supplementary Data/Supplementary Data 3/GC-Val-SI/000038_CCCCC__P.bmp]

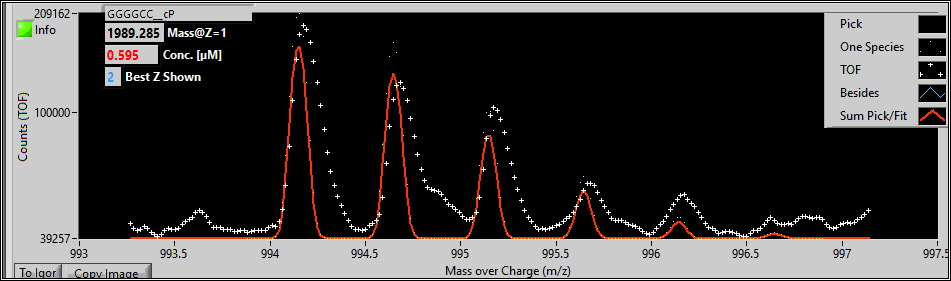

Supplement: Supplementary file 3 — Supplementary Data 1, 2 and 3 [file 41467_2025_60359_MOESM3_ESM.zip › Supplementary Data/Supplementary Data 3/GC-Val-SI/000045_GGGGCC__cP.bmp]

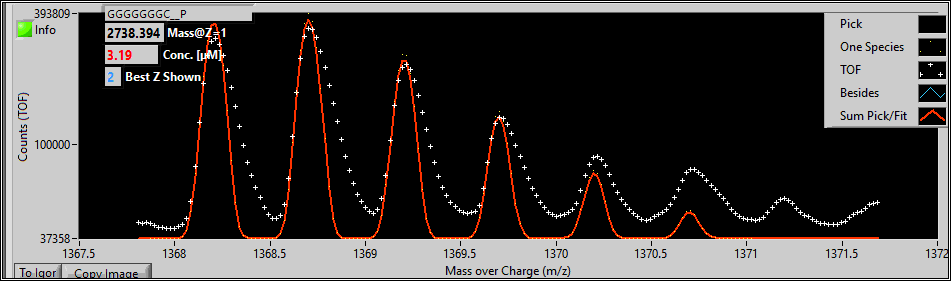

Supplement: Supplementary file 3 — Supplementary Data 1, 2 and 3 [file 41467_2025_60359_MOESM3_ESM.zip › Supplementary Data/Supplementary Data 3/GC-Val-SI/000072_GGGGGGGC__P.bmp]

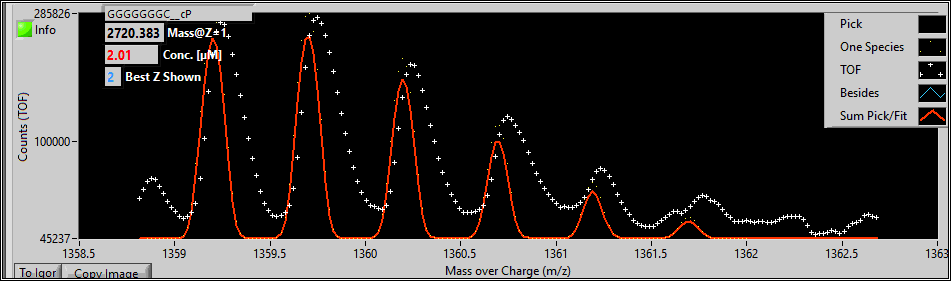

Supplement: Supplementary file 3 — Supplementary Data 1, 2 and 3 [file 41467_2025_60359_MOESM3_ESM.zip › Supplementary Data/Supplementary Data 3/GC-Val-SI/000073_GGGGGGGC__cP.bmp]

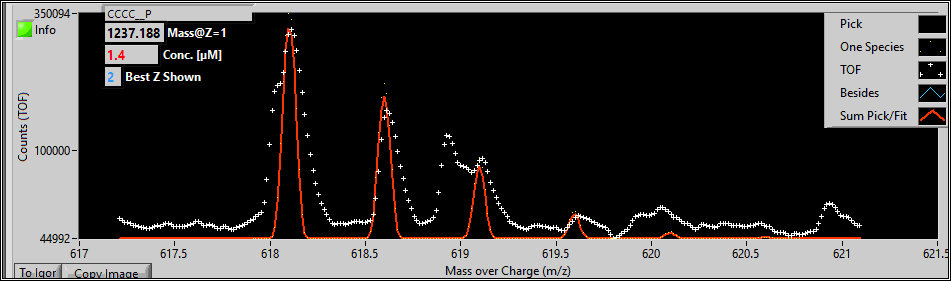

Supplement: Supplementary file 3 — Supplementary Data 1, 2 and 3 [file 41467_2025_60359_MOESM3_ESM.zip › Supplementary Data/Supplementary Data 3/GC-Val-SI/000026_CCCC__P.bmp]

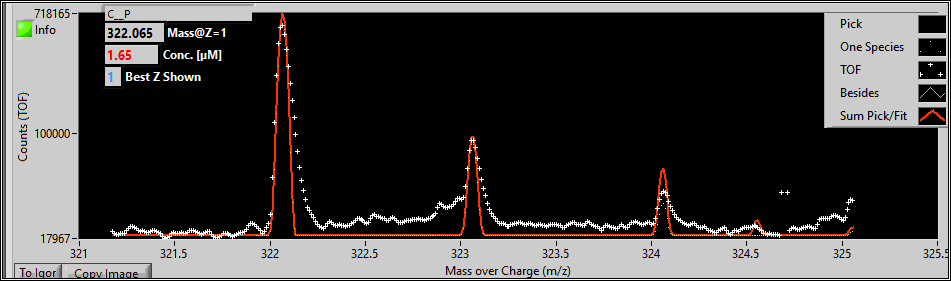

Supplement: Supplementary file 3 — Supplementary Data 1, 2 and 3 [file 41467_2025_60359_MOESM3_ESM.zip › Supplementary Data/Supplementary Data 3/GC-Val-SI/000002_C__P.bmp]

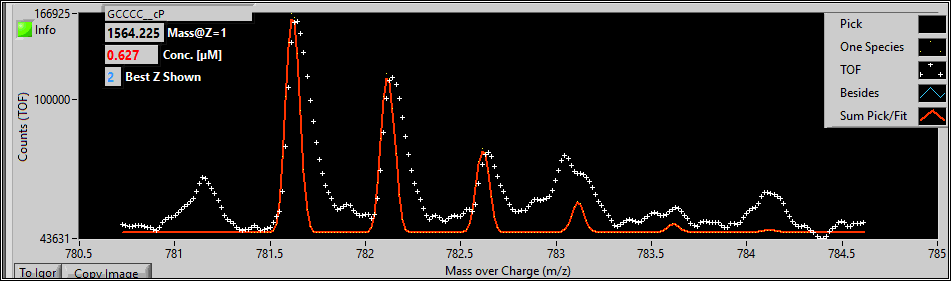

Supplement: Supplementary file 3 — Supplementary Data 1, 2 and 3 [file 41467_2025_60359_MOESM3_ESM.zip › Supplementary Data/Supplementary Data 3/GC-Val-SI/000037_GCCCC__cP.bmp]

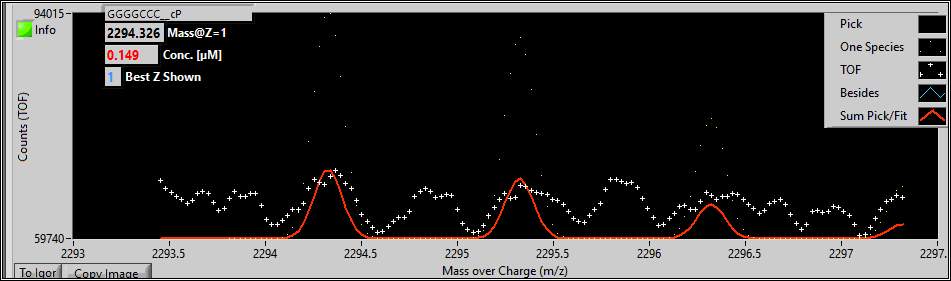

Supplement: Supplementary file 3 — Supplementary Data 1, 2 and 3 [file 41467_2025_60359_MOESM3_ESM.zip › Supplementary Data/Supplementary Data 3/GC-Val-SI/000061_GGGGCCC__cP.bmp]

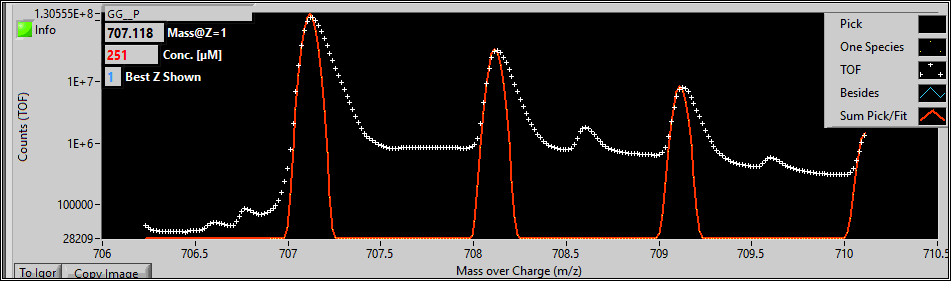

Supplement: Supplementary file 3 — Supplementary Data 1, 2 and 3 [file 41467_2025_60359_MOESM3_ESM.zip › Supplementary Data/Supplementary Data 3/GC-Val-SI/000004_GG__P.bmp]

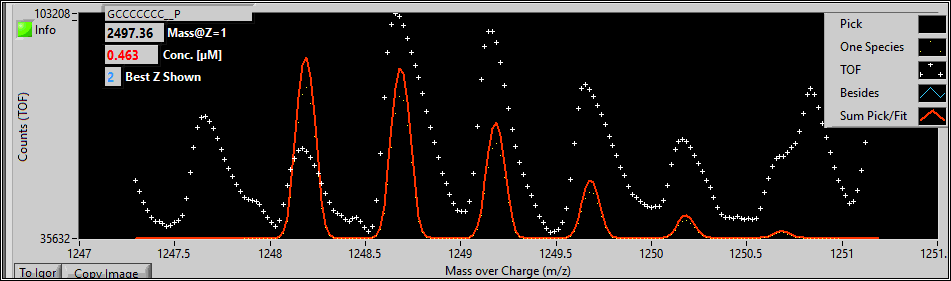

Supplement: Supplementary file 3 — Supplementary Data 1, 2 and 3 [file 41467_2025_60359_MOESM3_ESM.zip › Supplementary Data/Supplementary Data 3/GC-Val-SI/000084_GCCCCCCC__P.bmp]

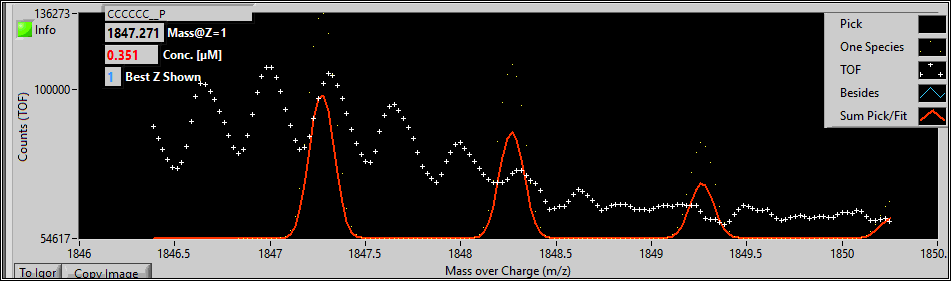

Supplement: Supplementary file 3 — Supplementary Data 1, 2 and 3 [file 41467_2025_60359_MOESM3_ESM.zip › Supplementary Data/Supplementary Data 3/GC-Val-SI/000052_CCCCCC__P.bmp]

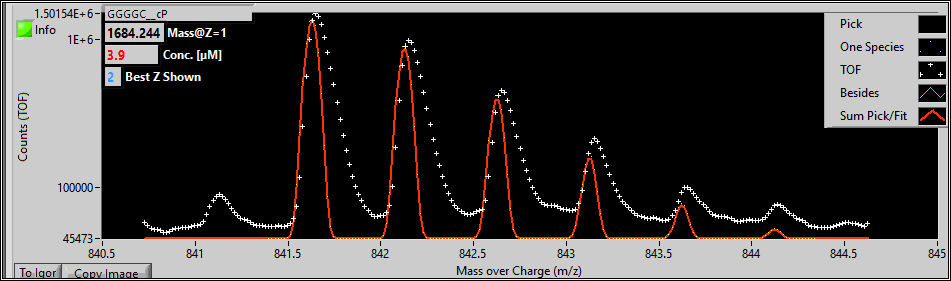

Supplement: Supplementary file 3 — Supplementary Data 1, 2 and 3 [file 41467_2025_60359_MOESM3_ESM.zip › Supplementary Data/Supplementary Data 3/GC-Val-SI/000031_GGGGC__cP.bmp]

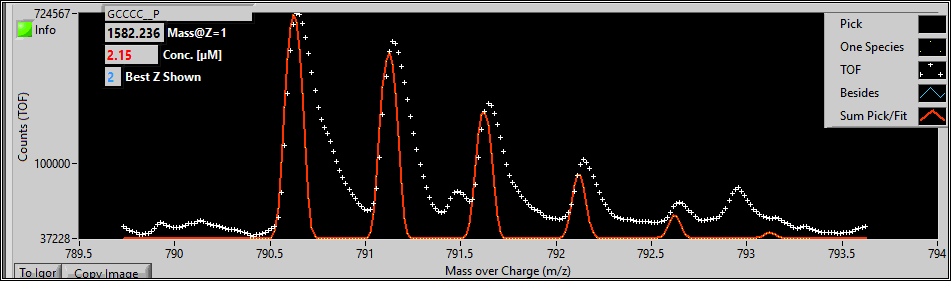

Supplement: Supplementary file 3 — Supplementary Data 1, 2 and 3 [file 41467_2025_60359_MOESM3_ESM.zip › Supplementary Data/Supplementary Data 3/GC-Val-SI/000036_GCCCC__P.bmp]

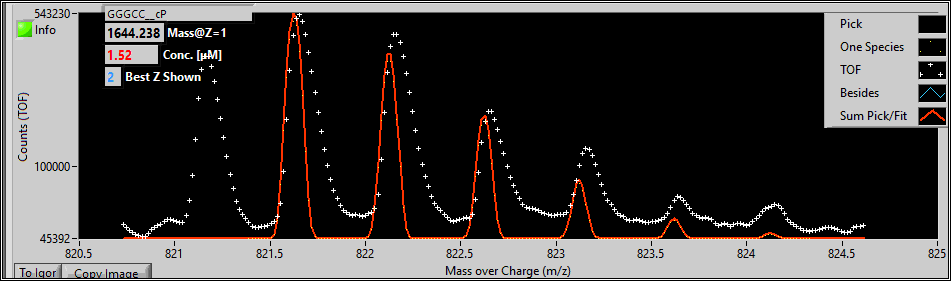

Supplement: Supplementary file 3 — Supplementary Data 1, 2 and 3 [file 41467_2025_60359_MOESM3_ESM.zip › Supplementary Data/Supplementary Data 3/GC-Val-SI/000033_GGGCC__cP.bmp]

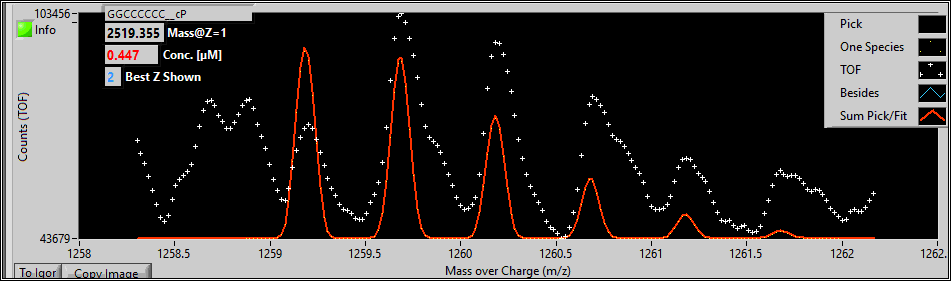

Supplement: Supplementary file 3 — Supplementary Data 1, 2 and 3 [file 41467_2025_60359_MOESM3_ESM.zip › Supplementary Data/Supplementary Data 3/GC-Val-SI/000083_GGCCCCCC__cP.bmp]

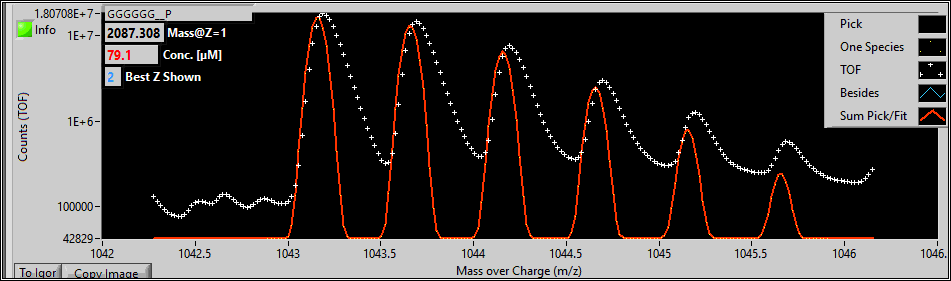

Supplement: Supplementary file 3 — Supplementary Data 1, 2 and 3 [file 41467_2025_60359_MOESM3_ESM.zip › Supplementary Data/Supplementary Data 3/GC-Val-SI/000040_GGGGGG__P.bmp]

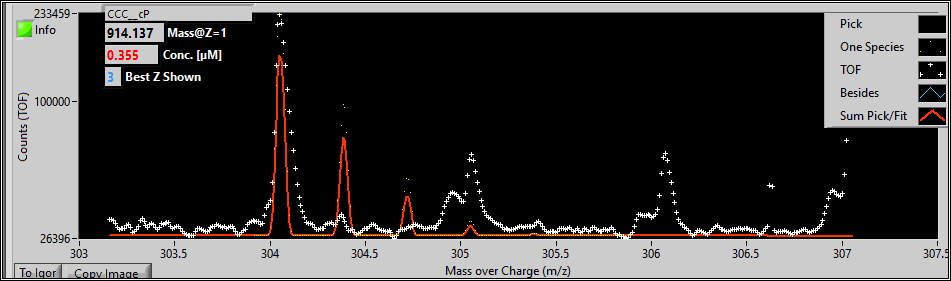

Supplement: Supplementary file 3 — Supplementary Data 1, 2 and 3 [file 41467_2025_60359_MOESM3_ESM.zip › Supplementary Data/Supplementary Data 3/GC-Val-SI/000017_CCC__cP.bmp]

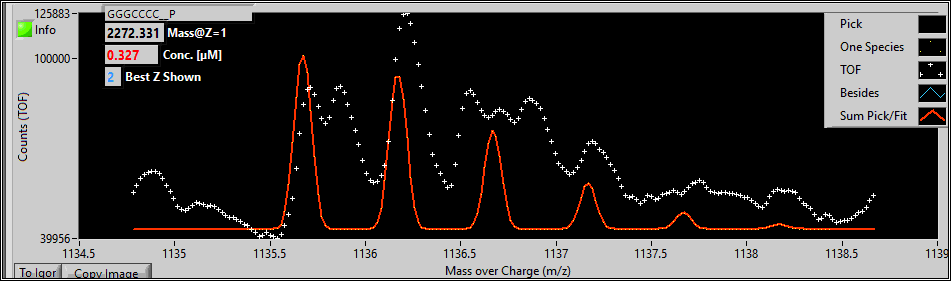

Supplement: Supplementary file 3 — Supplementary Data 1, 2 and 3 [file 41467_2025_60359_MOESM3_ESM.zip › Supplementary Data/Supplementary Data 3/GC-Val-SI/000062_GGGCCCC__P.bmp]

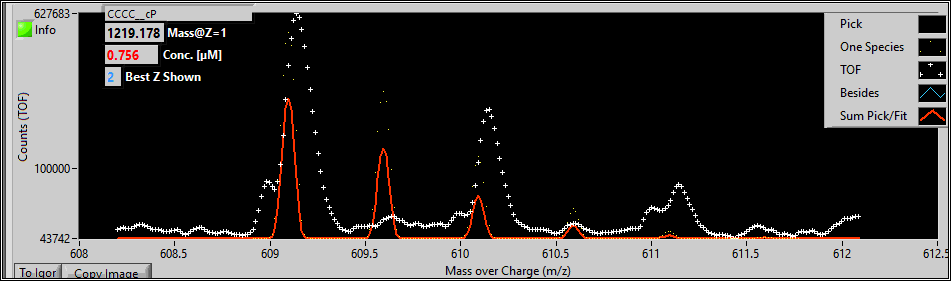

Supplement: Supplementary file 3 — Supplementary Data 1, 2 and 3 [file 41467_2025_60359_MOESM3_ESM.zip › Supplementary Data/Supplementary Data 3/GC-Val-SI/000027_CCCC__cP.bmp]

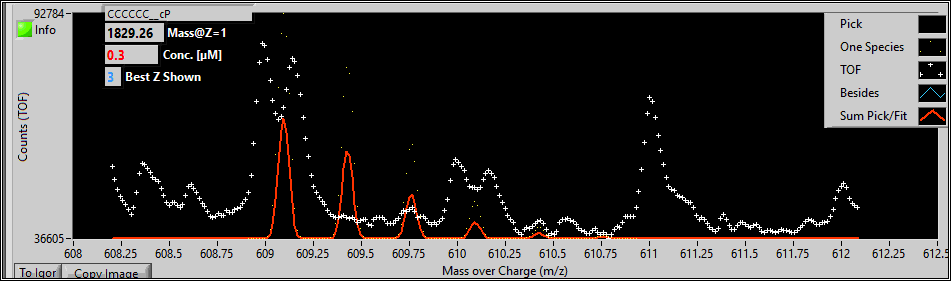

Supplement: Supplementary file 3 — Supplementary Data 1, 2 and 3 [file 41467_2025_60359_MOESM3_ESM.zip › Supplementary Data/Supplementary Data 3/GC-Val-SI/000053_CCCCCC__cP.bmp]
