# Supplementary figures and images for: Amino acids catalyse RNA formation under ambient alkaline conditions (part 1 of 2)
Source: Nat Commun. 2025 Jun 4;16:5193. doi: 10.1038/s41467-025-60359-3 (PMC12137669; doi:10.1038/s41467-025-60359-3)

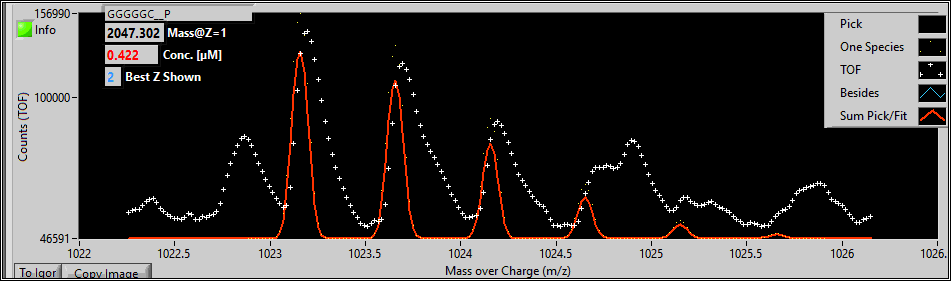

Supplement: Supplementary file 3 — Supplementary Data 1, 2 and 3 [file 41467_2025_60359_MOESM3_ESM.zip › Supplementary Data/Supplementary Data 3/GC-no aa-SI/000042_GGGGGC__P.bmp]

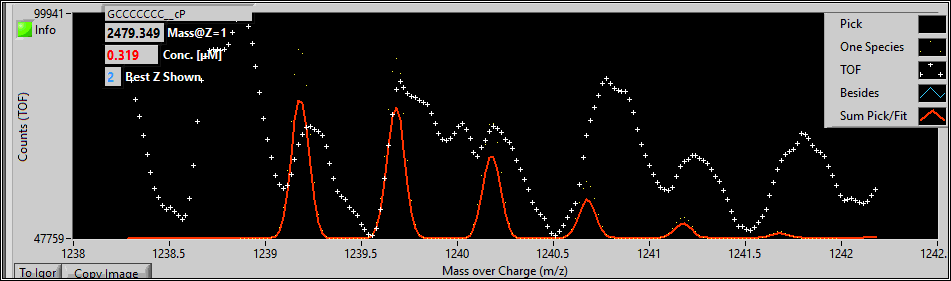

Supplement: Supplementary file 3 — Supplementary Data 1, 2 and 3 [file 41467_2025_60359_MOESM3_ESM.zip › Supplementary Data/Supplementary Data 3/GC-no aa-SI/000085_GCCCCCCC__cP.bmp]

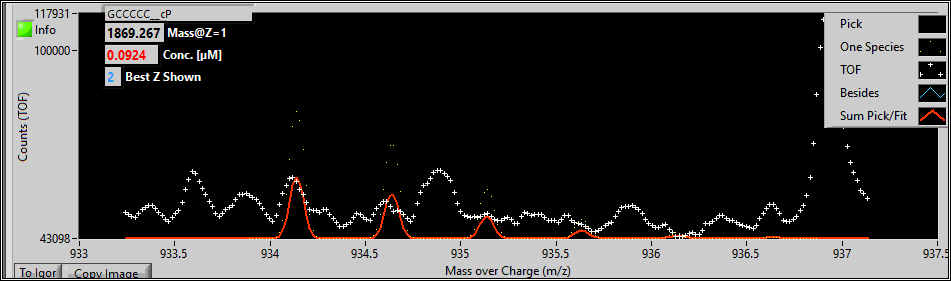

Supplement: Supplementary file 3 — Supplementary Data 1, 2 and 3 [file 41467_2025_60359_MOESM3_ESM.zip › Supplementary Data/Supplementary Data 3/GC-no aa-SI/000051_GCCCCC__cP.bmp]

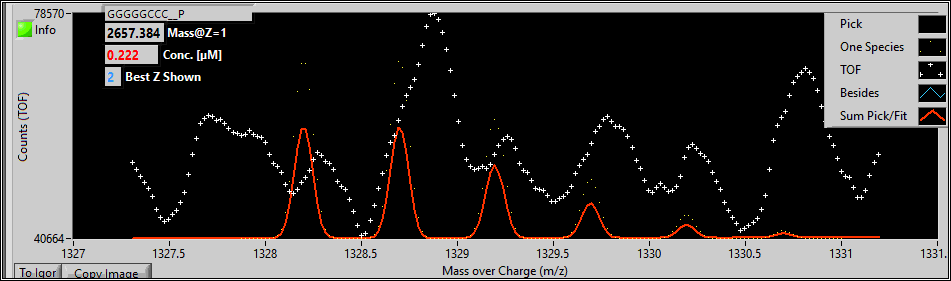

Supplement: Supplementary file 3 — Supplementary Data 1, 2 and 3 [file 41467_2025_60359_MOESM3_ESM.zip › Supplementary Data/Supplementary Data 3/GC-no aa-SI/000076_GGGGGCCC__P.bmp]

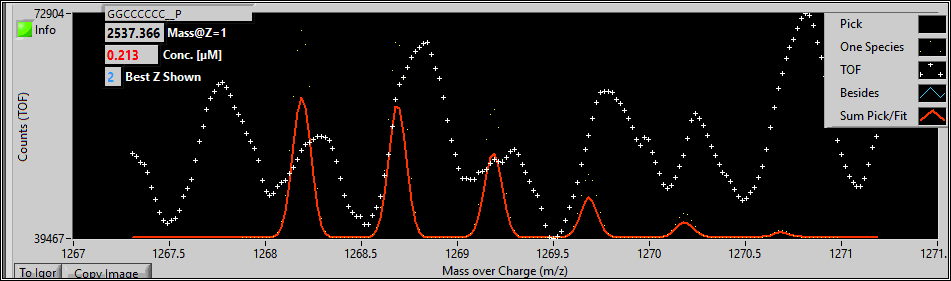

Supplement: Supplementary file 3 — Supplementary Data 1, 2 and 3 [file 41467_2025_60359_MOESM3_ESM.zip › Supplementary Data/Supplementary Data 3/GC-no aa-SI/000082_GGCCCCCC__P.bmp]

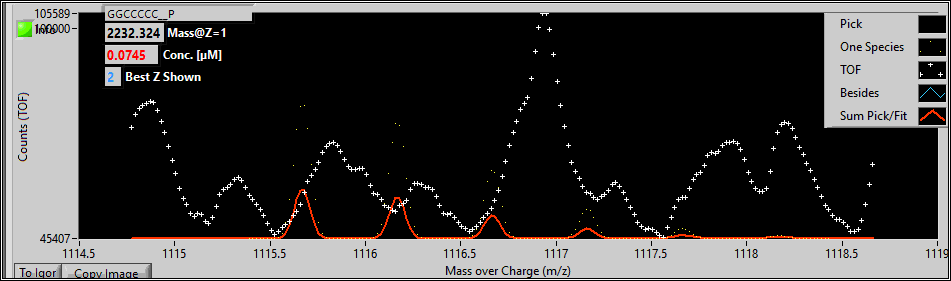

Supplement: Supplementary file 3 — Supplementary Data 1, 2 and 3 [file 41467_2025_60359_MOESM3_ESM.zip › Supplementary Data/Supplementary Data 3/GC-no aa-SI/000064_GGCCCCC__P.bmp]

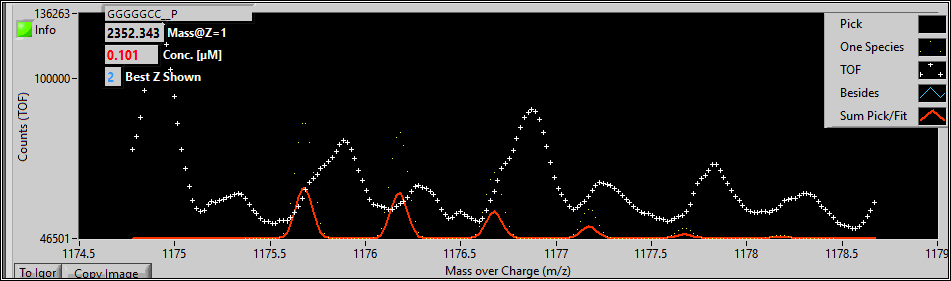

Supplement: Supplementary file 3 — Supplementary Data 1, 2 and 3 [file 41467_2025_60359_MOESM3_ESM.zip › Supplementary Data/Supplementary Data 3/GC-no aa-SI/000058_GGGGGCC__P.bmp]

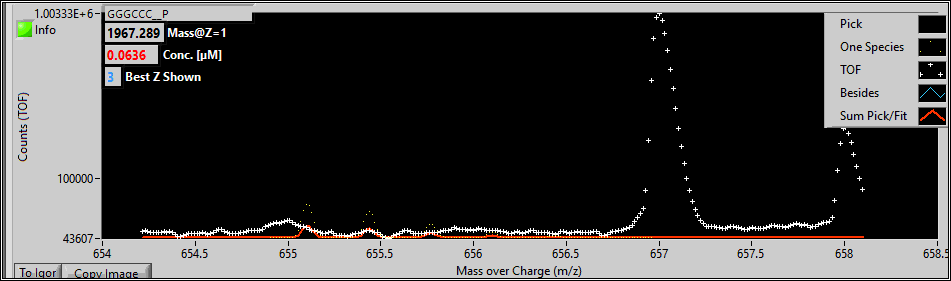

Supplement: Supplementary file 3 — Supplementary Data 1, 2 and 3 [file 41467_2025_60359_MOESM3_ESM.zip › Supplementary Data/Supplementary Data 3/GC-no aa-SI/000046_GGGCCC__P.bmp]

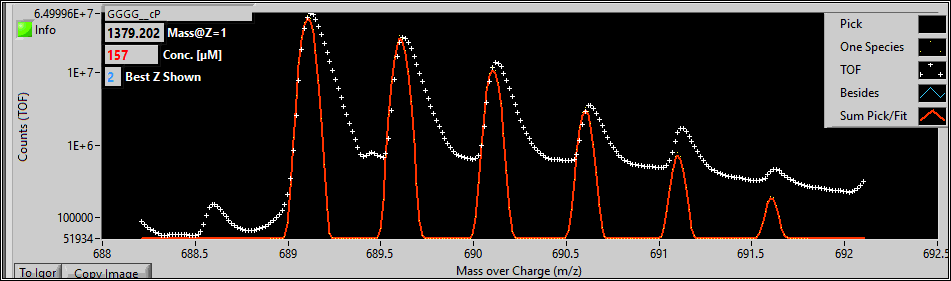

Supplement: Supplementary file 3 — Supplementary Data 1, 2 and 3 [file 41467_2025_60359_MOESM3_ESM.zip › Supplementary Data/Supplementary Data 3/GC-no aa-SI/000019_GGGG__cP.bmp]

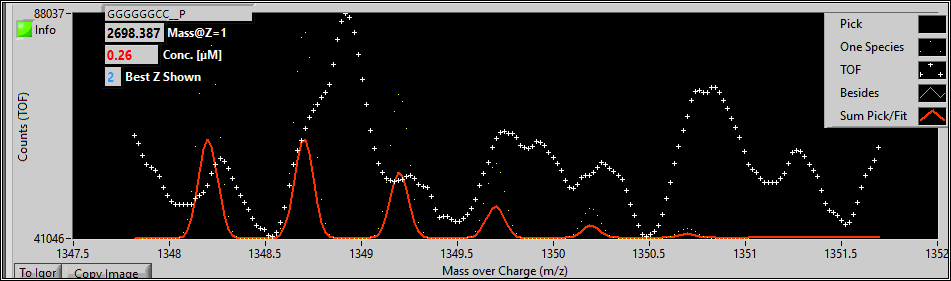

Supplement: Supplementary file 3 — Supplementary Data 1, 2 and 3 [file 41467_2025_60359_MOESM3_ESM.zip › Supplementary Data/Supplementary Data 3/GC-no aa-SI/000074_GGGGGGCC__P.bmp]

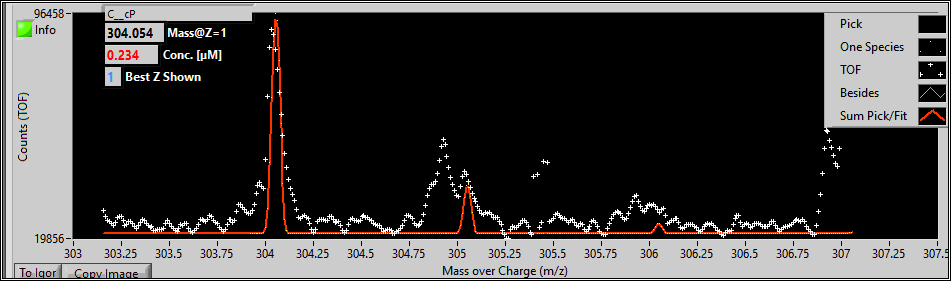

Supplement: Supplementary file 3 — Supplementary Data 1, 2 and 3 [file 41467_2025_60359_MOESM3_ESM.zip › Supplementary Data/Supplementary Data 3/GC-no aa-SI/000003_C__cP.bmp]

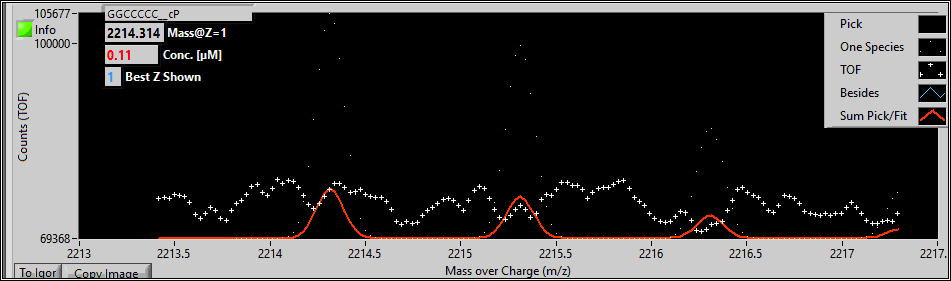

Supplement: Supplementary file 3 — Supplementary Data 1, 2 and 3 [file 41467_2025_60359_MOESM3_ESM.zip › Supplementary Data/Supplementary Data 3/GC-no aa-SI/000065_GGCCCCC__cP.bmp]

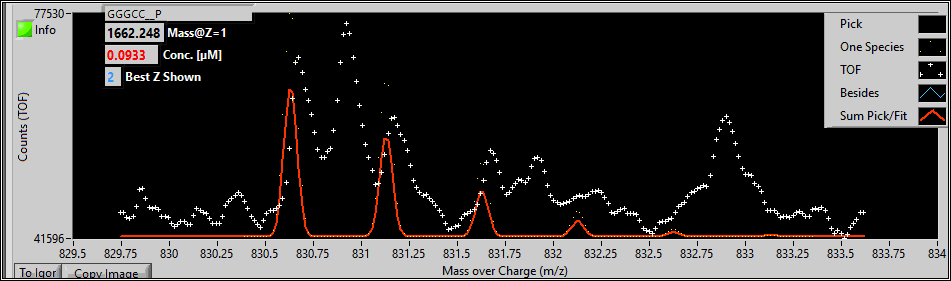

Supplement: Supplementary file 3 — Supplementary Data 1, 2 and 3 [file 41467_2025_60359_MOESM3_ESM.zip › Supplementary Data/Supplementary Data 3/GC-no aa-SI/000032_GGGCC__P.bmp]

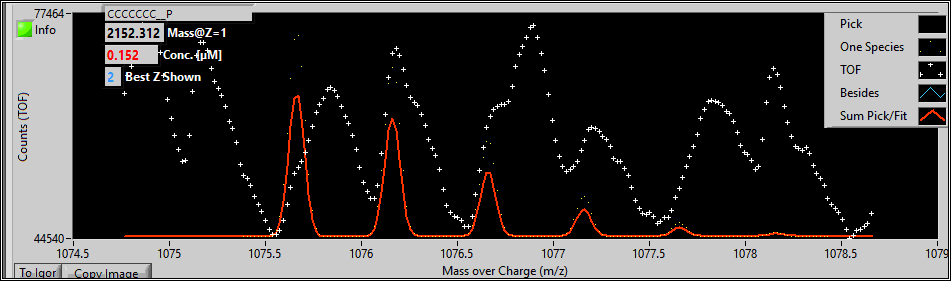

Supplement: Supplementary file 3 — Supplementary Data 1, 2 and 3 [file 41467_2025_60359_MOESM3_ESM.zip › Supplementary Data/Supplementary Data 3/GC-no aa-SI/000068_CCCCCCC__P.bmp]

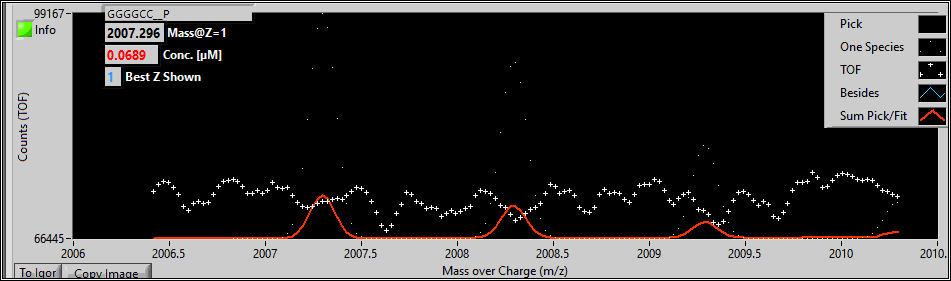

Supplement: Supplementary file 3 — Supplementary Data 1, 2 and 3 [file 41467_2025_60359_MOESM3_ESM.zip › Supplementary Data/Supplementary Data 3/GC-no aa-SI/000044_GGGGCC__P.bmp]

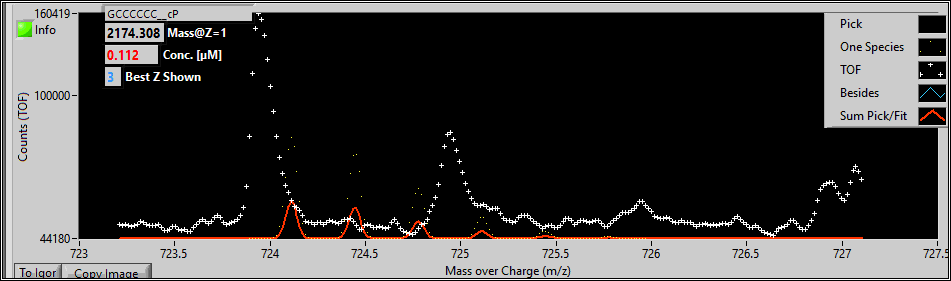

Supplement: Supplementary file 3 — Supplementary Data 1, 2 and 3 [file 41467_2025_60359_MOESM3_ESM.zip › Supplementary Data/Supplementary Data 3/GC-no aa-SI/000067_GCCCCCC__cP.bmp]

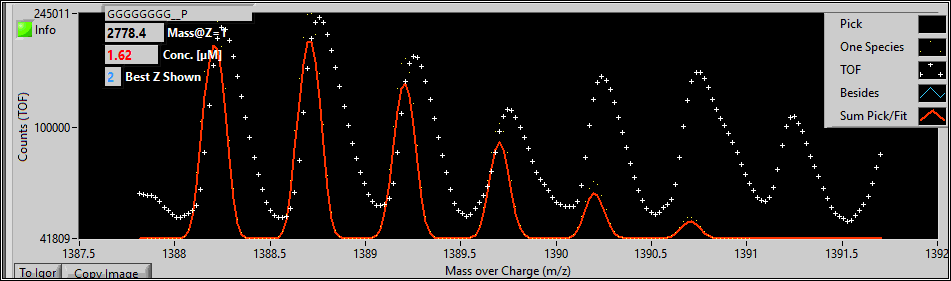

Supplement: Supplementary file 3 — Supplementary Data 1, 2 and 3 [file 41467_2025_60359_MOESM3_ESM.zip › Supplementary Data/Supplementary Data 3/GC-no aa-SI/000070_GGGGGGGG__P.bmp]

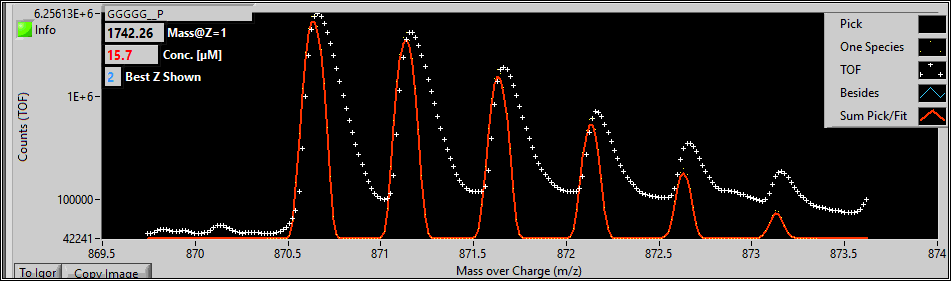

Supplement: Supplementary file 3 — Supplementary Data 1, 2 and 3 [file 41467_2025_60359_MOESM3_ESM.zip › Supplementary Data/Supplementary Data 3/GC-no aa-SI/000028_GGGGG__P.bmp]

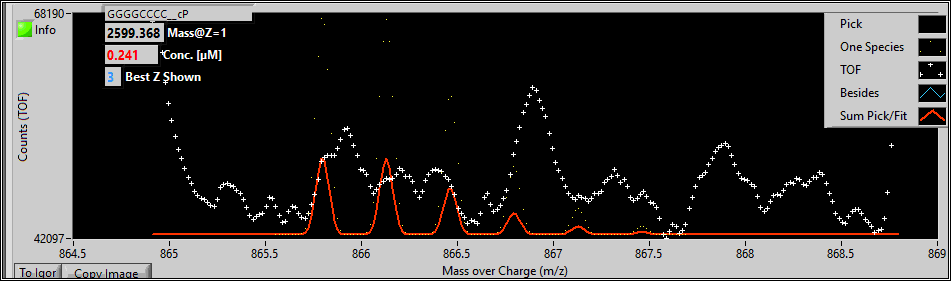

Supplement: Supplementary file 3 — Supplementary Data 1, 2 and 3 [file 41467_2025_60359_MOESM3_ESM.zip › Supplementary Data/Supplementary Data 3/GC-no aa-SI/000079_GGGGCCCC__cP.bmp]

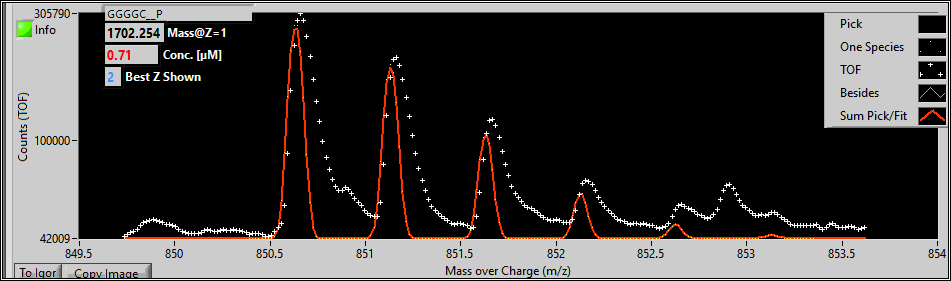

Supplement: Supplementary file 3 — Supplementary Data 1, 2 and 3 [file 41467_2025_60359_MOESM3_ESM.zip › Supplementary Data/Supplementary Data 3/GC-no aa-SI/000030_GGGGC__P.bmp]

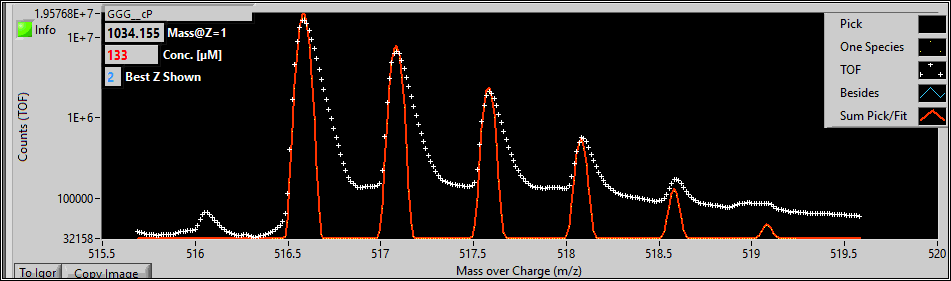

Supplement: Supplementary file 3 — Supplementary Data 1, 2 and 3 [file 41467_2025_60359_MOESM3_ESM.zip › Supplementary Data/Supplementary Data 3/GC-no aa-SI/000011_GGG__cP.bmp]

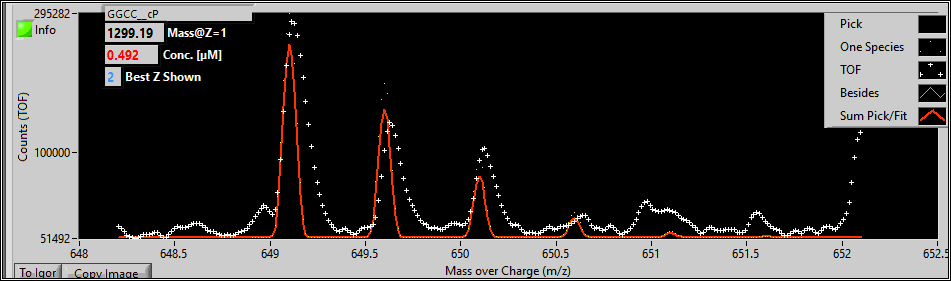

Supplement: Supplementary file 3 — Supplementary Data 1, 2 and 3 [file 41467_2025_60359_MOESM3_ESM.zip › Supplementary Data/Supplementary Data 3/GC-no aa-SI/000023_GGCC__cP.bmp]

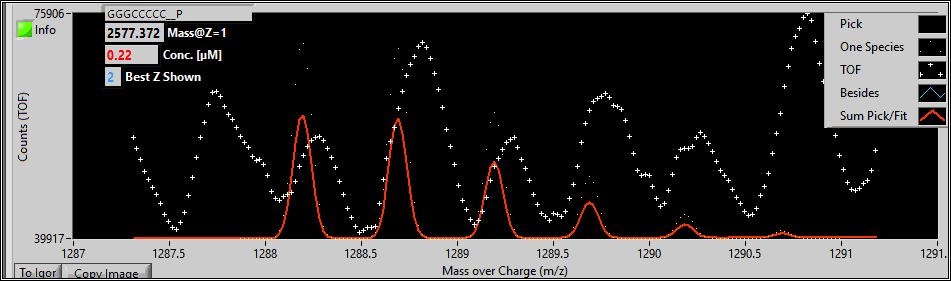

Supplement: Supplementary file 3 — Supplementary Data 1, 2 and 3 [file 41467_2025_60359_MOESM3_ESM.zip › Supplementary Data/Supplementary Data 3/GC-no aa-SI/000080_GGGCCCCC__P.bmp]

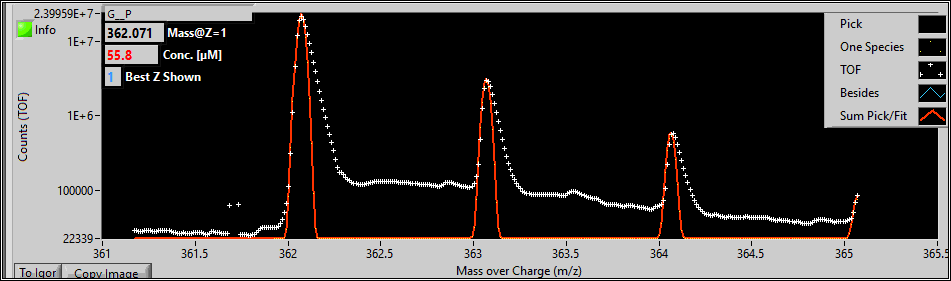

Supplement: Supplementary file 3 — Supplementary Data 1, 2 and 3 [file 41467_2025_60359_MOESM3_ESM.zip › Supplementary Data/Supplementary Data 3/GC-no aa-SI/000000_G__P.bmp]

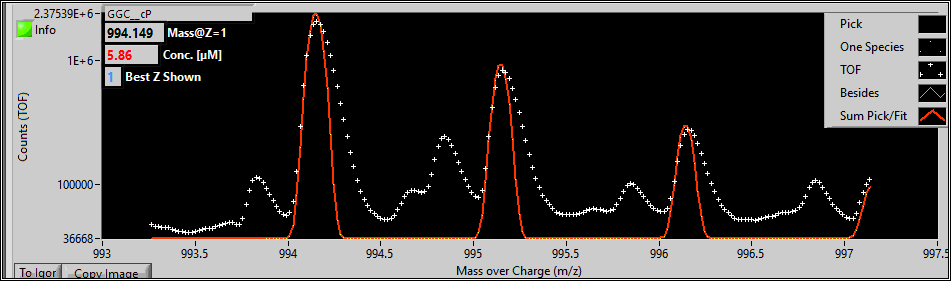

Supplement: Supplementary file 3 — Supplementary Data 1, 2 and 3 [file 41467_2025_60359_MOESM3_ESM.zip › Supplementary Data/Supplementary Data 3/GC-no aa-SI/000013_GGC__cP.bmp]

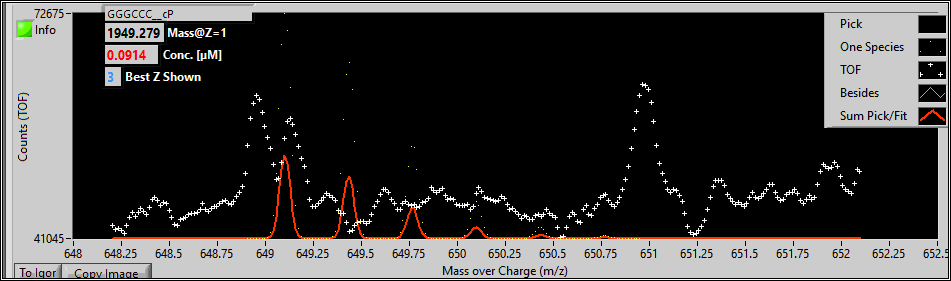

Supplement: Supplementary file 3 — Supplementary Data 1, 2 and 3 [file 41467_2025_60359_MOESM3_ESM.zip › Supplementary Data/Supplementary Data 3/GC-no aa-SI/000047_GGGCCC__cP.bmp]

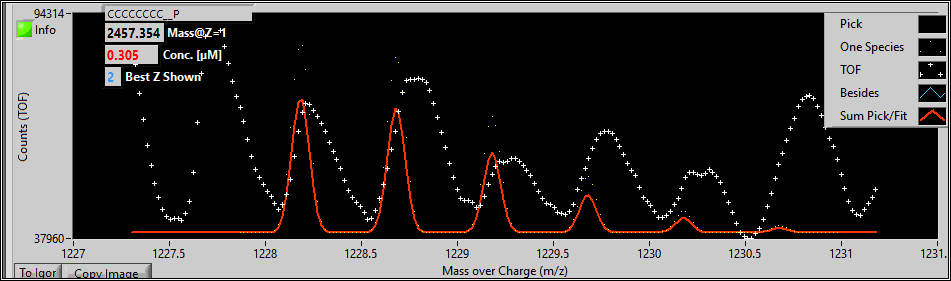

Supplement: Supplementary file 3 — Supplementary Data 1, 2 and 3 [file 41467_2025_60359_MOESM3_ESM.zip › Supplementary Data/Supplementary Data 3/GC-no aa-SI/000086_CCCCCCCC__P.bmp]

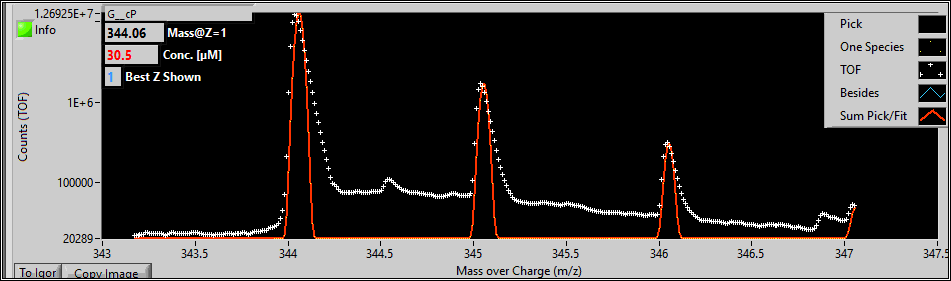

Supplement: Supplementary file 3 — Supplementary Data 1, 2 and 3 [file 41467_2025_60359_MOESM3_ESM.zip › Supplementary Data/Supplementary Data 3/GC-no aa-SI/000001_G__cP.bmp]

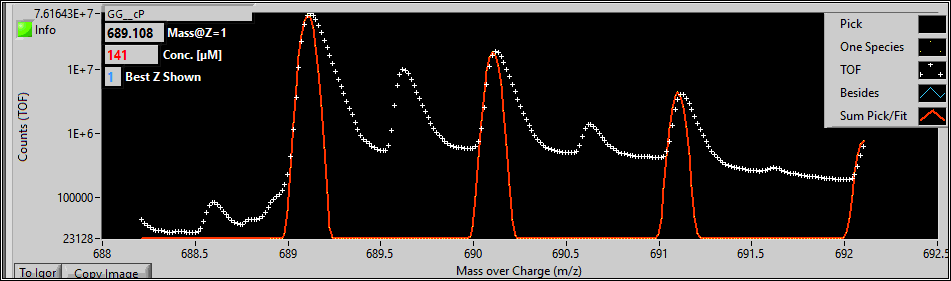

Supplement: Supplementary file 3 — Supplementary Data 1, 2 and 3 [file 41467_2025_60359_MOESM3_ESM.zip › Supplementary Data/Supplementary Data 3/GC-no aa-SI/000005_GG__cP.bmp]

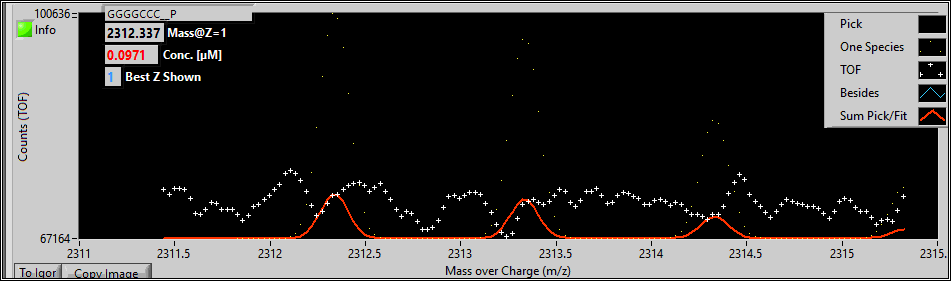

Supplement: Supplementary file 3 — Supplementary Data 1, 2 and 3 [file 41467_2025_60359_MOESM3_ESM.zip › Supplementary Data/Supplementary Data 3/GC-no aa-SI/000060_GGGGCCC__P.bmp]

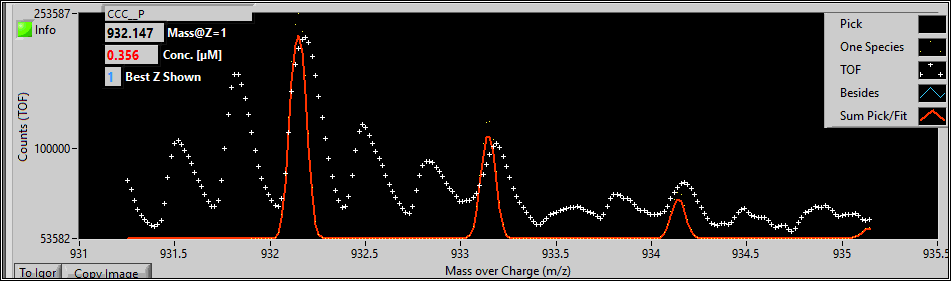

Supplement: Supplementary file 3 — Supplementary Data 1, 2 and 3 [file 41467_2025_60359_MOESM3_ESM.zip › Supplementary Data/Supplementary Data 3/GC-no aa-SI/000016_CCC__P.bmp]

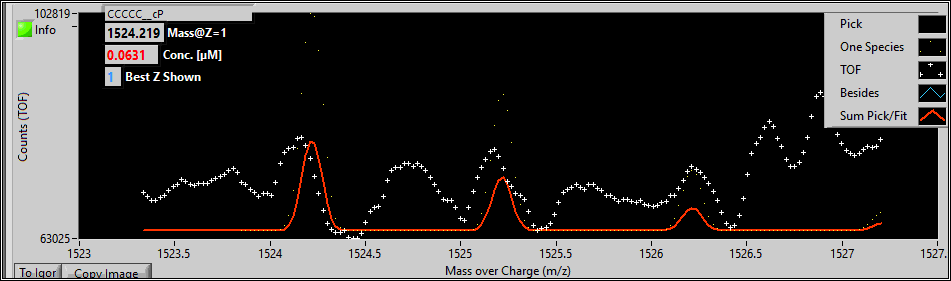

Supplement: Supplementary file 3 — Supplementary Data 1, 2 and 3 [file 41467_2025_60359_MOESM3_ESM.zip › Supplementary Data/Supplementary Data 3/GC-no aa-SI/000039_CCCCC__cP.bmp]

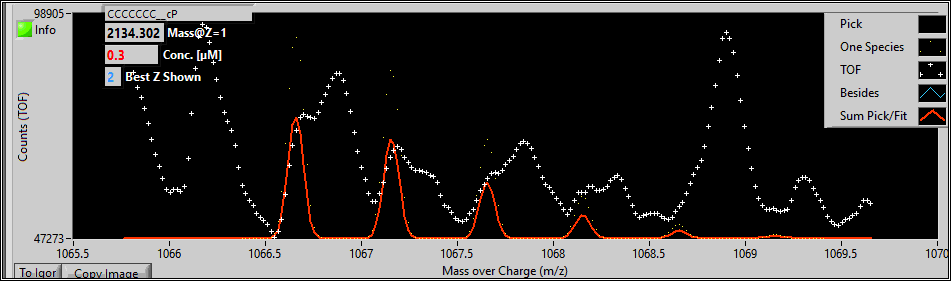

Supplement: Supplementary file 3 — Supplementary Data 1, 2 and 3 [file 41467_2025_60359_MOESM3_ESM.zip › Supplementary Data/Supplementary Data 3/GC-no aa-SI/000069_CCCCCCC__cP.bmp]

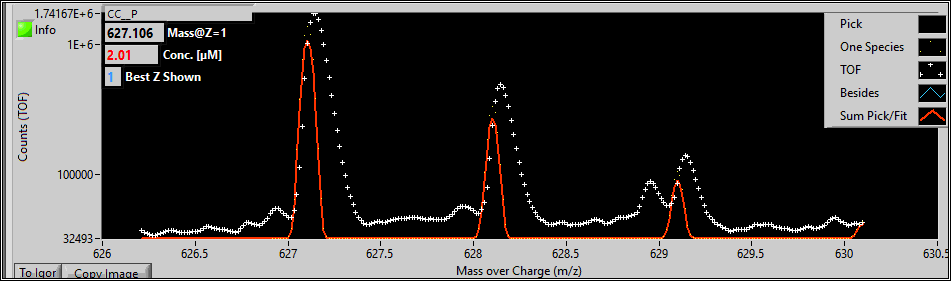

Supplement: Supplementary file 3 — Supplementary Data 1, 2 and 3 [file 41467_2025_60359_MOESM3_ESM.zip › Supplementary Data/Supplementary Data 3/GC-no aa-SI/000008_CC__P.bmp]

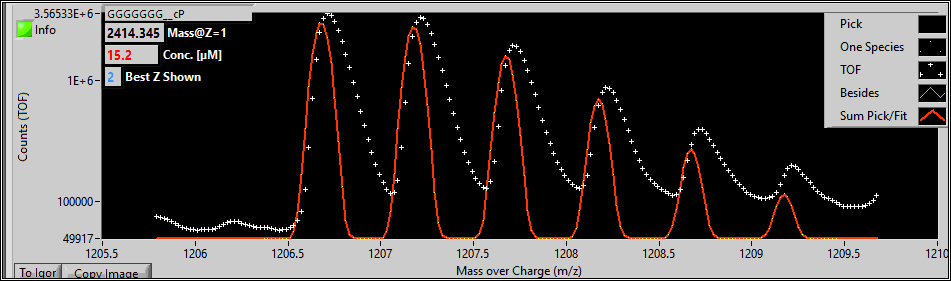

Supplement: Supplementary file 3 — Supplementary Data 1, 2 and 3 [file 41467_2025_60359_MOESM3_ESM.zip › Supplementary Data/Supplementary Data 3/GC-no aa-SI/000055_GGGGGGG__cP.bmp]

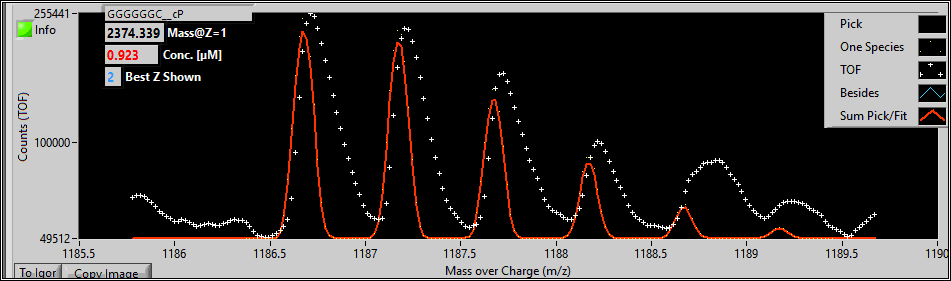

Supplement: Supplementary file 3 — Supplementary Data 1, 2 and 3 [file 41467_2025_60359_MOESM3_ESM.zip › Supplementary Data/Supplementary Data 3/GC-no aa-SI/000057_GGGGGGC__cP.bmp]

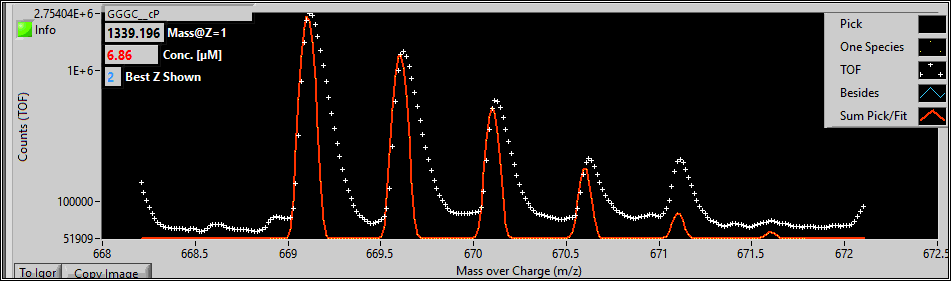

Supplement: Supplementary file 3 — Supplementary Data 1, 2 and 3 [file 41467_2025_60359_MOESM3_ESM.zip › Supplementary Data/Supplementary Data 3/GC-no aa-SI/000021_GGGC__cP.bmp]

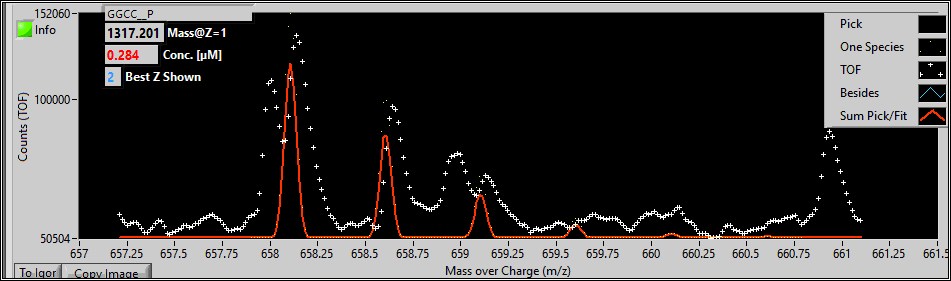

Supplement: Supplementary file 3 — Supplementary Data 1, 2 and 3 [file 41467_2025_60359_MOESM3_ESM.zip › Supplementary Data/Supplementary Data 3/GC-no aa-SI/000022_GGCC__P.bmp]

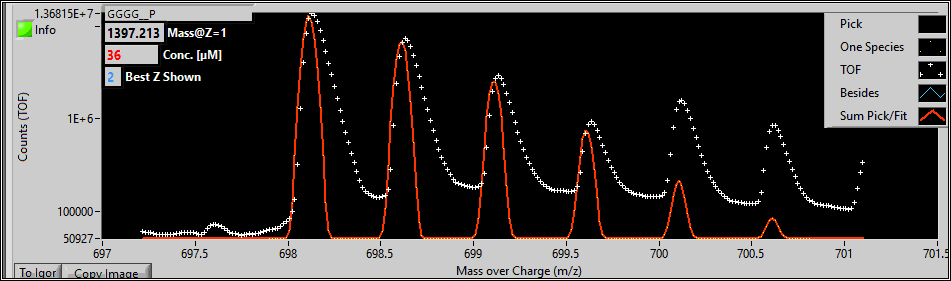

Supplement: Supplementary file 3 — Supplementary Data 1, 2 and 3 [file 41467_2025_60359_MOESM3_ESM.zip › Supplementary Data/Supplementary Data 3/GC-no aa-SI/000018_GGGG__P.bmp]

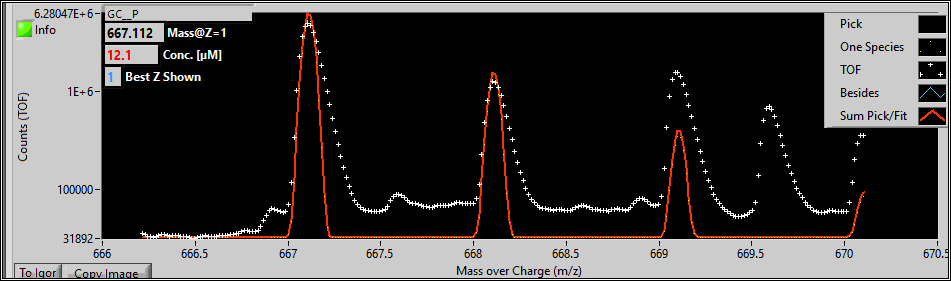

Supplement: Supplementary file 3 — Supplementary Data 1, 2 and 3 [file 41467_2025_60359_MOESM3_ESM.zip › Supplementary Data/Supplementary Data 3/GC-no aa-SI/000006_GC__P.bmp]

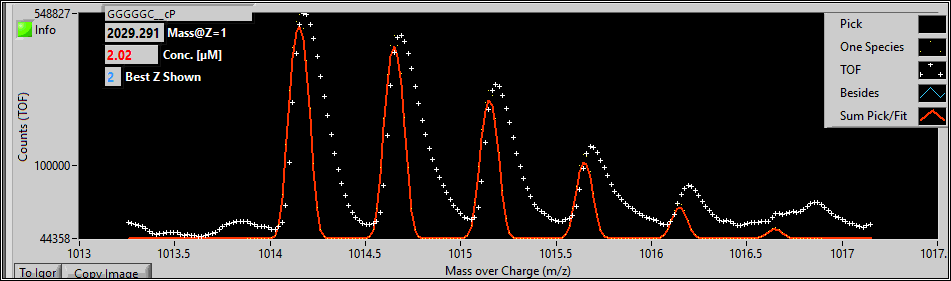

Supplement: Supplementary file 3 — Supplementary Data 1, 2 and 3 [file 41467_2025_60359_MOESM3_ESM.zip › Supplementary Data/Supplementary Data 3/GC-no aa-SI/000043_GGGGGC__cP.bmp]

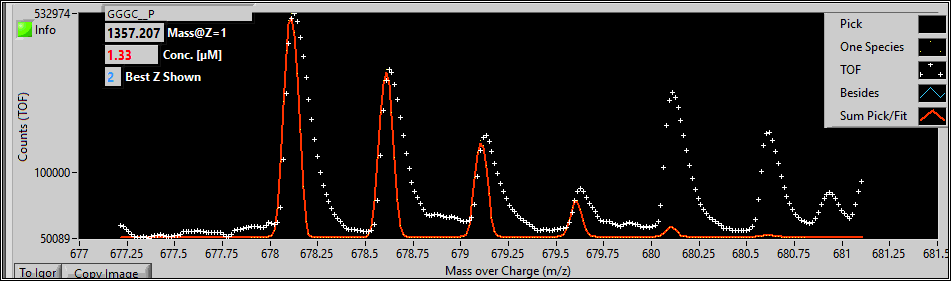

Supplement: Supplementary file 3 — Supplementary Data 1, 2 and 3 [file 41467_2025_60359_MOESM3_ESM.zip › Supplementary Data/Supplementary Data 3/GC-no aa-SI/000020_GGGC__P.bmp]

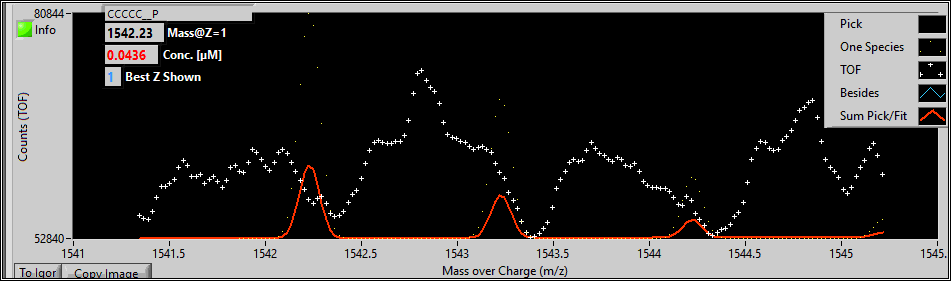

Supplement: Supplementary file 3 — Supplementary Data 1, 2 and 3 [file 41467_2025_60359_MOESM3_ESM.zip › Supplementary Data/Supplementary Data 3/GC-no aa-SI/000038_CCCCC__P.bmp]

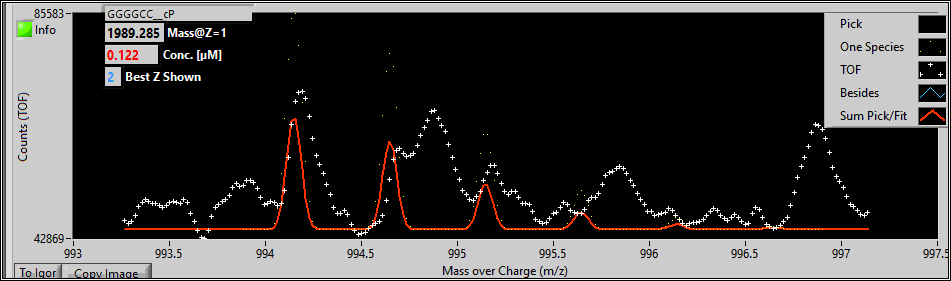

Supplement: Supplementary file 3 — Supplementary Data 1, 2 and 3 [file 41467_2025_60359_MOESM3_ESM.zip › Supplementary Data/Supplementary Data 3/GC-no aa-SI/000045_GGGGCC__cP.bmp]

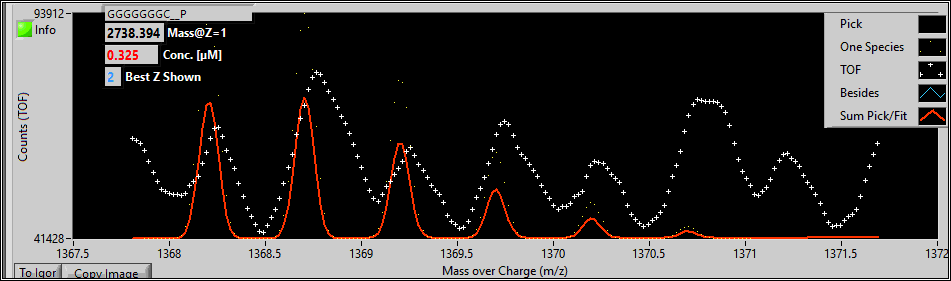

Supplement: Supplementary file 3 — Supplementary Data 1, 2 and 3 [file 41467_2025_60359_MOESM3_ESM.zip › Supplementary Data/Supplementary Data 3/GC-no aa-SI/000072_GGGGGGGC__P.bmp]

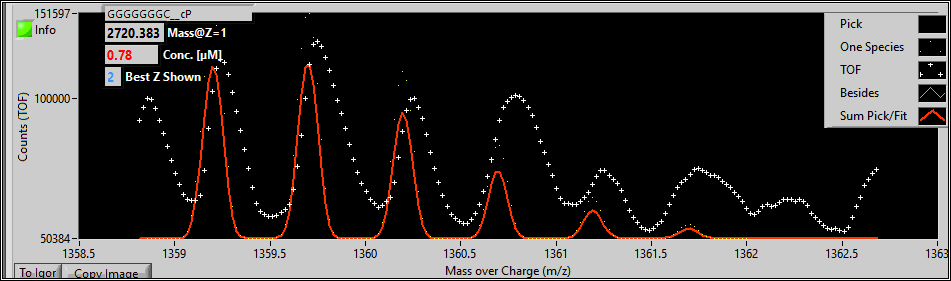

Supplement: Supplementary file 3 — Supplementary Data 1, 2 and 3 [file 41467_2025_60359_MOESM3_ESM.zip › Supplementary Data/Supplementary Data 3/GC-no aa-SI/000073_GGGGGGGC__cP.bmp]

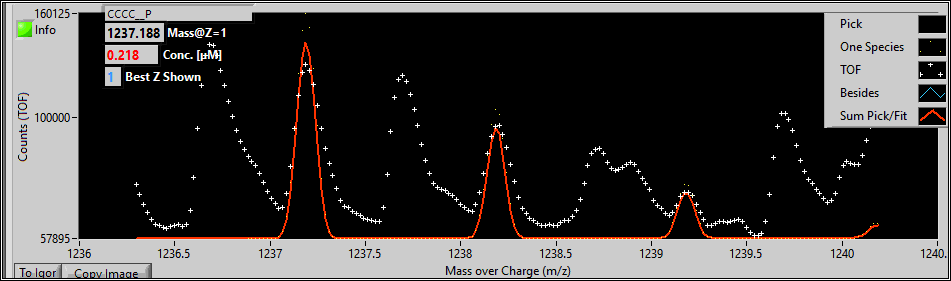

Supplement: Supplementary file 3 — Supplementary Data 1, 2 and 3 [file 41467_2025_60359_MOESM3_ESM.zip › Supplementary Data/Supplementary Data 3/GC-no aa-SI/000026_CCCC__P.bmp]

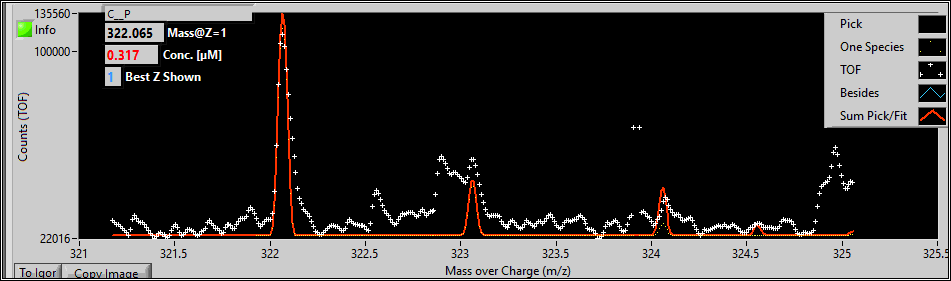

Supplement: Supplementary file 3 — Supplementary Data 1, 2 and 3 [file 41467_2025_60359_MOESM3_ESM.zip › Supplementary Data/Supplementary Data 3/GC-no aa-SI/000002_C__P.bmp]

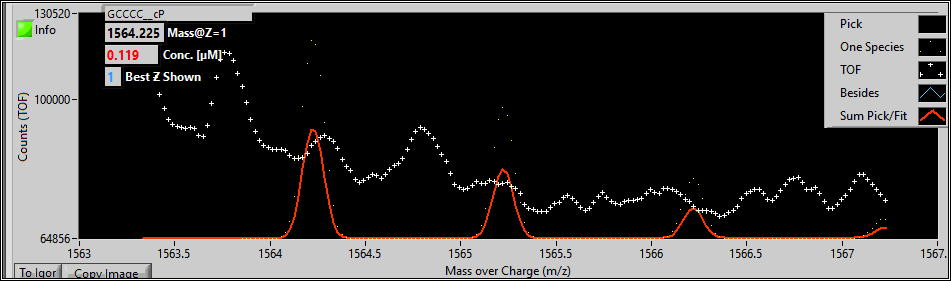

Supplement: Supplementary file 3 — Supplementary Data 1, 2 and 3 [file 41467_2025_60359_MOESM3_ESM.zip › Supplementary Data/Supplementary Data 3/GC-no aa-SI/000037_GCCCC__cP.bmp]

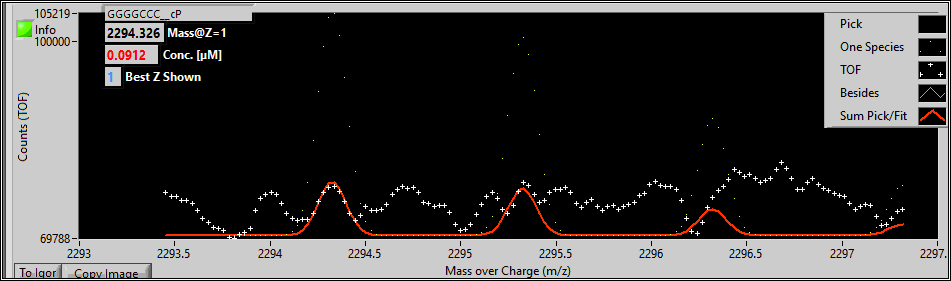

Supplement: Supplementary file 3 — Supplementary Data 1, 2 and 3 [file 41467_2025_60359_MOESM3_ESM.zip › Supplementary Data/Supplementary Data 3/GC-no aa-SI/000061_GGGGCCC__cP.bmp]

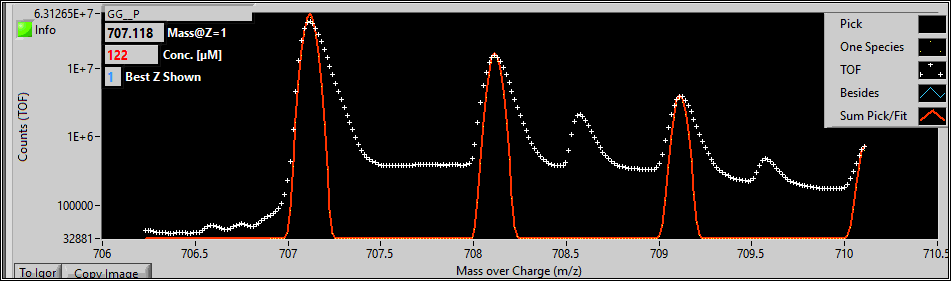

Supplement: Supplementary file 3 — Supplementary Data 1, 2 and 3 [file 41467_2025_60359_MOESM3_ESM.zip › Supplementary Data/Supplementary Data 3/GC-no aa-SI/000004_GG__P.bmp]

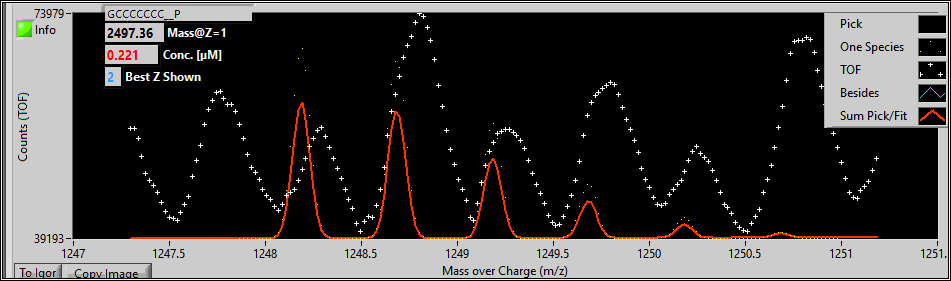

Supplement: Supplementary file 3 — Supplementary Data 1, 2 and 3 [file 41467_2025_60359_MOESM3_ESM.zip › Supplementary Data/Supplementary Data 3/GC-no aa-SI/000084_GCCCCCCC__P.bmp]

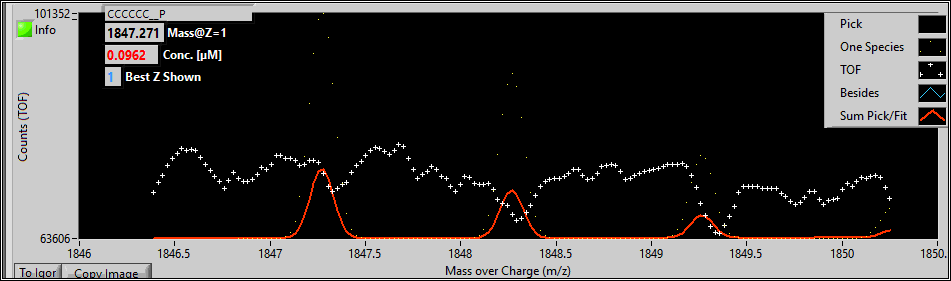

Supplement: Supplementary file 3 — Supplementary Data 1, 2 and 3 [file 41467_2025_60359_MOESM3_ESM.zip › Supplementary Data/Supplementary Data 3/GC-no aa-SI/000052_CCCCCC__P.bmp]

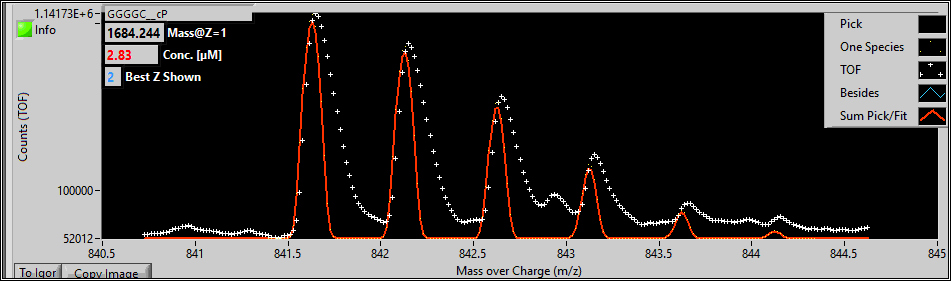

Supplement: Supplementary file 3 — Supplementary Data 1, 2 and 3 [file 41467_2025_60359_MOESM3_ESM.zip › Supplementary Data/Supplementary Data 3/GC-no aa-SI/000031_GGGGC__cP.bmp]

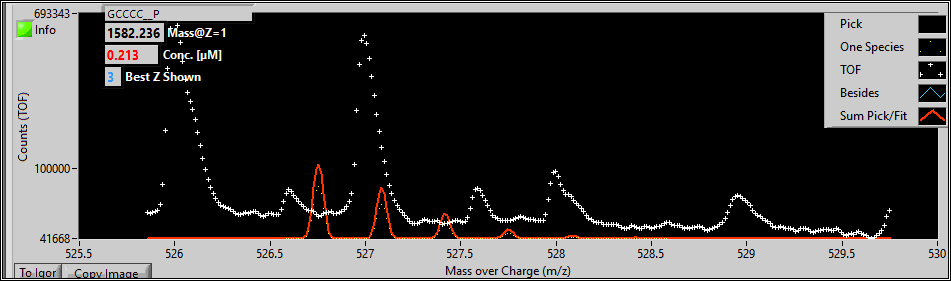

Supplement: Supplementary file 3 — Supplementary Data 1, 2 and 3 [file 41467_2025_60359_MOESM3_ESM.zip › Supplementary Data/Supplementary Data 3/GC-no aa-SI/000036_GCCCC__P.bmp]

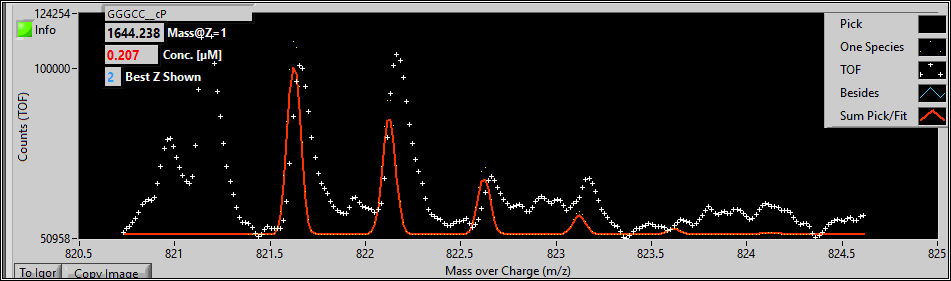

Supplement: Supplementary file 3 — Supplementary Data 1, 2 and 3 [file 41467_2025_60359_MOESM3_ESM.zip › Supplementary Data/Supplementary Data 3/GC-no aa-SI/000033_GGGCC__cP.bmp]

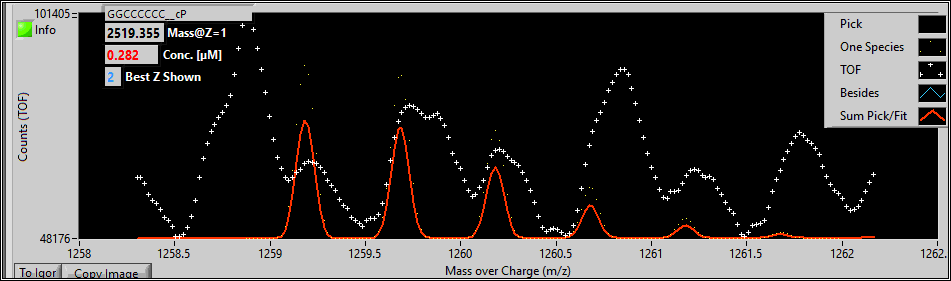

Supplement: Supplementary file 3 — Supplementary Data 1, 2 and 3 [file 41467_2025_60359_MOESM3_ESM.zip › Supplementary Data/Supplementary Data 3/GC-no aa-SI/000083_GGCCCCCC__cP.bmp]

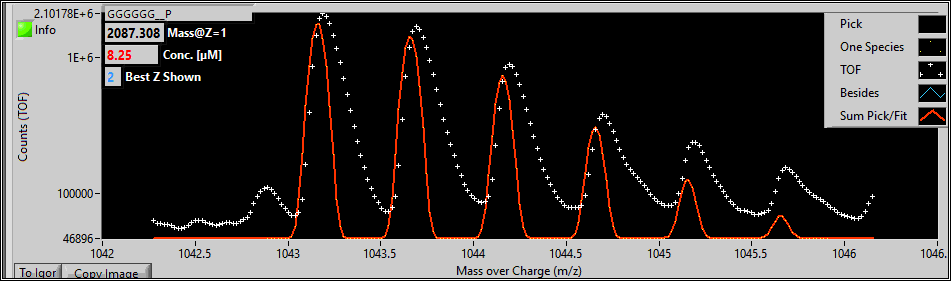

Supplement: Supplementary file 3 — Supplementary Data 1, 2 and 3 [file 41467_2025_60359_MOESM3_ESM.zip › Supplementary Data/Supplementary Data 3/GC-no aa-SI/000040_GGGGGG__P.bmp]

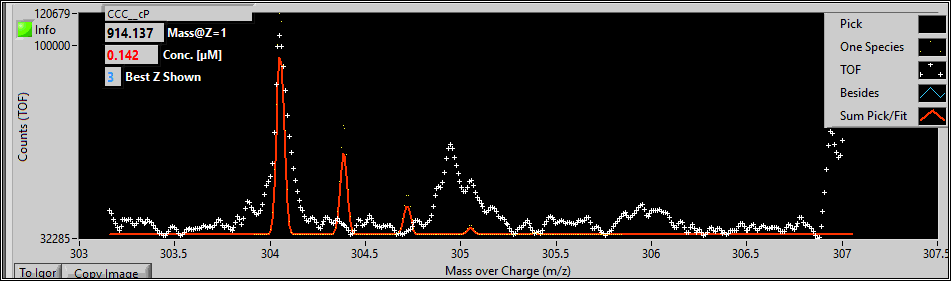

Supplement: Supplementary file 3 — Supplementary Data 1, 2 and 3 [file 41467_2025_60359_MOESM3_ESM.zip › Supplementary Data/Supplementary Data 3/GC-no aa-SI/000017_CCC__cP.bmp]

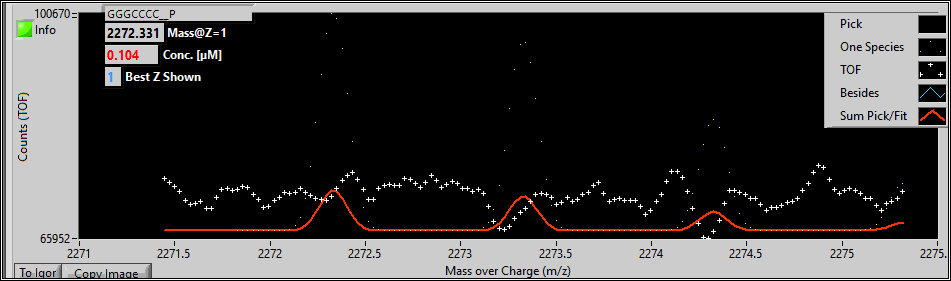

Supplement: Supplementary file 3 — Supplementary Data 1, 2 and 3 [file 41467_2025_60359_MOESM3_ESM.zip › Supplementary Data/Supplementary Data 3/GC-no aa-SI/000062_GGGCCCC__P.bmp]

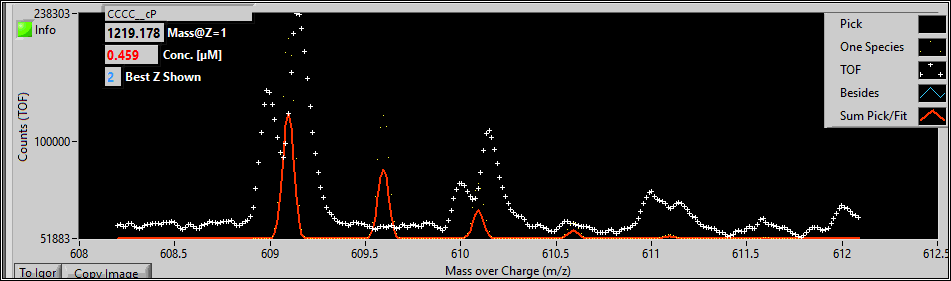

Supplement: Supplementary file 3 — Supplementary Data 1, 2 and 3 [file 41467_2025_60359_MOESM3_ESM.zip › Supplementary Data/Supplementary Data 3/GC-no aa-SI/000027_CCCC__cP.bmp]

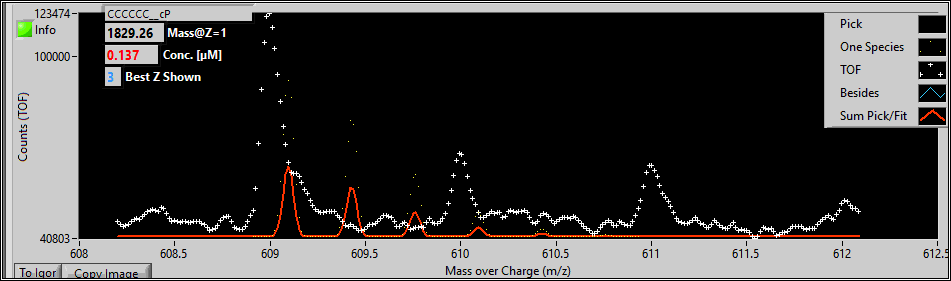

Supplement: Supplementary file 3 — Supplementary Data 1, 2 and 3 [file 41467_2025_60359_MOESM3_ESM.zip › Supplementary Data/Supplementary Data 3/GC-no aa-SI/000053_CCCCCC__cP.bmp]

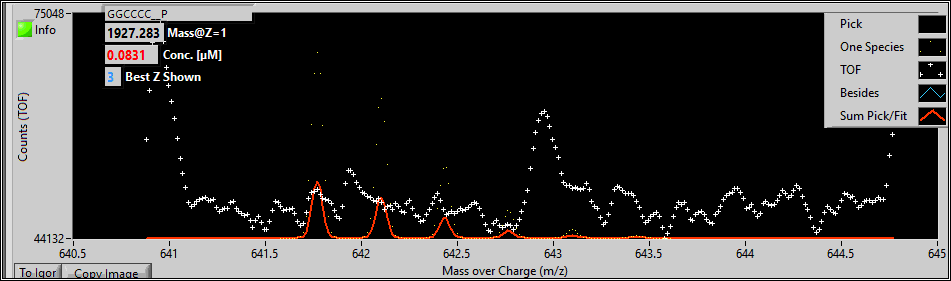

Supplement: Supplementary file 3 — Supplementary Data 1, 2 and 3 [file 41467_2025_60359_MOESM3_ESM.zip › Supplementary Data/Supplementary Data 3/GC-no aa-SI/000048_GGCCCC__P.bmp]

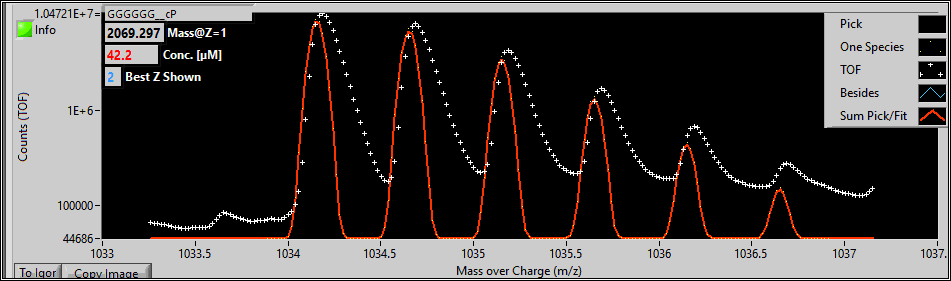

Supplement: Supplementary file 3 — Supplementary Data 1, 2 and 3 [file 41467_2025_60359_MOESM3_ESM.zip › Supplementary Data/Supplementary Data 3/GC-no aa-SI/000041_GGGGGG__cP.bmp]

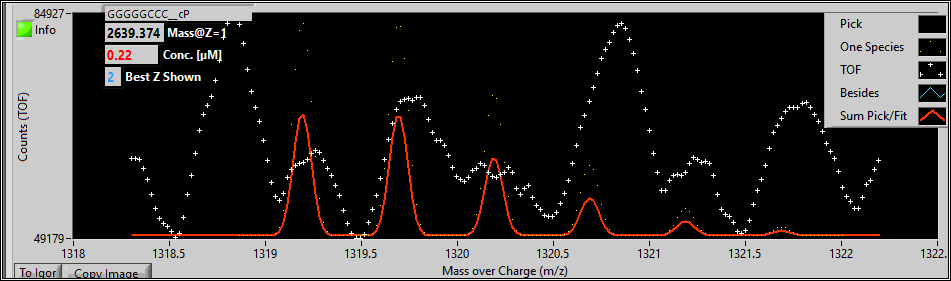

Supplement: Supplementary file 3 — Supplementary Data 1, 2 and 3 [file 41467_2025_60359_MOESM3_ESM.zip › Supplementary Data/Supplementary Data 3/GC-no aa-SI/000077_GGGGGCCC__cP.bmp]

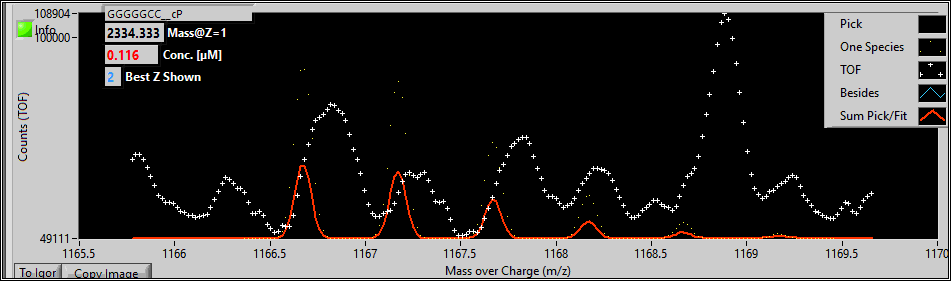

Supplement: Supplementary file 3 — Supplementary Data 1, 2 and 3 [file 41467_2025_60359_MOESM3_ESM.zip › Supplementary Data/Supplementary Data 3/GC-no aa-SI/000059_GGGGGCC__cP.bmp]

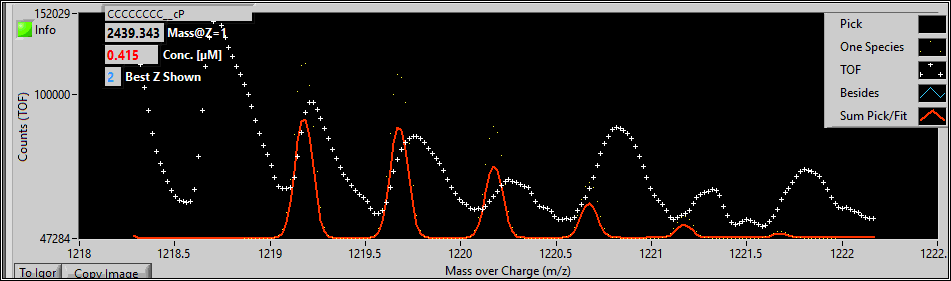

Supplement: Supplementary file 3 — Supplementary Data 1, 2 and 3 [file 41467_2025_60359_MOESM3_ESM.zip › Supplementary Data/Supplementary Data 3/GC-no aa-SI/000087_CCCCCCCC__cP.bmp]

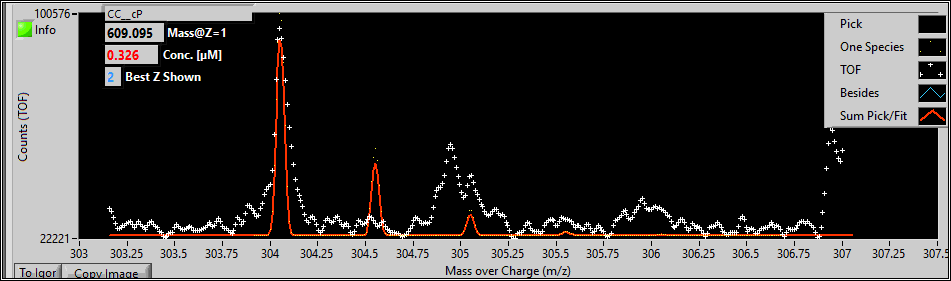

Supplement: Supplementary file 3 — Supplementary Data 1, 2 and 3 [file 41467_2025_60359_MOESM3_ESM.zip › Supplementary Data/Supplementary Data 3/GC-no aa-SI/000009_CC__cP.bmp]

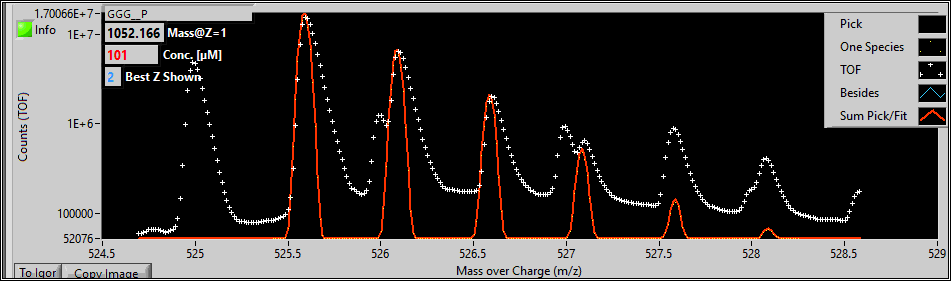

Supplement: Supplementary file 3 — Supplementary Data 1, 2 and 3 [file 41467_2025_60359_MOESM3_ESM.zip › Supplementary Data/Supplementary Data 3/GC-no aa-SI/000010_GGG__P.bmp]

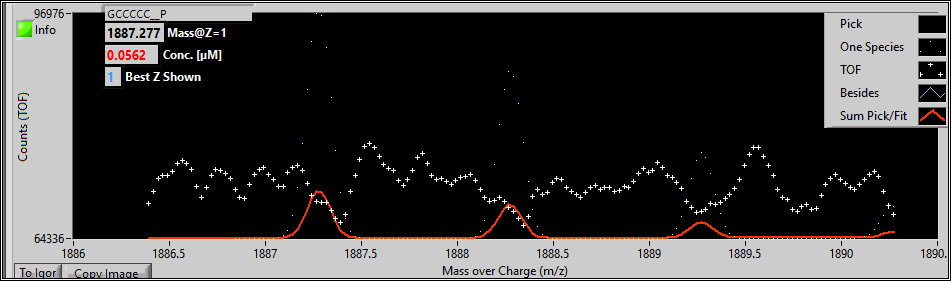

Supplement: Supplementary file 3 — Supplementary Data 1, 2 and 3 [file 41467_2025_60359_MOESM3_ESM.zip › Supplementary Data/Supplementary Data 3/GC-no aa-SI/000050_GCCCCC__P.bmp]

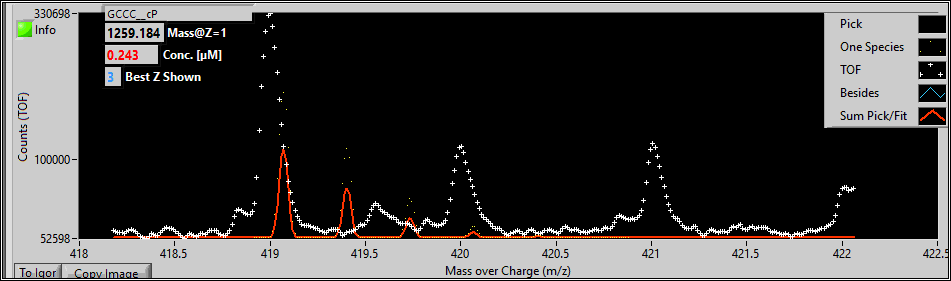

Supplement: Supplementary file 3 — Supplementary Data 1, 2 and 3 [file 41467_2025_60359_MOESM3_ESM.zip › Supplementary Data/Supplementary Data 3/GC-no aa-SI/000025_GCCC__cP.bmp]

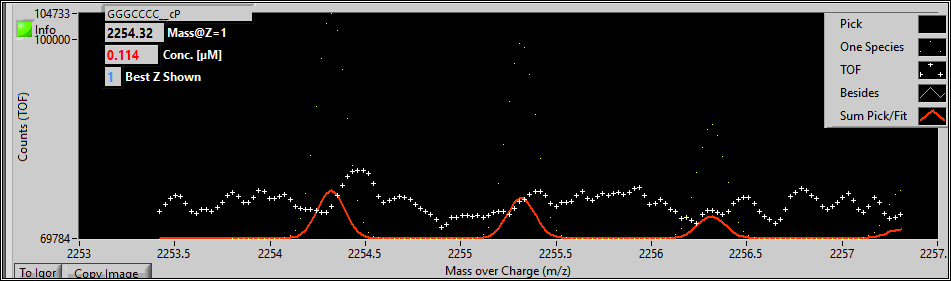

Supplement: Supplementary file 3 — Supplementary Data 1, 2 and 3 [file 41467_2025_60359_MOESM3_ESM.zip › Supplementary Data/Supplementary Data 3/GC-no aa-SI/000063_GGGCCCC__cP.bmp]

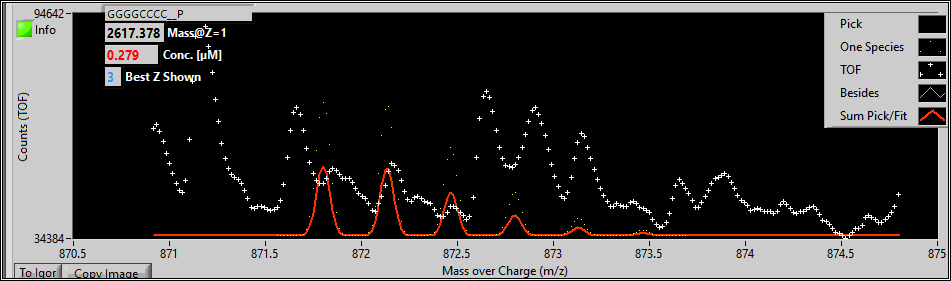

Supplement: Supplementary file 3 — Supplementary Data 1, 2 and 3 [file 41467_2025_60359_MOESM3_ESM.zip › Supplementary Data/Supplementary Data 3/GC-no aa-SI/000078_GGGGCCCC__P.bmp]

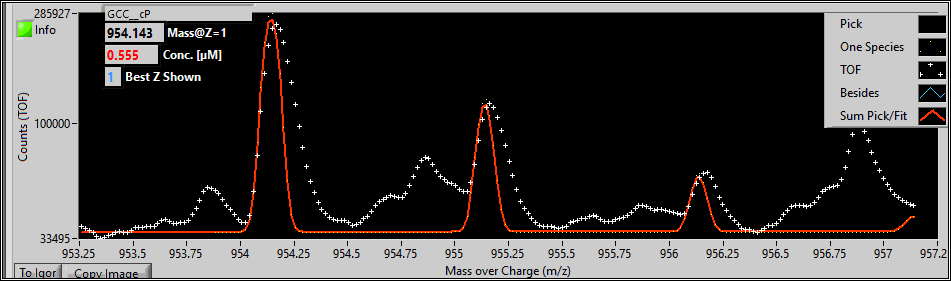

Supplement: Supplementary file 3 — Supplementary Data 1, 2 and 3 [file 41467_2025_60359_MOESM3_ESM.zip › Supplementary Data/Supplementary Data 3/GC-no aa-SI/000015_GCC__cP.bmp]

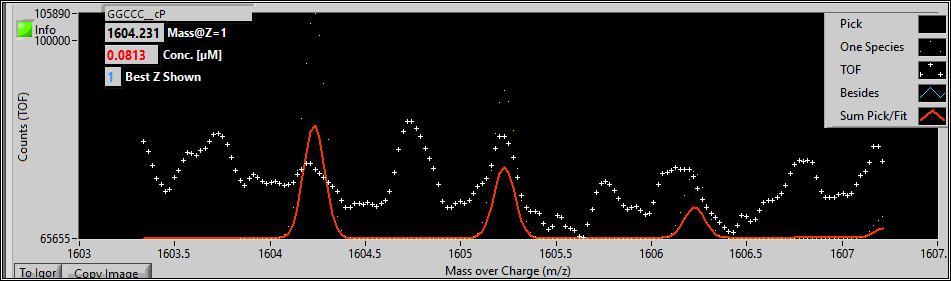

Supplement: Supplementary file 3 — Supplementary Data 1, 2 and 3 [file 41467_2025_60359_MOESM3_ESM.zip › Supplementary Data/Supplementary Data 3/GC-no aa-SI/000035_GGCCC__cP.bmp]

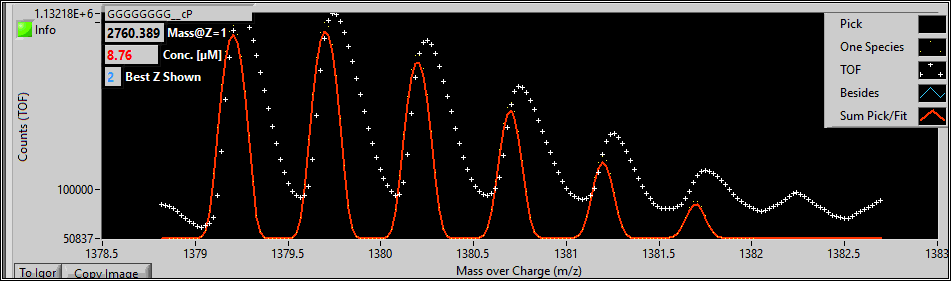

Supplement: Supplementary file 3 — Supplementary Data 1, 2 and 3 [file 41467_2025_60359_MOESM3_ESM.zip › Supplementary Data/Supplementary Data 3/GC-no aa-SI/000071_GGGGGGGG__cP.bmp]

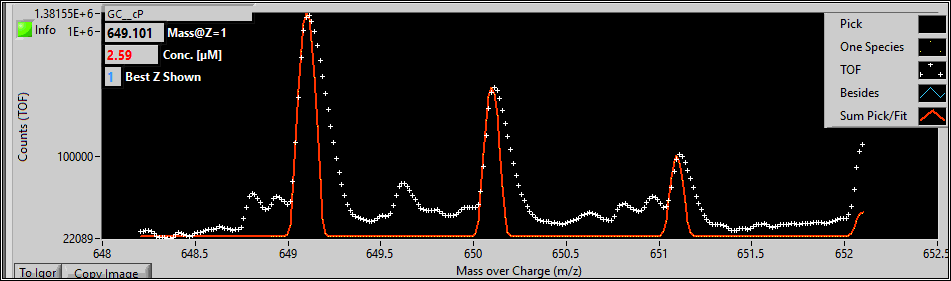

Supplement: Supplementary file 3 — Supplementary Data 1, 2 and 3 [file 41467_2025_60359_MOESM3_ESM.zip › Supplementary Data/Supplementary Data 3/GC-no aa-SI/000007_GC__cP.bmp]

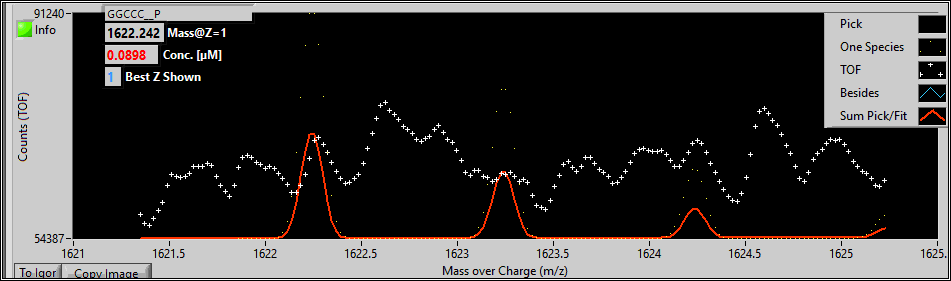

Supplement: Supplementary file 3 — Supplementary Data 1, 2 and 3 [file 41467_2025_60359_MOESM3_ESM.zip › Supplementary Data/Supplementary Data 3/GC-no aa-SI/000034_GGCCC__P.bmp]

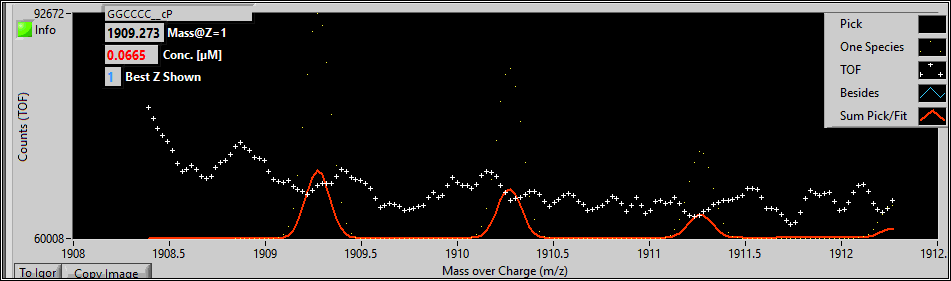

Supplement: Supplementary file 3 — Supplementary Data 1, 2 and 3 [file 41467_2025_60359_MOESM3_ESM.zip › Supplementary Data/Supplementary Data 3/GC-no aa-SI/000049_GGCCCC__cP.bmp]

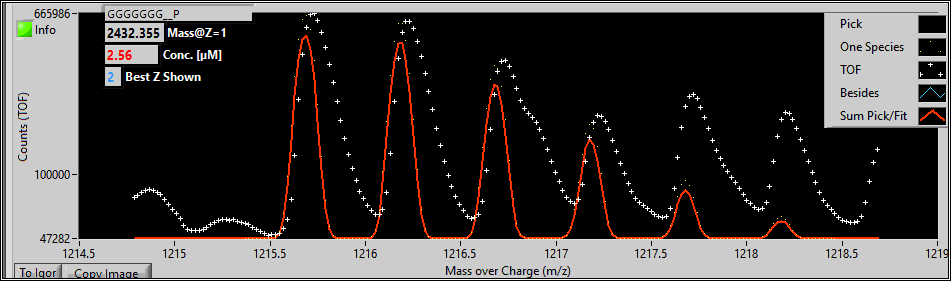

Supplement: Supplementary file 3 — Supplementary Data 1, 2 and 3 [file 41467_2025_60359_MOESM3_ESM.zip › Supplementary Data/Supplementary Data 3/GC-no aa-SI/000054_GGGGGGG__P.bmp]

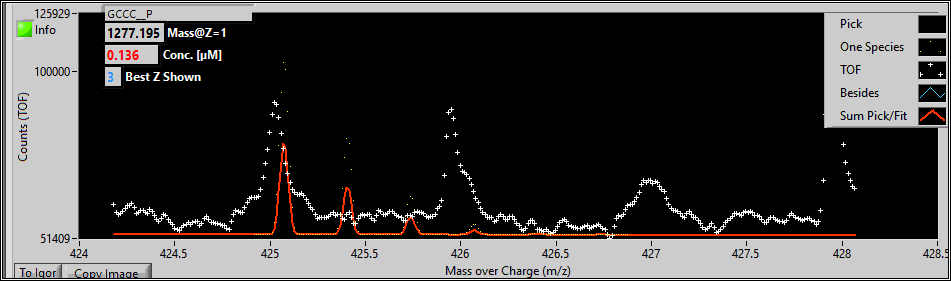

Supplement: Supplementary file 3 — Supplementary Data 1, 2 and 3 [file 41467_2025_60359_MOESM3_ESM.zip › Supplementary Data/Supplementary Data 3/GC-no aa-SI/000024_GCCC__P.bmp]

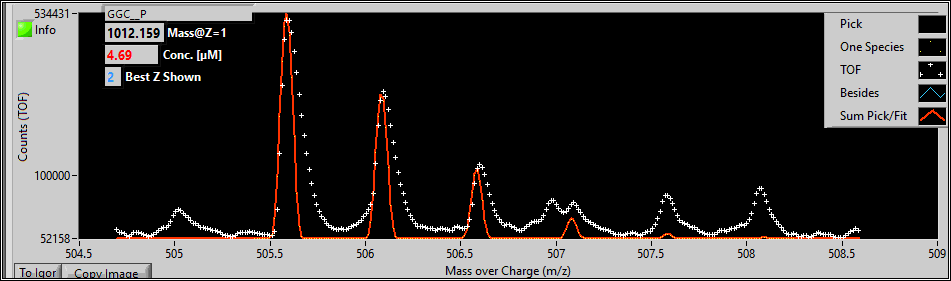

Supplement: Supplementary file 3 — Supplementary Data 1, 2 and 3 [file 41467_2025_60359_MOESM3_ESM.zip › Supplementary Data/Supplementary Data 3/GC-no aa-SI/000012_GGC__P.bmp]

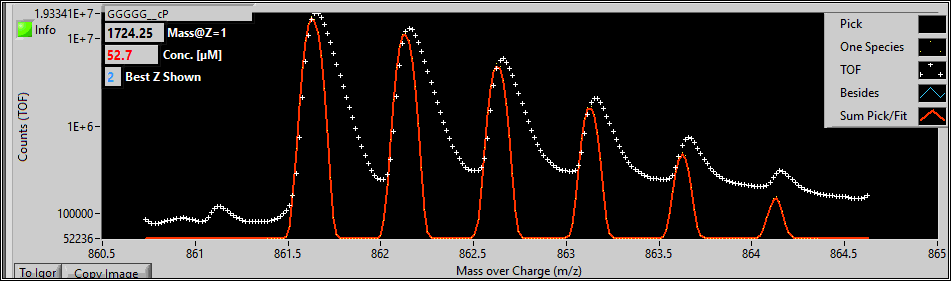

Supplement: Supplementary file 3 — Supplementary Data 1, 2 and 3 [file 41467_2025_60359_MOESM3_ESM.zip › Supplementary Data/Supplementary Data 3/GC-no aa-SI/000029_GGGGG__cP.bmp]

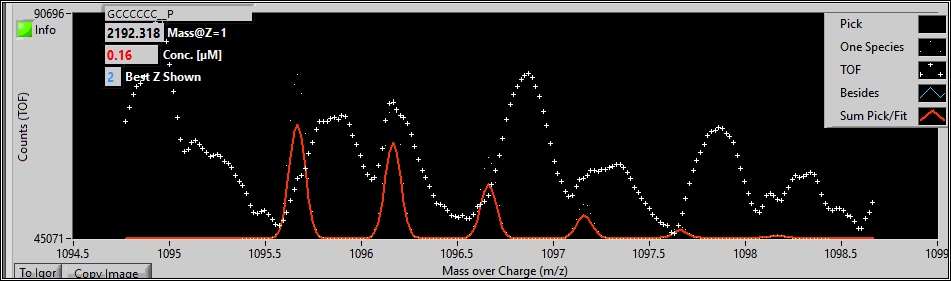

Supplement: Supplementary file 3 — Supplementary Data 1, 2 and 3 [file 41467_2025_60359_MOESM3_ESM.zip › Supplementary Data/Supplementary Data 3/GC-no aa-SI/000066_GCCCCCC__P.bmp]

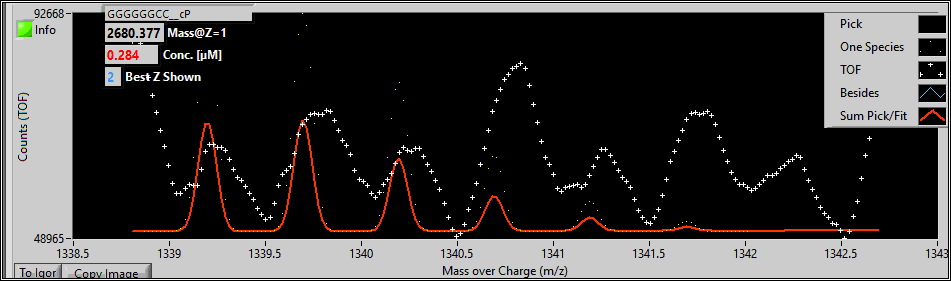

Supplement: Supplementary file 3 — Supplementary Data 1, 2 and 3 [file 41467_2025_60359_MOESM3_ESM.zip › Supplementary Data/Supplementary Data 3/GC-no aa-SI/000075_GGGGGGCC__cP.bmp]

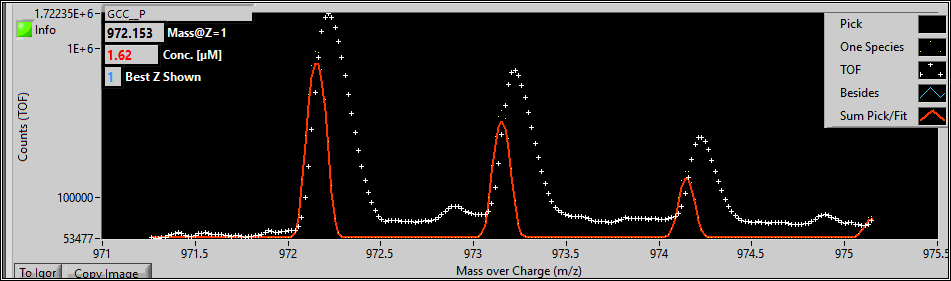

Supplement: Supplementary file 3 — Supplementary Data 1, 2 and 3 [file 41467_2025_60359_MOESM3_ESM.zip › Supplementary Data/Supplementary Data 3/GC-no aa-SI/000014_GCC__P.bmp]

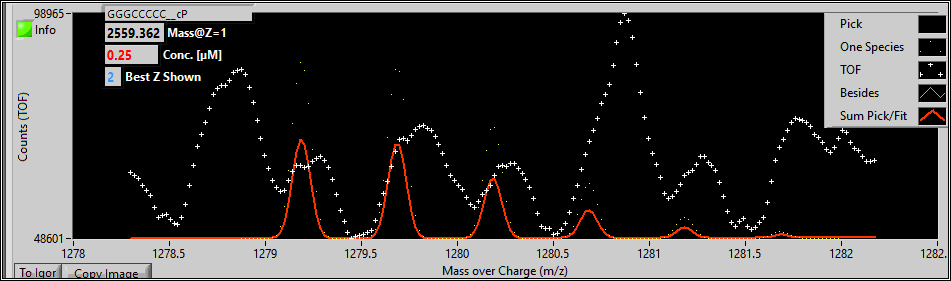

Supplement: Supplementary file 3 — Supplementary Data 1, 2 and 3 [file 41467_2025_60359_MOESM3_ESM.zip › Supplementary Data/Supplementary Data 3/GC-no aa-SI/000081_GGGCCCCC__cP.bmp]

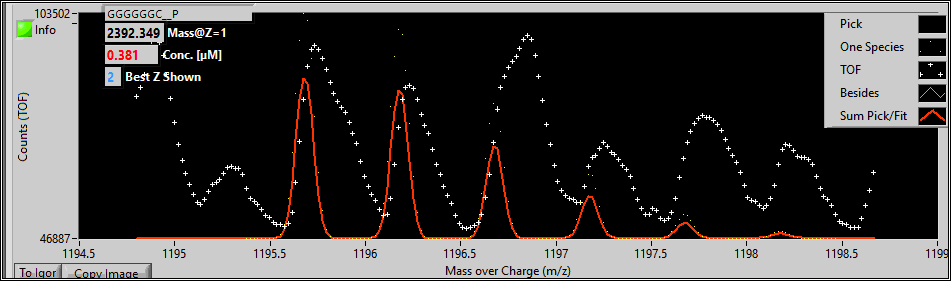

Supplement: Supplementary file 3 — Supplementary Data 1, 2 and 3 [file 41467_2025_60359_MOESM3_ESM.zip › Supplementary Data/Supplementary Data 3/GC-no aa-SI/000056_GGGGGGC__P.bmp]

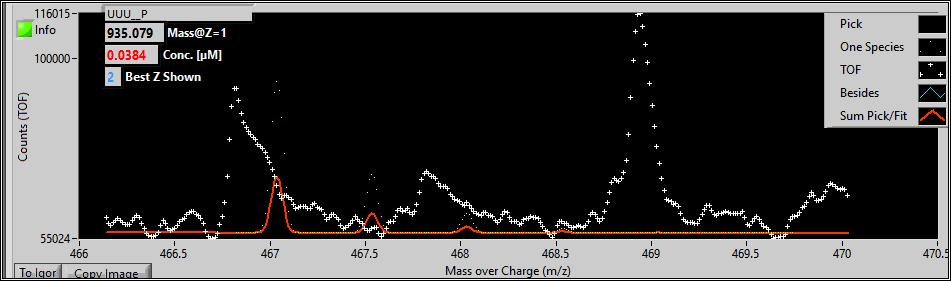

Supplement: Supplementary file 3 — Supplementary Data 1, 2 and 3 [file 41467_2025_60359_MOESM3_ESM.zip › Supplementary Data/Supplementary Data 3/AU-no aa/000012_UUU__P.bmp]

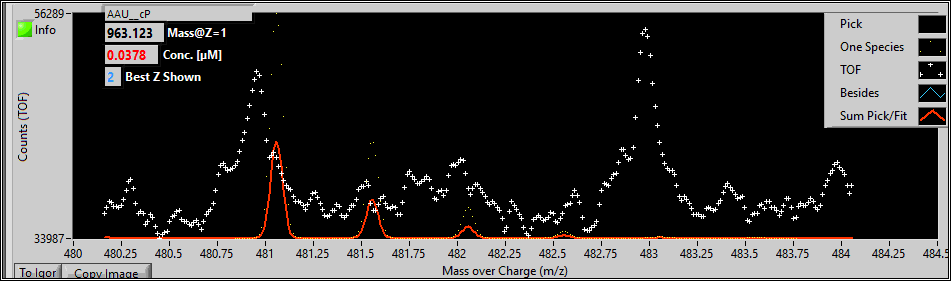

Supplement: Supplementary file 3 — Supplementary Data 1, 2 and 3 [file 41467_2025_60359_MOESM3_ESM.zip › Supplementary Data/Supplementary Data 3/AU-no aa/000009_AAU__cP.bmp]

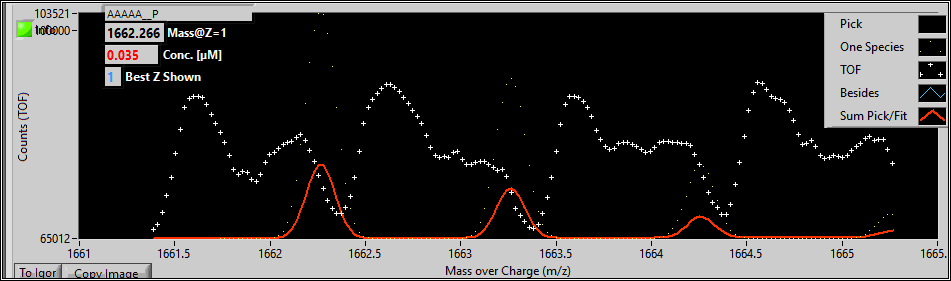

Supplement: Supplementary file 3 — Supplementary Data 1, 2 and 3 [file 41467_2025_60359_MOESM3_ESM.zip › Supplementary Data/Supplementary Data 3/AU-no aa/000024_AAAAA__P.bmp]

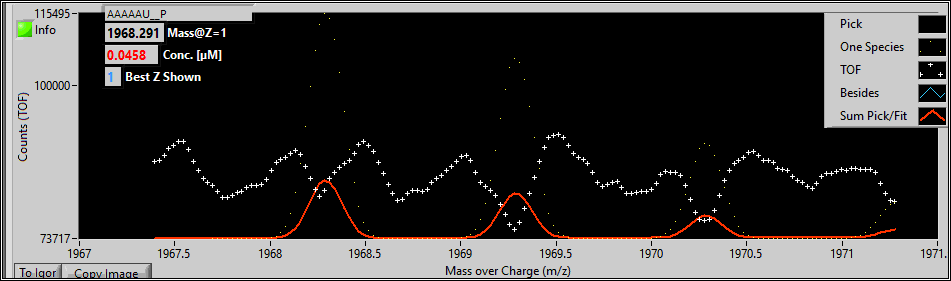

Supplement: Supplementary file 3 — Supplementary Data 1, 2 and 3 [file 41467_2025_60359_MOESM3_ESM.zip › Supplementary Data/Supplementary Data 3/AU-no aa/000038_AAAAAU__P.bmp]

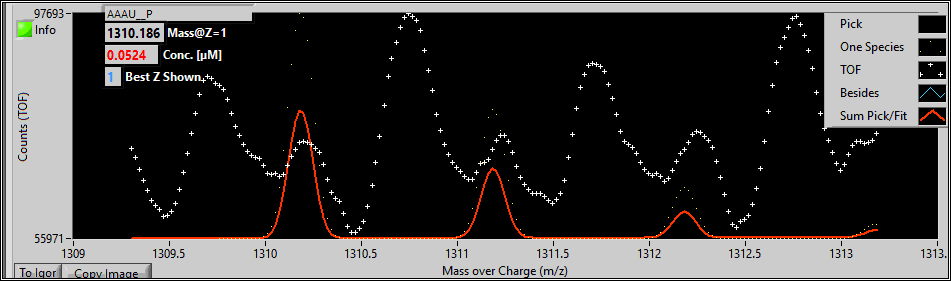

Supplement: Supplementary file 3 — Supplementary Data 1, 2 and 3 [file 41467_2025_60359_MOESM3_ESM.zip › Supplementary Data/Supplementary Data 3/AU-no aa/000016_AAAU__P.bmp]

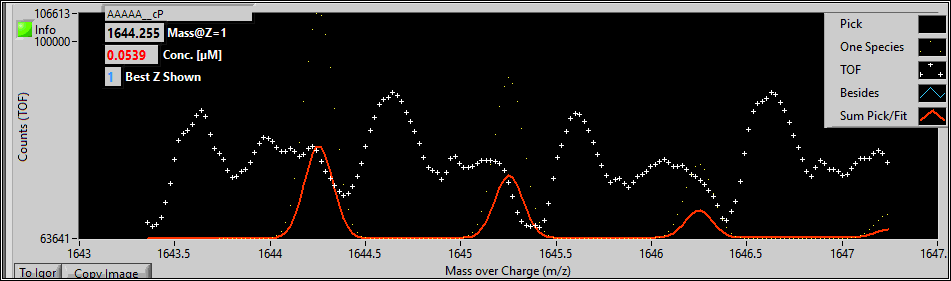

Supplement: Supplementary file 3 — Supplementary Data 1, 2 and 3 [file 41467_2025_60359_MOESM3_ESM.zip › Supplementary Data/Supplementary Data 3/AU-no aa/000025_AAAAA__cP.bmp]

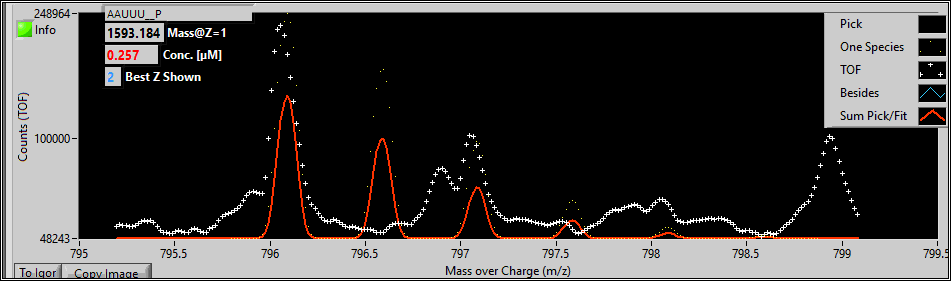

Supplement: Supplementary file 3 — Supplementary Data 1, 2 and 3 [file 41467_2025_60359_MOESM3_ESM.zip › Supplementary Data/Supplementary Data 3/AU-no aa/000030_AAUUU__P.bmp]

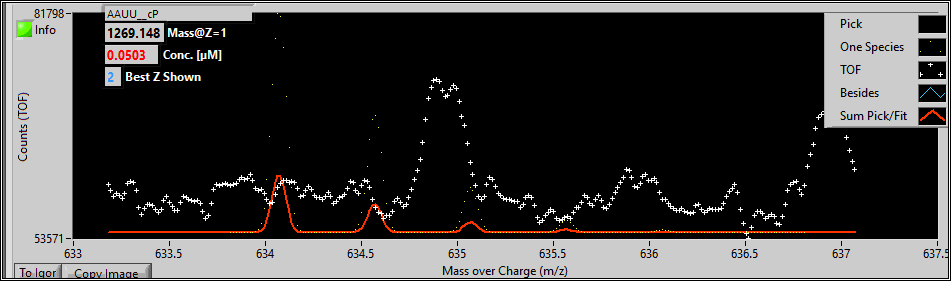

Supplement: Supplementary file 3 — Supplementary Data 1, 2 and 3 [file 41467_2025_60359_MOESM3_ESM.zip › Supplementary Data/Supplementary Data 3/AU-no aa/000019_AAUU__cP.bmp]

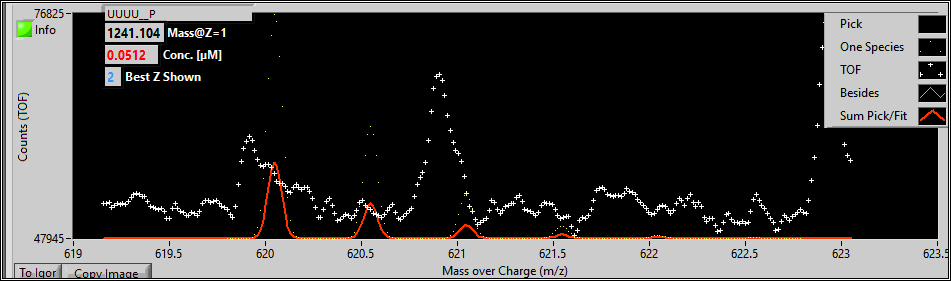

Supplement: Supplementary file 3 — Supplementary Data 1, 2 and 3 [file 41467_2025_60359_MOESM3_ESM.zip › Supplementary Data/Supplementary Data 3/AU-no aa/000022_UUUU__P.bmp]

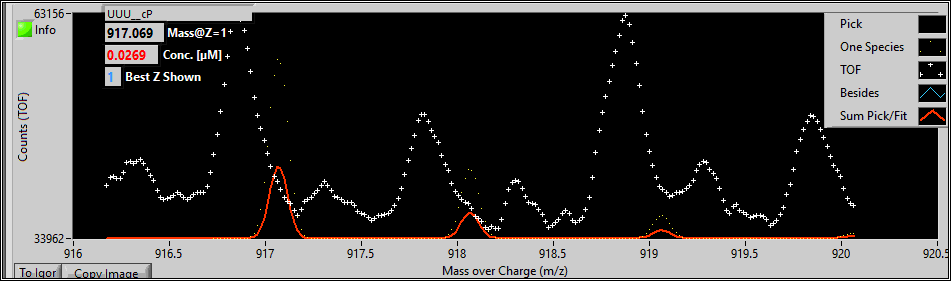

Supplement: Supplementary file 3 — Supplementary Data 1, 2 and 3 [file 41467_2025_60359_MOESM3_ESM.zip › Supplementary Data/Supplementary Data 3/AU-no aa/000013_UUU__cP.bmp]

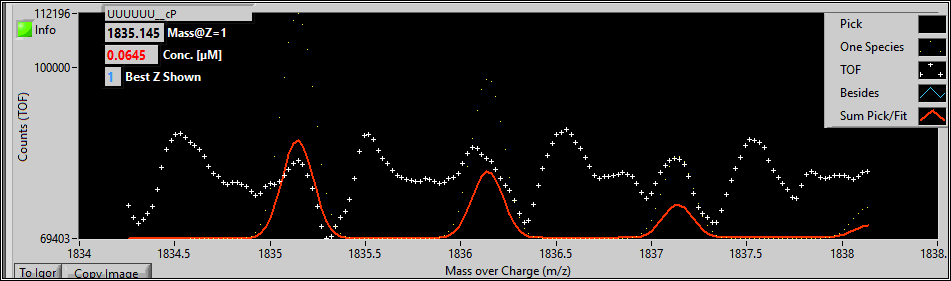

Supplement: Supplementary file 3 — Supplementary Data 1, 2 and 3 [file 41467_2025_60359_MOESM3_ESM.zip › Supplementary Data/Supplementary Data 3/AU-no aa/000049_UUUUUU__cP.bmp]

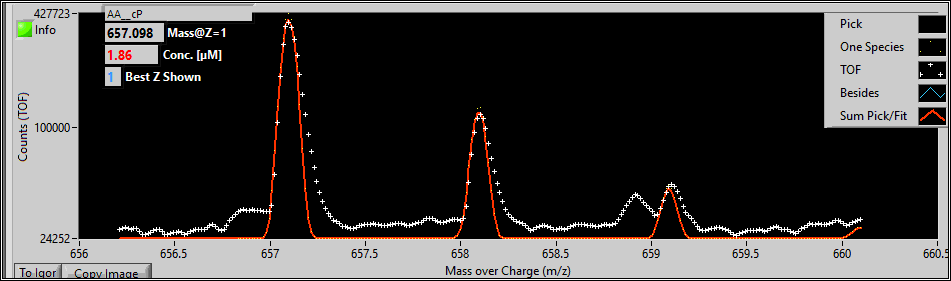

Supplement: Supplementary file 3 — Supplementary Data 1, 2 and 3 [file 41467_2025_60359_MOESM3_ESM.zip › Supplementary Data/Supplementary Data 3/AU-no aa/000001_AA__cP.bmp]
